# Mapping child growth failure in Africa between 2000 and 2015

**DOI:** 10.1038/nature25760

**Published:** 2018-03-01

**Authors:** Aaron Osgood-Zimmerman, Anoushka I. Millear, Rebecca W. Stubbs, Chloe Shields, Brandon V. Pickering, Lucas Earl, Nicholas Graetz, Damaris K. Kinyoki, Sarah E. Ray, Samir Bhatt, Annie J. Browne, Roy Burstein, Ewan Cameron, Daniel C. Casey, Aniruddha Deshpande, Nancy Fullman, Peter W. Gething, Harry S. Gibson, Nathaniel J. Henry, Mario Herrero, L. Kendall Krause, Ian D. Letourneau, Aubrey J. Levine, Patrick Y. Liu, Joshua Longbottom, Benjamin K. Mayala, Jonathan F. Mosser, Abdisalan M. Noor, David M. Pigott, Ellen G. Piwoz, Puja Rao, Rahul Rawat, Robert C. Reiner, David L. Smith, Daniel J. Weiss, Kirsten E. Wiens, Ali H. Mokdad, Stephen S. Lim, Christopher J. L. Murray, Nicholas J. Kassebaum, Simon I. Hay

**Affiliations:** 1 Institute for Health Metrics and Evaluation, University of Washington, Seattle, Washington 98121, USA; 2 Department of Infectious Disease Epidemiology, Imperial College London, London SW7 2AZ, UK; 3 Big Data Institute, Li Ka Shing Centre for Health Information and Discovery, University of Oxford, Oxford OX3 7FZ, UK; 4 Commonwealth Scientific and Industrial Research Organisation, St Lucia, Queensland 4067, Australia; 5 Bill & Melinda Gates Foundation, Seattle, Washington 98109, USA; 6 Kenya Medical Research Institute-Wellcome Trust Collaborative Programme, Nairobi, Kenya; 7 Centre for Tropical Medicine and Global Health, Nuffield Department of Clinical Medicine, University of Oxford, Oxford OX3 7FZ, USA; 8 Department of Anesthesiology and Pain Medicine, Seattle Children’s Hospital, Seattle, Washington 98105, USA

## Abstract

Insufficient growth during childhood is associated with poor health outcomes and an increased risk of death. Between 2000 and 2015, nearly all African countries demonstrated improvements for children under 5 years old for stunting, wasting, and underweight, the core components of child growth failure. Here we show that striking subnational heterogeneity in levels and trends of child growth remains. If current rates of progress are sustained, many areas of Africa will meet the World Health Organization Global Targets 2025 to improve maternal, infant and young child nutrition, but high levels of growth failure will persist across the Sahel. At these rates, much, if not all of the continent will fail to meet the Sustainable Development Goal target—to end malnutrition by 2030. Geospatial estimates of child growth failure provide a baseline for measuring progress as well as a precision public health platform to target interventions to those populations with the greatest need, in order to reduce health disparities and accelerate progress.

## Abstract (web summary)

Geospatial estimates of child growth failure in Africa provide a baseline for measuring progress and a precision public health platform to target interventions to those populations with the greatest need.

Child undernutrition increases the risk of neonatal and child mortality and future maternal reproductive outcomes^1^,^2^,^3^. Child growth failure (CGF) is the specific subset of child undernutrition, excluding micronutrient deficiencies, that is characterized by the relationship between insufficient height and weight at a given age, and this subset is most universally described in terms of univariate ‘growth standards’, for which age-specific heights and weights are compared to healthy reference populations^4^,^5^. In aggregate, univariate assessments of stunting, wasting and underweight (Extended Data Fig. 1) can serve as a comprehensive assessment of CGF. Prevalence of moderate and severe stunting, wasting and underweight among children aged 0–59 months is defined as the proportion of children with a height-for-age, weight-for-height or weight-for-age *z* score that is more than two standard deviations below the 2006 WHO (World Health Organization) growth reference population, respectively^4^.

The Millennium Development Goals (MDG) had a single nutrition target: a 50% reduction in prevalence of underweight in children under five between 1990 and 2015. In 2012, WHO member states endorsed a broader agenda to improve nutrition by 2025: the Global Nutrition Targets (WHO GNT), including stunting, wasting, low birth weight and overweight^6^ in children under five (see Extended Data Fig. 1). Sustainable Development Goal (SDG) 2.2 is even more aspirational, calling for an end to all forms of malnutrition by 2030, progress towards which can be seen as inseparable from many of the other SDG child health ambitions^7^,^8^,^9^.

Quantitative assessments of levels and trends in CGF indicators serve as key input to discussions of progress and areas for improvement^1^,^10^,^11^,^12^,^13^,^14^. According to findings from the Global Burden of Diseases, Injuries, and Risk Factors Study 2016 (GBD 2016), an estimated 36.6% of children under five were stunted, 8.6% wasted and 19.5% underweight in sub-Saharan Africa (SSA) in 2015^1^. Furthermore, CGF was the second leading risk factor for child mortality in SSA, accounting for more than 23% of deaths of children under five in this region^1^.

## Precision public health and child growth failure

Although country-level estimates are useful for international comparisons and benchmarking, they mask disparities in CGF at the lower administrative levels at which most health and nutrition policy planning and implementation occur. The value of precision public health in this context—the use of more spatially resolved data to guide efficient targeting of interventions to those populations with the greatest need—is increasingly recognized by the global health community^15^. This approach enables quantification of inequalities and identification of successes and failures of programmes and policies at the local level. Similar efforts that mapped subnational malaria prevalence, incidence, and mortality^16^,^17^ have, when overlaid with interventions, shown where use of insecticide-treated nets or access to treatment is lacking, pinpointing where remedial actions are needed. Without comparable, robust subnational information on stunting, wasting and underweight, health authorities face sizeable challenges to precisely target and thus optimally fund relevant CGF interventions.

Subnational assessments of CGF have been conducted in select countries in Africa, including states in Nigeria^18^, regions in Uganda^19^, governorates in Egypt^20^ and districts in Ethiopia^21^, Malawi^22^,^23^, Tanzania^23^ and Zambia^23^, as well as the Demographic and Health Surveys, which report at the first administrative subdivision in 39 countries^24^. Although this initial work has unveiled coarse subnational disparities in CGF, it provides an incomplete picture, with heterogeneity remaining within administrative units. Model-based geostatistics, a set of statistical techniques developed to make inferences from spatially correlated phenomena, have produced high-spatial-resolution estimates of nutrition indicators in Burkina Faso^25^, Ghana^25^, Kenya^26^, Mali^25^, Nigeria^26^, Tanzania^26^ and Somalia^27^. These studies demonstrate that geo-referenced anthropometric survey data, if properly harnessed with spatially and temporally explicit models and appropriate covariates, can allow for the synthesis of these data into gridded maps. However, a sizable geographical knowledge gap remains, as the combined analyses of previous studies are not comprehensive or generalizable. Furthermore, advances in data sharing and computational statistics enable high-resolution estimates to be made over continental and global scales^16^,^17^.

Here we provide a comprehensive geospatial analysis of CGF in 51 African countries from 2000 to 2015, offering highly relevant subnational information on key nutrition indicators for policymakers and health practitioners at all administrative subdivisions. We used Bayesian model-based geostatistics, which uses geo-referenced child anthropometry survey data and gridded covariates over space and time, in an ensemble modelling framework based on stacked generalizations^28^ and spatial validation processes, to produce 5 × 5 km gridded estimates of stunting, wasting, and underweight for children under five. To ensure comparability with national estimates and to facilitate benchmarking, we calibrated pixel-level estimates to those produced by GBD 2016^1^ and subsequently aggregated 5 × 5 km estimates to multiple administrative subdivisions in each country. We compared the annualized rate of change (AROC) for each CGF measure during the MDG era (2000 to 2015) relative to the AROC required between 2015 and 2025 to meet the WHO GNT (Figs 1g, 2g, Extended Data Fig. 2g), and the acceleration in the pace of progress required between 2015 and 2025 to achieve the WHO GNT (Figs 1i, 2i, Extended Data Fig. 2i).

## Disparate progress in reducing child growth failure

Between 2000 and 2015, nearly all African countries showed a reduction in the absolute levels of stunting, wasting and underweight in children under five, but observed rates of change varied markedly^1^,^2^,^10^,^29^. If current rates of progress continue, many countries are on track to meet the relevant WHO GNT^6^ at the national level. This includes most of eastern and southern SSA and the coastal sections of western SSA at more local scales. However, our results also show particularly high levels of CGF, with little evidence of improvement, across the Sahel.

Stunting (Extended Data Fig. 1a) was the most prevalent form of CGF across all years, and its change in prevalence across time was visually striking (Fig. 1a–c). While large areas of Algeria, Mozambique, Burkina Faso and Ghana showed a reduction in the prevalence of stunting from 2000 to 2015, progress in other countries was more spatially heterogeneous. Progress occurred between 2005 and 2015 in many areas, as illustrated by the Imo state in Nigeria, in which the mean estimated stunting prevalence was nearly halved (46.2% reduction; 95% uncertainty interval, 38.7–54.9%) from 31.5% (28.1–35.5%) in 2005 to 16.9% (14.7–19.2%) in 2015. By 2015, lower levels were found in coastal central Africa, particularly in areas within Ghana, Gabon and Equatorial Guinea. By contrast, northern Nigeria, southern Niger, Democratic Republic of the Congo (DRC), Zimbabwe and northern Mozambique all had areas with a prevalence of stunting near or above 40% in 2000, which was as high as 64.9% (59.3–70.8) in the Lubango municipality within Huila province, Angola. Although many of these regions showed improvement (the prevalence rate in Lubango dropped to 31.5% (27.2–35.9%) in 2015), some areas, such as regions of the Northern Province of Zambia, northern Nigeria, and southern Niger, had the highest prevalence rates in both 2000 and 2015.

Wasting (Extended Data Fig. 1b) is a short-term phenomenon that encompasses both moderate acute malnutrition and severe acute malnutrition^4^. Wasting is more sensitive to external environmental fluctuations, such as crop yields and food availability^30^, and is most likely to affect children over the course of months, rather than years. These shorter-term events drive uneven temporal patterns of decline compared to the more consistent decreases seen in stunting and underweight. As such, some areas in northern Kenya, eastern Ethiopia, northern Nigeria and Madagascar show temporal variation and increases across the study years (Fig. 2a–c). The Afar region in Ethiopia, for example, had a high prevalence in both 2000 (16.7% (14.5–19.4%)) and 2015 (21.7% (18.9–24.7%)). While the estimated prevalence in regions of Madagascar also increased, these estimates were relatively uncertain (Fig. 2f). High prevalence of wasting appears in a band across the continent, with concentrations in Niger (19.9% (18.9–20.9%)), South Sudan (21.0% (18.0–24.2%)) and Burkina Faso (18.9% (17.9–19.9%)) in 2000. Foci of higher prevalence remain even in countries with low rates nationally. Kenya, for example, had a national prevalence of 5.7% (5.2–6.2%) in 2015, although rates as high as 28.2% (24.8–31.8%) were found in areas within the Rift Valley province. Prevalence of wasting in southern Africa, by contrast, remained consistently low across the study period. Some countries, including the DRC, experienced sizeable progress both nationally and subnationally, dropping from 14.6% (13.9–15.4%) in 2000, with rates as high as 18.4% (16.7–20.2%) in the Équateur province, to 8.7% (8.2–9.2%) in 2015, with a decrease in Équateur to 9.8% (8.7–11.0%), lessening the gap between national and subnational prevalence.

Of particular note when comparing the prevalence of underweight (Extended Data Fig. 1c) across time and space, is the persistent band of high prevalence across the Sahel, stretching from southern Mali in the west to the Horn of Africa in the east (Extended Data Fig. 2a–c). By contrast, for the northern coast of Africa, in countries near the Gulf of Guinea, and in southern Africa, prevalence remained low throughout the study period, achieving rates such as 3.8% (2.7–5.2%) in the Litoral province of Equatorial Guinea by 2015. Patterns of change in central Africa were highly spatially heterogeneous in countries, such as Nigeria, which achieved rates below 10% in some regions, and in excess of 30% in northern areas by 2015. Marked progress was seen in central Africa, with Rwanda reducing national prevalence from 22.3% (20.7–23.8%) in 2000 to 8.7% (7.8–9.6%) in 2015. Although Angola and the DRC have experienced substantial improvement, hot spots remain, such as the Kasai-Occidental province in the DRC, in which prevalence was 25.3% (22.9–27.9%) in 2015.

For each CGF metric, Figures 1e, 2e and Extended Data Figure 2e show estimates of the population-weighted highest and lowest 10% of pixels across the continent in 2000 and 2015, as well as their overlap. Overlaid stippling across the continent represents areas in the estimated maps that experienced the 10% lowest and highest rates of decline for the 16 years that were modelled. Areas in Angola (Fig. 1e) experienced some of the highest rates of stunting in 2000, but also some of the highest annualized rates of decline; by 2015, pixels in that same area were no longer in the worst 10%. Conversely, as demonstrated for southern Niger and northern Zambia, where some of the highest stunting rates in both 2000 and 2015 occurred, these maps can elucidate places and populations left behind as the continent progresses towards the WHO GNT.

Figures 1f, 2f and Extended Data Fig. 2f show our estimates contrasted with their respective certainty for each 5 × 5-km area in 2015. These maps more intuitively highlight areas for which our estimates are less uncertain, and the corresponding relative prevalence of the CGF indicator. For example, much of Zimbabwe had a low prevalence of wasting (3.8% (3.4–4.1%)) at the national level and had low uncertainties relative to other areas. Areas in Chad (such as the Kanem region, with a prevalence of stunting of 50.0% (47.0–52.9%)) had a high prevalence and were relatively certain. By contrast, the Melaky region of Madagascar experienced a high prevalence of wasting, but estimates in those areas were relatively uncertain (42.2% (28.3–58.0%)). For more detail, see Supplementary Figs 13–15.

The predicted space–time models of CGF prevalence closely matched the observed national survey data, and we used 5-fold cross-validation strategies to assess the fit of our models. The full array of validation metrics by indicator and country are provided in the Supplementary Information (Supplementary Tables 8–19 and Supplementary Figs 16–36).

Given the continental scope and fine spatial scale of this work, additional results are provided in the Supplementary Information and all outputs of these analyses at the first administrative subdivision (for example, state), second administrative subdivision (for example, district), and 5 × 5-km levels are publicly available in the Global Health Data Exchange (http://ghdx.healthdata.org/record/africa-child-growth-failure-geospatial-estimates-2000-2015) and via bespoke data visualization tools (https://vizhub.healthdata.org/lbd/cgf).

## Outlook for the 2025 GNT

Progress towards the WHO GNT has been uneven (Figs 1g, 2g and Extended Data Fig. 2g). The AROC between 2000 and 2015 relative to the pace required to meet the WHO GNT by 2025 clearly shows that some areas are on track or exceeding the pace required to achieve the targets and other locations appear to have already met the goal. Yet there are still vast expanses of the continent that must increase their rate of progress two-, three-, and even fourfold to achieve the WHO GNT by 2025 (Figs 1i, 2i and Extended Data Fig. 2i). Inland areas of central Africa and the Sahel will require the most marked improvement, with many areas requiring at least two- to fourfold increases in their annual rates of decrease across CGF indicators. Stunting targets—40% reduction by 2025—are unlikely to be met in many areas of central Africa without an accelerated rate of decline, while some areas, such as southern and western coastal Africa, are on target to meet the WHO GNT. The reduction and maintenance of child wasting to less than 5% is likely to be met in most countries in southeastern Africa based on current trajectories, but much of central SSA and the entire Sahel will require pronounced improvements in order to meet 2025 targets.

The likelihood of areas meeting the WHO GNT is sensitive to the spatial scale used to measure achievement. In Fig. 3, the probability that regions have reached the WHO GNT in 2015 is estimated at the first administrative subdivision, as well as at the 5 × 5-km pixel level, showing the increased nuance in the results gained at finer spatial scales. For example, the administrative boundaries of Kenya are aligned in a north–south orientation, cutting across areas of low and high prevalence, which generates population-weighted probabilities of having met the stunting target between 0% and 50% for much of eastern Kenya (Fig. 3a). Viewing the same results at a 5 × 5-km resolution (Fig. 3b) shows that pockets within Kenya had a much higher (over 95% in some places) probability of meeting the target in 2015.

## Discussion

Our study provides a quantification of CGF in 51 African countries at a 5 × 5 km spatial resolution, highlighting a mixture of impressive gains and enduring disparities in CGF within countries and across the continent. By 2015, nearly all locations showed decreases in the rates of stunting, wasting and underweight compared to 2000, with noticeable tracts of the DRC, Mozambique, Angola and Burkina Faso showing considerable reductions in multiple CGF indicators, despite room for improvement within these locales (Figs 1g, 2g and Extended Data Figs 2g, 3, 4). Conversely, some countries are performing poorly across wide areas in all CGF indicators: South Sudan, Chad, Ethiopia, Madagascar, Sudan and northern Nigeria had some of the highest rates of CGF based on multiple metrics in 2015 (Extended Data Fig. 4). We also provide a baseline assessment of the WHO GNT for CGF at the local level for 2015 (Fig. 3), to guide policy and precision public health interventions to improve outcomes by 2025. Although policies are often set at administrative levels, implementation happens locally, such as within districts or cities, particularly when targeting specific at-risk populations or studying responses to interventions over time. Our analysis at the 5 × 5-km level enables the identification of programme and policy successes and failures at the local level and quantification of inequalities to guide efficient targeting of resources and interventions to those populations with the greatest need.

Differences between alternative sets of international nutrition targets must be acknowledged, along with the fact that even in high-income nations, malnutrition has not been entirely eliminated. MDG 1.C called for a 50% reduction in underweight prevalence between 1990 and 2015. On the basis of national-level GBD results, 21 African countries achieved MDG 1.C and an additional 16 surpassed the achievement rate from 2000 to 2015^1^. The mapping of 5 × 5 km levels and trends in underweight has revealed substantial further geographical heterogeneity during the MDG period (Extended Data Fig. 2h), with almost every country having areas in which improvements were consistent with achieving MDG 1.C, and every country having areas in which improvement lagged behind the national rate. The WHO GNT were formulated by analysing national CGF trends in Brazil, China, Bangladesh and Mexico, all of which made remarkable progress during the MDG period^12^. While most of Africa must accelerate reductions in CGF in children under 5 in order to meet the WHO GNT by 2025, these aspirational goals are anchored in examples of past achievement. By contrast, the current wording of SDG 2.2 calling to ‘end all forms of malnutrition’ is clinically vague and almost certainly unachievable. There is a need for more clearly defined SDG targets for malnutrition and CGF, formulated in terms of absolute, rather than relative change. Absolute targets would bring SDG 2.2 in line with the overall aim of the SDGs of achieving a ‘grand convergence’ in health^31^.

Most CGF improvements in Africa occurred after the year 2000, and were likely catalysed by large political, social and financial investments^32^. A number of related factors are likely to have led to improvements in nutrition, CGF and child mortality^4^. These include general sociodemographic improvements^9^ and broad scaling up of interventions that focused on reducing childhood illness, such as malaria control, vaccination coverage, HIV prevention and treatment, and water, sanitation and hygiene facilities, which can break the cycle of metabolic compromise leading to CGF^9^,^16^,^17^,^33^,^34^,^35^. It is probably no coincidence that many of the nations and regions with slower gains, such as Central African Republic, Chad, Somalia and much of the Sahel, received less international assistance for newborn and child health^26^, had persistently low coverage of sentinel maternal and child health interventions^36^, experienced periods of pronounced conflict^36^,^37^, and showed no progress in their sociodemographic index status^38^. The finding of a continued high burden of wasting in arid sections of the Sahel, the Horn of Africa and sections of southern SSA is especially important given the implications of famine on potential for human health, geopolitical unrest and mass migration^39^,^40^. There is strong correspondence between the areas of high prevalence of wasting in 2015 (Fig. 2c) and the nations (Nigeria, South Sudan, Somalia, and Yemen (not mapped here)) that were identified by the United Nations as collectively containing approximately 20 million people that are at imminent risk of famine^41^.

We estimate that no country in Africa is likely to achieve all of the WHO GNT in all of its territory if current trends continue, highlighting a widespread need to adopt evidence-based, precision public health programmes to track and improve progress. In an era of static development assistance for health^32^ and in countries where financial resources are constrained, highly localized mapping of CGF may facilitate more efficient stewardship by providing a way to pair vulnerable communities with health and nutrition programmes, community support and knowledge that are more likely to meet their specific needs. Targeting precision health interventions to reduce the burden of CGF without considering key sociodemographic factors poses large risks to the sustainability of intervention strategies, either directly through unrealistic assumptions about care-seeking behaviour and retention, or indirectly by not working in tandem to break cycles of poverty and mitigate CGF risk for future generations. Geospatial estimates of average, community-level human capital, such as those provided in the complementary mapping of educational attainment in Africa^42^, should be considered when striving to make policy decisions at a local level. The exact combination of intervention packages required for remedial action to combat CGF was not directly addressed in this study. Further context on the diverse range of instruments and interventions to address CGF is provided in the Supplementary Discussion.

## Future work and caveats

Our present study offers the analytic framework from which we aim to extend geospatial modelling of CGF to all low- and middle-income countries, with a heightened focus on the modelling of holistic measures of CGF, such as the composite index of anthropometric failure^43^. These more integrated measures would take into consideration the overlapping and longitudinal influences of being born early, born small and having an early childhood characterized by inadequate height or weight gain. To provide a complete baseline and assessment of progress towards all six WHO GNT, we plan to expand our analysis to include mapping of low birth weight, childhood overweight, anaemia in women of reproductive age, and exclusive breastfeeding in the first six months of life.

The accuracy of this work is primarily determined by the volume and fidelity of nationally representative surveys, regardless of the sophistication of the models used. The limitations of these data, including collection biases in anthropometric measurement and non-existent data on deceased children, underscore the need for future refinement and improved data collection. Furthermore, the statistical model does not yet incorporate child-level covariates, which may mask sub-pixel heterogeneity across sex, age and socio-economic factors (see Methods for additional detail on methodological limitations).

National improvements in CGF across Africa may mask large subnational and acute 5 × 5-km grid-level variation, such that no country in SSA has reached the relevant WHO GNT or SDG targets in all of its territory, or is projected to do so by 2025 or 2030, respectively, under current rates of improvement. As researchers, policymakers and programme implementers continue to determine the optimal mix of interventions to alleviate CGF, they now have at their disposal a precision public health tool to monitor subnational inequalities and target interventions to those populations with the greatest need.

## Methods

### Overview

Our study follows the Guidelines for Accurate and Transparent Health Estimates Reporting (GATHER). Our analysis provides estimates of the prevalence of stunting, wasting and underweight in children under 5 (Extended Data Fig. 1) based on univariate growth standards for which age-specific height and weight are benchmarked against children of the same age from healthy reference populations^4^,^5^. Stunting, wasting and underweight are defined as *z* scores that are two or more standard deviations below the reference median for height-for-age (HAZ), weight-for-height (WHZ) and weight-for-age (WAZ), respectively. Our primary goal is to provide prevalence predictions across the African continent at a high resolution and we have used methods to provide the best out-of-sample predictive performance at the expense of inferential understanding. We modelled prevalence of each indicator on a 5 × 5-km grid over 51 countries in Africa at an annual resolution from 2000 to 2015. This includes all 48 countries in mainland Africa, as well as islands for which we had survey data, including Madagascar, Comoros, and São Tomé and Príncipe. We do not estimate for island nations for which no available survey data could be sourced, including Mauritius, Seychelles and Cape Verde. After harmonizing the data, we implemented an ensemble modelling framework that feeds into a Bayesian generalized linear model with a correlated space–time error. We took 1,000 draws from the fitted posterior distribution and we combined and processed the draws into 1,000 candidate 5 × 5-km resolution maps that were used to generate all of our final results. The analytical steps and their limitations are described in detail below and additional detail can be found in the Supplementary Information.

### Data

We extracted individual-level height, weight and age data for children under 5 from household survey series, including the Demographic and Health Surveys (DHS), Multiple Indicator Cluster Surveys (MICS), Living Standards Measurement Study and Core Welfare Indicators Questionnaire (CWIQ), among other country-specific child health and nutrition surveys^24^,^50^,^51^,^52^. Each individual record is associated with a cluster, a group of neighbouring households or a ‘village’ that acts as a primary sampling unit. Some surveys include geographical coordinates or precise place names for each cluster within that survey (50,142 clusters for stunting, 49,564 for wasting and 50,078 for underweight). In the absence of geographical coordinates; coordinates for each cluster, we assigned data to the smallest available administrative areal unit in the survey while correcting for the survey sample design^53^,^54^. Boundary information for these administrative units was obtained as shapefiles either directly from the surveys or by matching to shapefiles in the Global Administrative Unit Layers^44^ database or the Database of Global Administrative Areas^55^. For select cases, shapefiles provided by the survey administrator were used or custom shapefiles were created on the basis of the survey documentation. These areal data were resampled to 10,000 coordinate locations per areal observation using a population-weighted sampling scheme over the relevant area^49^. *k*-means clustering on the sampled locations reduces the sampled points to a set of *k*-means centroids acting as proxies for community locations, and the number of points in each cluster informs the weighting given to the point. These centroids are taken to be the geolocations for the observation, and the pseudo-observations are down-weighted in the likelihood evaluation to account for our uncertainty in the precise location of the observation. Weighting by sample size, GPS-located clusters contributed at least 47.4% of the total data per indicator, and resampled areal data contributed the remainder. Extended Data Figures 5, 6 show stunting data availability by type and country from 2000 to 2015. Wasting and underweight data availability can be found in Supplementary Figs 2, 3.

#### Child anthropometry data

Using the height, weight and age data for each individual, HAZ, WHZ and WAZ were calculated using the age-, sex- and indicator-specific LMS values from the 2006 WHO Child Growth Standards, which takes into account distributional skew using the lambda parameter, the centre of the distribution using the mu parameter, and the spread of the distribution using the sigma parameter^4^,^5^. The LMS methodology allows for Gaussian *z* score calculations and comparisons to be applied to skewed, non-Gaussian distributions^56^. These microdata were then collapsed to cluster-level or areal-level prevalence of moderate stunting, wasting and underweight (HAZ < −2s.d., WHZ < −2s.d. and WAZ < −2s.d. below the reference median, respectively). Data from the Somalia Food Security and Nutrition Analysis Unit were provided as already collapsed to cluster-level prevalences (using the WHO 2006 standards).

#### Data exclusion criteria

Select data sources were excluded for the following reasons: missing survey weights for areal data, missing gender variables, insufficient age granularity (in months) for HAZ and WAZ calculation in children aged 0–2 years, incomplete sampling (for example, only children aged 0–3 years measured), or untrustworthy data (as determined by the survey administrator or by user inspection). Within each source, polygon survey clusters with a sample size of one were excluded. Untrustworthy data refer specifically to the exclusion of six surveys for the reasons described here. Two datasets, the 2009–2010 Ghana Socioeconomic Panel Survey and the 2005 Burkina Faso CWIQ, were excluded because the national prevalence values reported for one or more indicators were determined to be implausibly high based on the country-level trend seen in the seven other Ghana and six other Burkina Faso sources. In addition, the data were only resolved to the first administrative subdivision. This combined with the very coarse spatial resolution makes the data of minor use for our geospatial purposes. Two additional sources, the 2014 MICS Kenya Kakamega and Bungoma surveys, were excluded because, according to the survey documentation, the ‘anthropometric data suffered from digit preference for both weight and height’, meaning the measurements were rounded with preference for certain numbers in a way that introduced considerable bias. The 2015 Ethiopia Living Standards Measurement Study–Integrated Surveys on Agriculture was excluded, because the low prevalence of child growth failure in the Ogaden region was determined to be unrealistic by specialists in the field of child nutrition. Lastly, the 2015 Egypt Special DHS was excluded because of the non-proportional sample allocation that was designed to estimate the prevalence of hepatitis and certain other non-communicable disease risk factors such that the survey sampling was not equivalent to the rest of the surveys.

#### Temporal resolution

We estimated prevalence of stunting, wasting and underweight annually from 2000 to 2015 using a model that allows us to account for data points that were continuously measured over time. As such, the model would also allow us to predict at monthly or finer temporal resolutions. However, we are computationally limited by the temporal resolution of our space–time covariates. In order to account for seasonality within each year of observations, periodic splines were fitted to the data by regions defined by GBD^57^ (Extended Data Fig. 2).

#### Seasonality adjustment

Owing to the acute nature of wasting and its relative temporal transience, wasting data were pre-processed to account for seasonality within each year of observation. Generalized additive models (GAMs) were fitted to wasting data across time using the month of interview and a country-level fixed effect as the explanatory variables and WHZ as the response. A 12-month periodic spline for the interview month was used, as well as a spline that smoothed across the whole duration of the dataset and country-level random effects. The GAMs were fitted to the data by regions defined by GBD^57^ (Extended Data Fig. 7) in order to allow for different seasonality adjustments across the continent^57^. Once the models were fitted, individual WHZ observations were adjusted, using only the fit from the periodic spline, so that each measurement was consistent with a day that represented a mean day in the periodic spline. The seasonality adjustment introduced relatively little change to the raw data. This analysis could not be run on sources missing interview dates, which were excluded from the wasting data. See Supplementary Information for more detail and the adjustment is shown in Supplementary Figs 5, 6.

#### Spatial covariates

In order to leverage strength from locations with observations to the entire spatiotemporal domain, we compiled several 5 × 5-km raster layers of possible socio-economic and environmental correlates of CGF in Africa (see Supplementary Table 3 and Supplementary Fig. 4). These covariates were selected on the basis of their potential to be predictive for the set of CGF indicators, after reviewing literature on evidence and plausible hypotheses as to their influence. Acquisition of temporally dynamic datasets, where possible, was prioritized in order to best match our observations and thus predict the changing dynamics of the CGF indicators. Of the 37 covariates included, 23 were temporally dynamic and were reformatted as a synoptic mean over each estimation period or as a mid-period year estimate. The remaining 14 covariate layers were static, and were applied uniformly across all modelling years. Furthermore, we also used a number of covariates that are constant within each country and year: the percentage of population with access to improved toilet types, and per capita lag distributed income, as indicated as predictive of CGF in GBD 2016^1^. Country-level age-standardized mortality rates due to famine as produced by GBD 2016 were also included in the model for wasting. More information, including plots of all covariates, can be found in the Supplementary Information.

An ensemble covariate modelling method was implemented in order to both select covariates and capture possible nonlinear effects and complex interactions between them^28^. For each region, three sub-models were fitted to our dataset, using all of our covariate data as explanatory predictors: GAMs, boosted regression trees and lasso regression. Each sub-model was fitted using fivefold cross-validation to avoid overfitting, and the out-of-sample predictions from across the five holdouts were compiled into a single comprehensive set of predictions from that model. Additionally, the same sub-models were also run using 100% of the data and a full set of in-sample predictions were created. The five sets of out-of-sample sub-model predictions were fed into the full geostatistical model as the explanatory covariates when performing the model fit. The in-sample predictions from the sub-models are used as the covariates when generating predictions using the fitted full geostatistical model. A recent study has shown that this ensemble approach can improve predictive validity by up to 25% over an individual model^28^. More details on the ensemble covariate modelling can be found in the Supplementary Methods and example predictive rasters can be found in Supplementary Fig. 11.

### Analysis

#### Geostatistical model

Binomial count data are modelled within a Bayesian hierarchical modelling framework using a logit link function and a spatially and temporally explicit hierarchical generalized linear regression model to fit prevalence of each of our indicators in five regions of Africa as defined in GBD^57^ (‘Northern’, ‘Western’, ‘Southern’, ‘Central’, and ‘Eastern’; see Extended Data Fig. 7). The GBD study design sought to create regions on the basis of two primary criteria: epidemiological homogeneity and geographic contiguity^57^ (see Extended Data Fig. 7). For each GBD region, we explicitly write the hierarchy that defines our Bayesian model as follows:
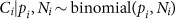

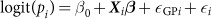

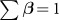

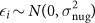

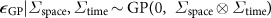

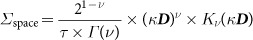

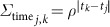


For each indicator and region, we modelled the number of children at cluster *i*, among a sample size, ***N***_***i***_, who are subject to the indicator as binomial count data, *C*_*i*_. We have suppressed the notation, but the counts (*C*_*i*_), probabilities (*p*_*i*_), predictions from the three submodels (*X*_*i*_) and residual terms 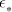 are all indexed at a space–time coordinate. The probabilities (*p*_*i*_) represent both the annual prevalence at the space–time location and the probability that an individual child will be afflicted with the risk factor given that they live at that particular location. The logit of annual prevalence (*p*_*i*_) of our indicators was modelled as a linear combination of the three sub-models (GAM, boosted regression trees and lasso regression), ***X***_****i****_, a correlated spatiotemporal error term (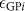) and an independent nugget effect, 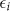. Coefficients (***β***) on the sub-models represent their respective predictive weighting in the mean logit link and are constrained to sum to 1. In order for this constraint to make any sense, we ensure that the predictions from the sub-models entered into INLA (integrated nested Laplace approximation)^28^ in the link space (logit) without having been centre-scaled. The joint error term (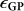) accounts for residual spatiotemporal autocorrelation between individual data points that remains after accounting for the predictive effect of the sub-model covariates, and the nugget (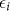), which is an independent error term for each data point, representing irreducible error for that observation. The residuals (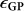) are modelled as a three-dimensional Gaussian process in space–time centred at zero and with a covariance matrix constructed from a Kronecker product of spatial and temporal covariance kernels. The spatial covariance (*Σ*_space_) is modelled using an isotropic and stationary Matérn function^58^, and temporal covariance (*Σ*_time_) as an annual autoregressive-order-1 function over the 16 years that are represented in the model. This approach leveraged the residual correlation structure of the data to more accurately predict prevalence estimates for locations with no data, while also propagating the dependence in the data through to uncertainty estimates^59^. The posterior distributions were fitted using computationally efficient and accurate approximations in R INLA^60^,^61^ with the stochastic partial differential equations^62^ approximation to the Gaussian process residuals. Pixel-level uncertainty intervals were generated from 1,000 draws (that is, statistically plausible candidate maps)^63^ created from the posterior-estimated distributions of modelled parameters. Additional detail on the geostatistical model and estimation process can be found in the Supplementary Methods.

To transform pixel-level estimates into a range of information useful for a wide community of potential users, these estimates were aggregated from the 1,000 candidate maps up to the second administrative subdivision, the first administrative subdivision and national levels using population weighted conditional simulation^64^. This aggregation also enabled calibration of estimates to national GBD 2016^1^ estimates for 2000, 2005, 2010 and 2015. More details on the calibration can be found in the ‘Post estimation’ section.

Although the model can predict all locations covered by available raster covariates, all final model outputs for which land cover was classified as ‘barren or sparsely vegetated’ were masked on the basis of the most recently available MODIS satellite data (2013), as well as areas where the total population density was less than ten individuals per 1 × 1-km pixel in 2015. This step has led to improved understanding of the maps when communicating with data specialists and policymakers.

#### Post estimation

To leverage national-level data included in GBD 2016, but outside the scope of our current geospatial modelling framework, and to ensure perfect calibration between these estimates and GBD 2016 national-level estimates, we performed a post hoc calibration to each of our 1,000 candidate maps^1^. For each posterior draw, we calculated population-weighted pixel aggregations to a national level and compared these country–year estimates to the analogous and available GBD 2016^1^ country–year estimates (all countries for 2000, 2005, 2010 and 2016). To generate 2015 national-level estimates for use in calibrating our 2015 5 × 5-km maps, we linearly interpolated between 2010 and 2016 estimates. We defined the raking factor to be the ratio between the GBD 2016^1^ estimate and our current estimates and linearly interpolated raking factors in a country between the available years yielding raking factors for all country–year pairs. Finally, we multiplied each of our pixels in a country–year pair by its associated raking factor. This ensures perfect calibration between our geospatial estimates and GBD 2016^1^ national-level estimates, while preserving our estimated within-country geospatial and temporal variation.

The median for the raking factor ratios across all three indicators was 0.999 (interquartile range, 0.920–1.096), indicating a very close agreement with GBD 2016^1^ estimates. Scatter plots comparing national-level estimates from this analysis with GBD 2016^1^ estimates can be found in Supplementary Figs 40–42.

#### Model validation

Models were validated using spatially stratified fivefold out-of-sample cross-validation. In order to offer a more stringent analysis by respecting some of the spatial correlation in the data, holdout sets were created by combining sets of spatially contiguous data at different spatial resolutions, for example, the first administrative subdivision. Validation was performed by calculating bias (mean error), total variance (root-mean-square error) and 95% data coverage within prediction intervals, and correlation between observed data and predictions. All validation metrics were calculated on the out-of-sample predictions from the fivefold cross-validation. We compared five different model formulations (stacked ensemble with and without space–time error, raw satellite covariates with and without space–time error, and the Gaussian process space–time error without any covariates) using out-of-sample predictive metrics. The results are presented in the model validation section of the Supplementary Methods, in which we show that using the stacked ensemble covariates in conjunction with the space–time error consistently outperforms the other models across all three indicators.

Where possible, results from these models were compared against other existing estimates, such as subnational DHS estimates as shown in Supplementary Fig. 43. Furthermore, measures of spatial and temporal autocorrelation pre- and post-modelling were examined to verify correct recognition, fitting and accounting for the complex spatiotemporal correlation structure in the data. We found our in-sample-size weighted Pearson’s correlation between our posterior mean predictions at data observation locations and the observed prevalence proportions to be 0.70, 0.66 and 0.76 for stunting, wasting and underweight, respectively, at the pixel level, and 0.98, 0.96 and 0.99, respectively, at the national level. The equivalent out-of-sample correlations were 0.63, 0.58 and 0.69 for stunting, wasting and underweight, respectively, at the pixel level, and 0.96, 0.95 and 0.98, respectively, at the national level. We also used various out-of-sample validation strategies to assess the fit of our models. For example, for stunting we demonstrate that our models, aggregated to the national level over five-year periods, have a small average root mean square error (0.020, ranging from 0.017 to 0.023), a small average mean error (0.0175, 0.001–0.012), a well-calibrated average 95% coverage (93.25%, ranging from 91.6% to 94.3%) and a high concordance with existing small area estimates (Supplementary Fig. 31). All model validation procedures and corresponding results are provided in the Supplementary Methods.

### Projections

To compare our estimated rates of improvement in CGF prevalence over the last 15 years with the improvements needed between 2015 and 2025 to meet the WHO GNT, we performed a simple projection using estimated AROCs applied to the final year of our estimates. A full predictive forecast was not available due to a lack of available forecasts for many of our covariates.

For each CGF indicator *i*, we calculated log-additive annual rates of change at each pixel *j*, by logit-transforming our 16 years of posterior mean prevalence estimates, 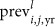, and calculating the annual rate of change between each pair of adjacent years starting with 2001:
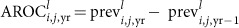
We then calculated a weighted AROC for each indicator–pixel by taking a weighted average across the years, where more recent AROCs are given more weight in the average. We defined the weights to be:
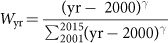
where *γ* may be chosen to give varying amounts of weight across the years. For this set of projections we selected *γ* = 1, resulting in a linear weighting scheme that has been tested and vetted for use in projecting the health-related SDGs^9^. For any indicator and for any pixel, we then calculated the average AROC to be:
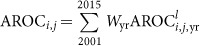
Finally, we calculated the projections by applying the ten years of the annual rates of change at each pixel in our mean 2015 mean prevalence estimates:
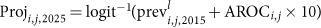
This projection scheme is analogous to the methods used in the GBD 2016 measurement of progress and projected attainment of health-related SDGs^9^. An evaluation of the projection methodology and the implicit assumptions involved can be found in the Supplementary Methods.

### Relative WHO GNT interpretation

The WHO GNT are composed of both relative (for example, 40% reduction in stunting relative to 2010) and fixed (for example, less than 5% wasting) targets. In order to compare our modelled results to the relative WHO GNT, we computed the population-weighted aggregated prevalence in 2010 from GBD 2016^1^ results across all countries for which we made estimates. We then set a fixed target for every pixel in our modelled domain to be a reduction based on the 2010 continent-level aggregated prevalences. This interpretation of the WHO GNT was used to set a fixed target across space while ensuring that locations that were already performing favourably were not characterized as being behind pace to reach the targets due to their early and continued low prevalences across time. This yielded a stunting prevalence target of 24.2%, and an underweight target prevalence of 13.5%.

### Limitations

This work should be assessed in full acknowledgement of the data and methodological limitations. While our present study is informed by 209 sources (totalling 1.29 million measured children), areas of greatest uncertainty (Figs 1f, 2f and Extended Data Fig. 2f) usually correspond to those in need of newer and/or updated information (Extended Data Figs 5, 6 and Supplementary Figs 2, 3). Expansion to additional countries and indicators underscores the need for enhanced data collection (and equally importantly, retrospective data retrieval) as we iteratively update the measurement of progress towards global targets. While not a focus of this study, a combination of the magnitude of CGF indicator prevalence (Figs 1c, 2c and Extended Data Fig. 2c), the uncertainty in its estimation (Figs 1f, 2f and Extended Data Fig. 2f), and our knowledge of national survey coverage (Extended Data Figs 5, 6 and Supplementary Figs 2, 3) can be used to help to identify countries and vulnerable sub-populations that would benefit from further survey enumeration.

There are limitations to the data used in this analysis and thus areas for future refinement. For example, the height or weight of children may have been measured or recorded incorrectly due to equipment calibration or user error, or based on difficulties originating from measuring younger children lying down rather than standing up^65^. Levels of ‘missingness’ in these survey data may also be high due to recall error of a child’s birthday. Given that growth standards are age- and sex-specific, children without detailed age information were excluded from the analysis (see Supplementary Information). In addition, a child must have been present in the home in order for the survey taker to record measurements. Given that only children alive at the time of the survey could be counted, children under 5 who died due to undernourishment or other causes before the survey was taken would not have been measured. Conflict zones in select countries or regions may also have been excluded from surveying because of security and safety issues. The direction of all of these biases is towards an underestimation of CGF.

Moreover, our estimates are not stratified by sex, wealth or any other socio-economic indicators. This may mask higher rates of CGF present in sub-populations within the areas measured and while this work presents a very fine scale for comprehensive geospatial estimates of child growth failure, the 5 × 5-km resolution is still too coarse to account for urban slums and other hyperspecific spatial disparities. Similarly, relatively coarse AROCs taken across time may obscure higher-frequency changes within the time series, and more research in studying and summarizing spatially correlated temporal trends should be pursued. Although comprehensive, due to a lack of high-resolution spatial data, our set of included covariates does not cover all CGF drivers and confounders. On the modelling side, we have attempted to propagate as much uncertainty through the various modelling stages, but there are still some propagations, such as incorporating uncertainty from the child model ensemble fits, that proved computationally infeasible. Future research is also ongoing to develop computational methods for better geostatistical integration of point and areal data to continental-scale mapping studies for a variety of indicators. These geostatistical tools are driven primarily by infrequently reported national survey data and are thus well-positioned for monitoring and evaluating progress across years, but are not suited for day-to-day assessments of CGF vulnerability. We show, however, that there is considerable room for exciting harmonization with such efforts, for example, by focusing attention of early warning efforts on populations that are the most vulnerable and least resilient^66^.

### Code availability

All code used for these analyses is publicly available online at http://ghdx.healthdata.org/record/africa-child-growth-failure-geospatial-estimates-2000-2015.

### Data availability

The findings of this study are supported by data that are available in public online repositories, data that are publicly available upon request from the data provider, and data that are not publicly available due to restrictions by the data provider, which were used under license for the current study, but may be available from the authors upon reasonable request and permission of the data provider. A detailed table of data sources and availability can be found in Supplementary Table 2.

Administrative boundaries were retrieved from the Global Administrative Unit Layers (GAUL) dataset, implemented by the FAO within the CountrySTAT and Agricultural Market Information System (AMIS) projects^44^. Land cover was retrieved from the online Data Pool, courtesy of the NASA EOSDIS Land Processes Distributed Active Archive Center (LP DAAC), USGS/Earth Resources Observation and Science (EROS) Center^45^. Lakes were retrieved from the Global Lakes and Wetlands Database (GLWD), courtesy of the World Wildlife Fund and the Center for Environmental Systems Research, University of Kassel^46^,^47^. Populations were retrieved from WorldPop^48^,^49^.

## Supplementary Material

Life Sciences Reporting Summary

Supplementary Information This file contains Supplementary Figures 1-46, Supplementary Tables 1-19, Supplementary Data, a Supplementary Discussion, Supplementary Methods and Supplementary References – see contents page for details.

## Figures and Tables

**Figure 1:**
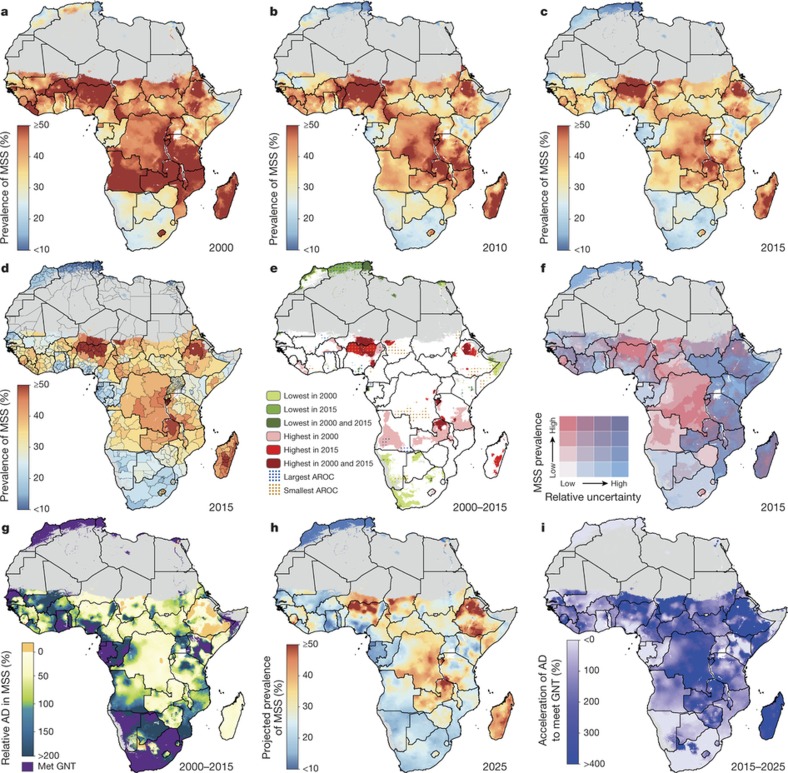
Prevalence of stunting (2000–2015) in children under five and progress towards 2025. **a**–**c**, Prevalence of moderate and severe stunting (MSS) at the 5 × 5-km resolution in 2000 (**a**), 2010 (**b**) and 2015 (**c**). **d**, Prevalence of stunting at the first administrative subdivision in 2015. **e**, Overlapping population-weighted lowest and highest 10% of pixels and AROC in stunting from 2000 to 2015 across the continent. **f**, Overlapping population-weighted quartiles of stunting and relative 95% uncertainty in 2015. **g**, Annualized decrease (AD) in stunting prevalence from 2000 to 2015 relative to rates needed during 2015–2025 to meet the WHO GNT. 100% indicates the annualized decrease from 2000 to 2015 equivalent to the pace of progress required during 2015–2025 to meet the WHO GNT by 2025 (40% decrease in stunting, relative to 2010). Blue pixels exceeded this pace; green to yellow pixels proceeded at a slower rate than required; orange pixels were non-decreasing; and purple pixels were estimated to have met the target by 2015 (‘Met GNT’). **h**, Pixel-level prevalence of stunting was predicted for 2025 on the basis of the annualized decrease achieved from 2000 to 2015 and projected from 2015. **i**, Acceleration in the annualized decrease in stunting required to meet the WHO GNT by 2025. Purple pixels were either non-decreasing or must accelerate their rate of decline by more than 400% over 2000–2015 rates during 2015–2025 to achieve the target; white pixels require no increase. Maps reflect administrative boundaries, land cover, lakes and population; pixels with fewer than ten people per 1 × 1 km and classified as ‘barren or sparsely vegetated’ are coloured in grey^44^,^45^,^46^,^47^,^48^,^49^.

**Figure 2:**
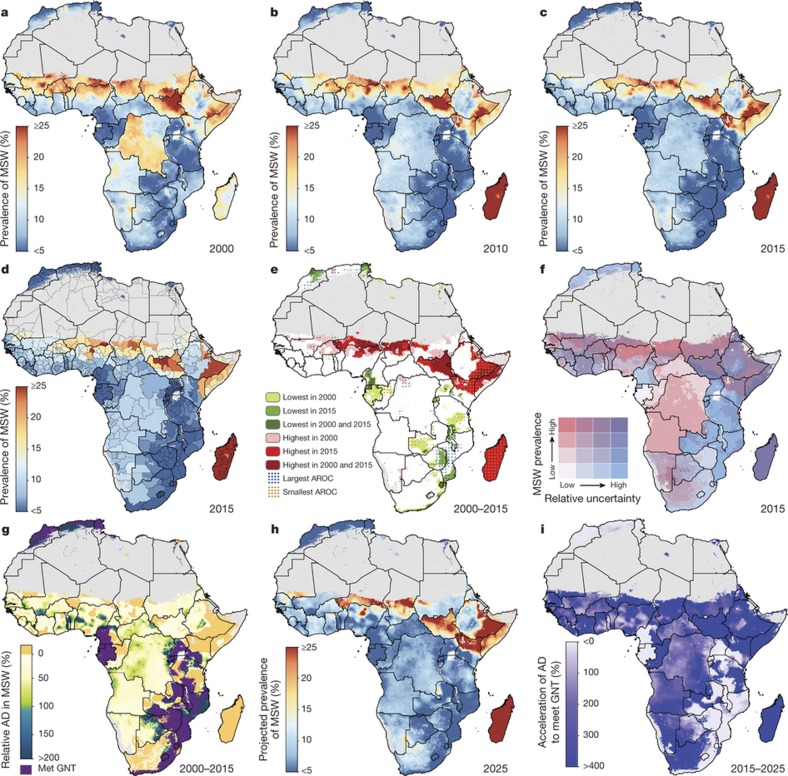
Wasting prevalence (2000–2015) in children under five and progress towards 2025. **a**–**c**, Prevalence of moderate and severe wasting (MSW) at the 5 × 5-km resolution in 2000 (**a**), 2010 (**b**) and 2015 (**c**). **d**, Prevalence of wasting at the first administrative subdivision in 2015. **e**, Overlapping population-weighted lowest and highest 10% of pixels and AROC in wasting from 2000 to 2015 across the continent. **f**, Overlapping population-weighted quartiles of wasting and relative 95% uncertainty in 2015. **g**, Annualized decrease in wasting prevalence from 2000 to 2015 relative to rates needed during 2015–2025 to meet the WHO GNT. 100% indicates the annualized decrease from 2000 to 2015 equivalent to the pace of progress required during 2015–2025 to meet the WHO GNT by 2025 (wasting less than 5%). Blue pixels exceeded this pace; green to yellow pixels proceeded at a slower rate than required; orange pixels were non-decreasing; and purple pixels were estimated to have met the target by 2015. **h**, Pixel-level prevalence of wasting was predicted for 2025 on the basis of the annualized decrease achieved from 2000 to 2015 and projected from 2015. **i**, Acceleration in annualized decrease required to meet the WHO GNT by 2025. Purple pixels were either non-decreasing or must accelerate their rate of decline by more than 400% over 2000–2015 rates during 2015–2025 to achieve the target; white pixels require no increase. Maps reflect administrative boundaries, land cover, lakes and population; pixels with fewer than ten people per 1 × 1 km and classified as ‘barren or sparsely vegetated’ are coloured in grey^44^,^45^,^46^,^47^,^48^,^49^.

**Figure 3:**
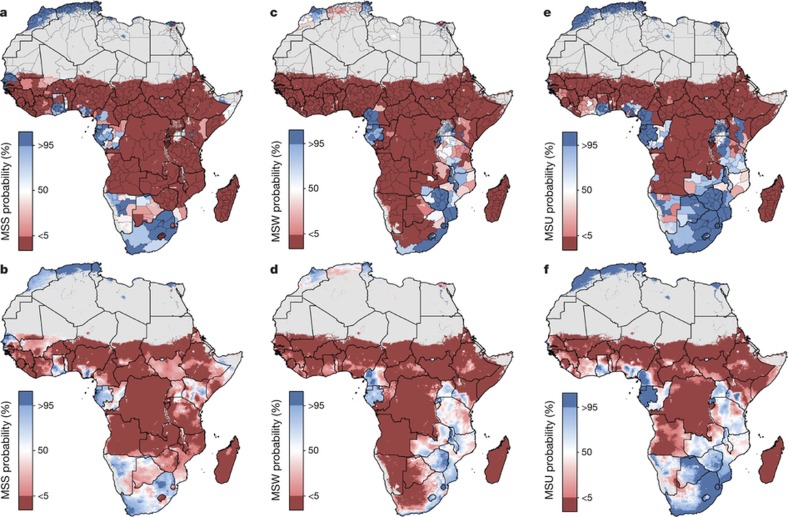
Probability that the WHO GNT has been achieved in 2015 at the first administrative subdivision and 5 × 5-km pixel level for stunting, wasting and underweight. **a**–**f**, Probability of WHO GNT achievement in 2015 at the first administrative subdivision and 5 × 5-km level for moderate and severe stunting (**a**, **b**), moderate and severe wasting (**c**, **d**) and moderate and severe underweight (**e**, **f**). The probability that dark-blue pixels have met the WHO GNT in 2015 is greater than 95%, and less than 5% for dark-red pixels. Estimates for 2015 at the 5 × 5-km level have been calculated using population-weighting based on the population of children under five and probabilities that the WHO GNT were met in 2015. Maps reflect administrative boundaries, land cover, lakes and population; pixels with fewer than ten people per 1 × 1 km and classified as ‘barren or sparsely vegetated’ are coloured in grey^44^,^45^,^46^,^47^,^48^,^49^.

**Figure 1:**
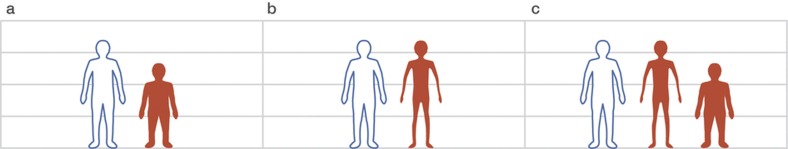
Measurement of child growth failure. **a**, Stunting is a manifestation of chronic malnutrition and is defined as a height-for-age *z* score (HAZ) that is two or more standard deviations (s.d.) below the reference median. **b**, Wasting is an emaciated state resulting from acute malnutrition and is defined a weight-for-height *z* score (WHZ) of <−2. **c**, Underweight is a weight-for-age *z* score (WAZ) of <−2 and is considered a marker of subacute malnutrition, but is nonspecific from an anthropometric standpoint, because it can indicate either low weight for height, low height for age or some combination of both. There are multiple permutations of child growth failure and the silhouettes are simply illustrative of what a stunted, wasted or underweight child may look like. The World Health Organization Global Targets 2025 to improve maternal, infant and young child nutrition call for a 40% reduction in stunting and a reduction and maintenance of child wasting to less than 5% in children under five. While there is no target for child underweight, a reduction of 40% was used in this analysis.

**Figure 2:**
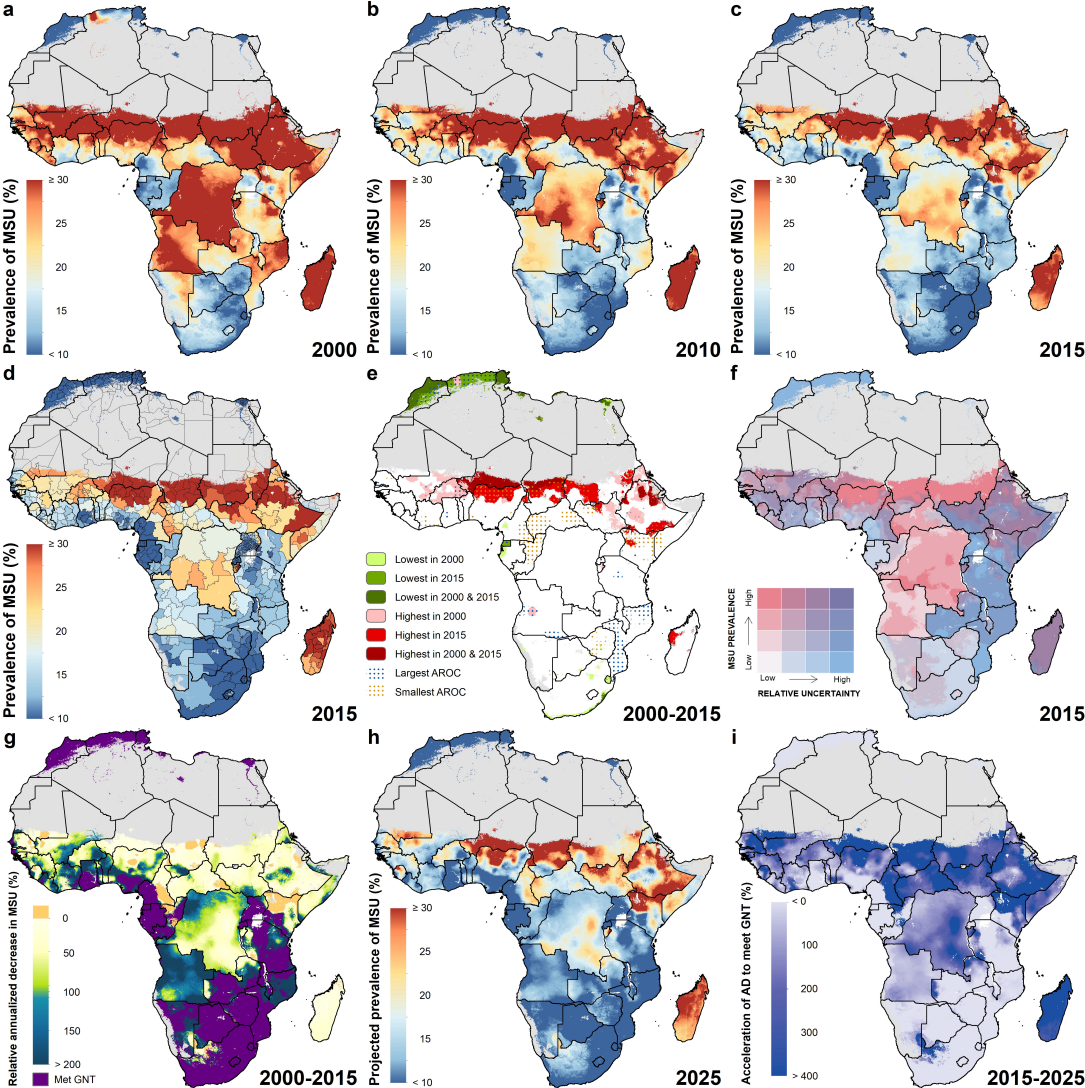
Underweight prevalence in children under five (2000–2015) and progress towards 2025. **a**–**c**, Moderate and severe underweight (MSU) prevalence at the 5 × 5-km resolution in 2000 (**a**), 2010 (**b**) and 2015 (**c**). **d**, Underweight prevalence at the first administrative subdivision in 2015. **e**, Overlapping population-weighted lowest and highest 10% of pixels and annualized rates of change in underweight from 2000 to 2015 across the continent. **f**, Overlapping population-weighted quartiles of underweight and relative 95% uncertainty in 2015. **g**, Annualized decrease in underweight prevalence from 2000 to 2015 relative to rates needed during 2015–2025 to meet the WHO GNT. 100% indicates the annualized decrease from 2000 to 2015 equivalent to the pace of progress required during 2015–2025 to meet a 40% decrease in underweight by 2025, relative to 2010. Blue pixels exceeded this pace; green to yellow pixels proceeded at a slower rate than required; orange pixels were non-decreasing; and purple pixels were estimated to have met the target by 2015. This target was internally constructed, commensurate with the target for stunting, as there is no WHO GNT for underweight. **h**, Pixel-level underweight prevalence was predicted for 2025 on the basis of the annualized decrease achieved from 2000 to 2015 and projected from 2015 estimates. **i**, Acceleration in annualized decrease required to meet the WHO GNT by 2025. Purple pixels were either non-decreasing or must accelerate their rate of decline by more than 400% over 2000–2015 rates during 2015–2025 to achieve the target; white pixels require no increase. Maps reflect administrative boundaries, land cover, lakes and population; pixels with fewer than ten people per 1 × 1 km and classified as ‘barren or sparsely vegetated’ are coloured in grey^44^,^45^,^46^,^47^,^48^,^49^.

**Figure 3:**
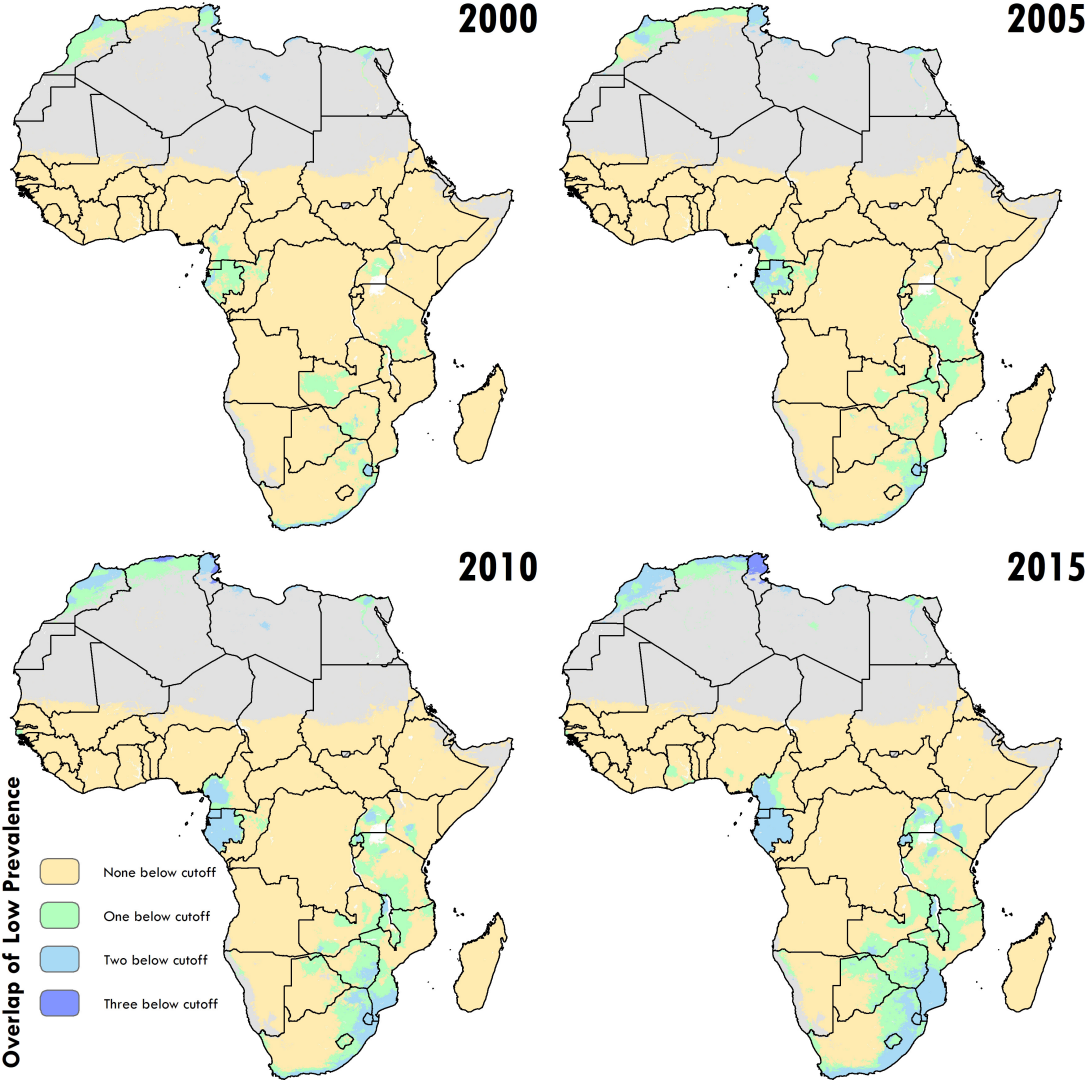
Low prevalence across stunting, wasting and underweight. Across the modelling regions and 5-year periods, these plots show locations where the prevalence of one, two or three of the indicators falls below a lower bound (10% for stunting (HAZ), 5% for wasting (WHZ) and 10% for underweight (WAZ), which correspond to the lower cut-offs used in Figs 1a, 2a, Extended Data Fig. 2a). Maps reflect administrative boundaries, land cover, lakes and population; pixels with fewer than ten people per 1 × 1 km and classified as ‘barren or sparsely vegetated’ are coloured in grey^44^,^45^,^46^,^47^,^48^,^49^.

**Figure 4:**
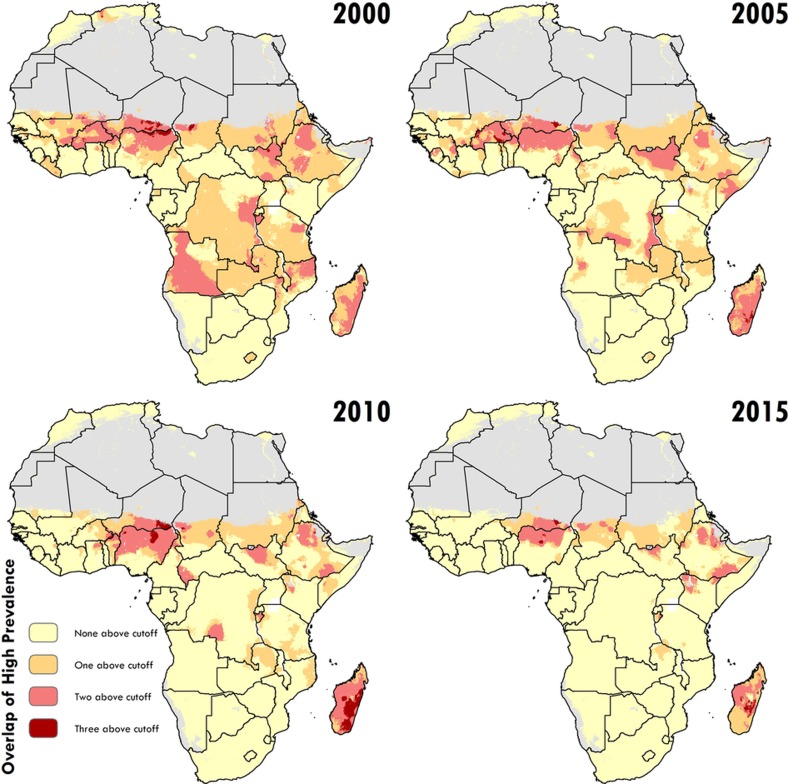
High prevalence across stunting, wasting, and underweight. Across the modelling regions and 5-year periods, these plots show locations where the prevalence of one, two or three of the indicators falls above an upper bound (50% for stunting (HAZ), 25% for wasting (WHZ) and 30% for underweight (WAZ), which correspond to the upper cut-offs used in Figs 1a, 2a, Extended Data Fig. 2a). Maps reflect administrative boundaries, land cover, lakes and population; pixels with fewer than ten people per 1 × 1 km and classified as ‘barren or sparsely vegetated’ are coloured in grey^44^,^45^,^46^,^47^,^48^,^49^.

**Figure 5:**
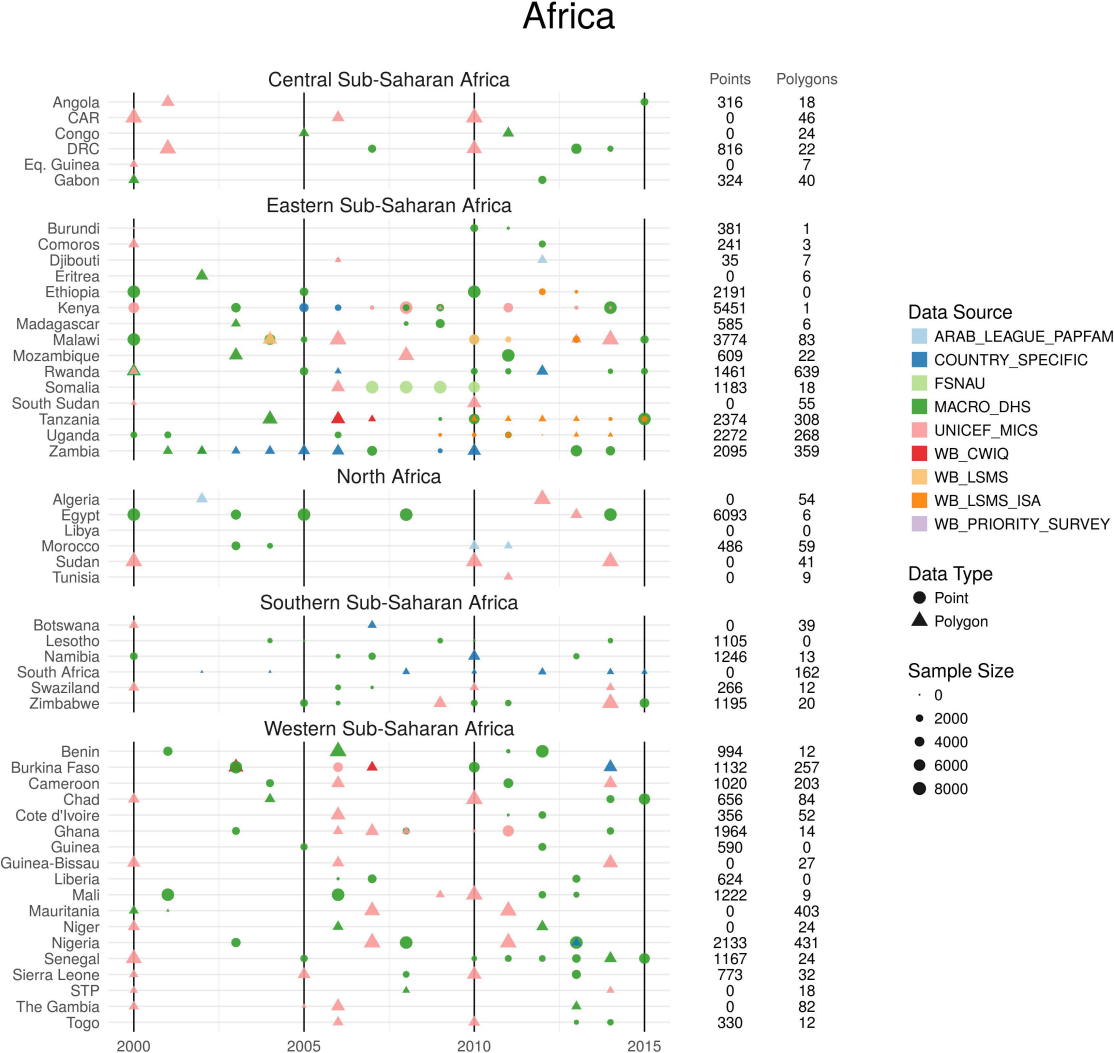
Stunting annual data availability by type and country for 2000–2015. All data are shown by country and year of survey. The total number of points and polygons (areal) for each country are plotted by data source, type and sample size. Sample size represents the number of individual microdata records for each survey. This database consists of 50,142 clusters and 4,253 polygons with a sample size totalling over 1.15 million children in Africa.

**Figure 6:**
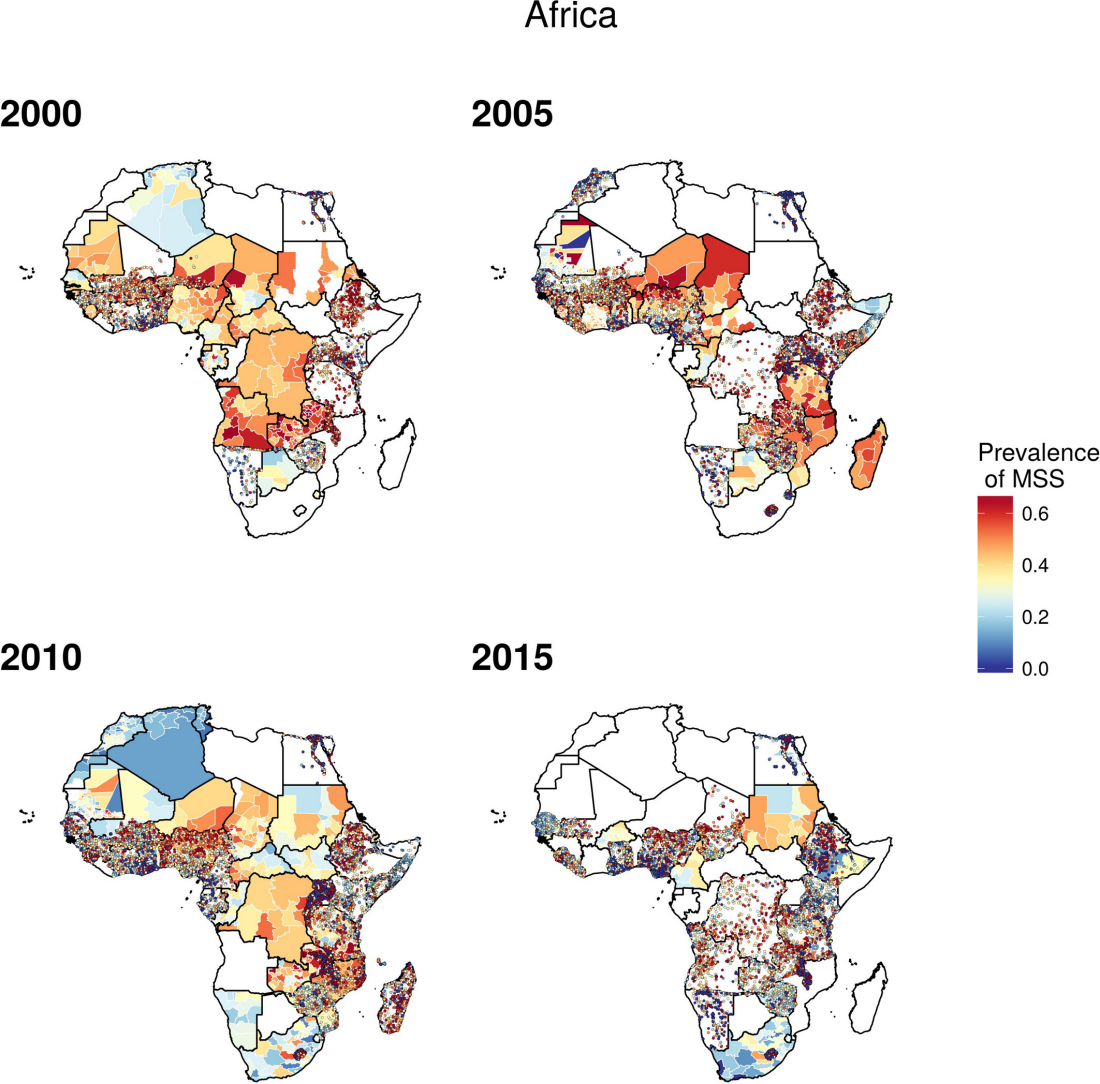
Stunting data availability map for 2000–2015. All data are shown by country and year and mapped at their corresponding geopositioned coordinate or area. Mean stunting prevalence of the input coordinate or area is mapped. This database consists of 50,142 clusters and 4,253 polygons with a sample size totalling over 1.15 million children in Africa. Maps reflect administrative boundaries, land cover, lakes and population; pixels with fewer than ten people per 1 × 1 km and classified as ‘barren or sparsely vegetated’ are coloured in grey^44^,^45^,^46^,^47^,^48^,^49^.

**Figure 7:**
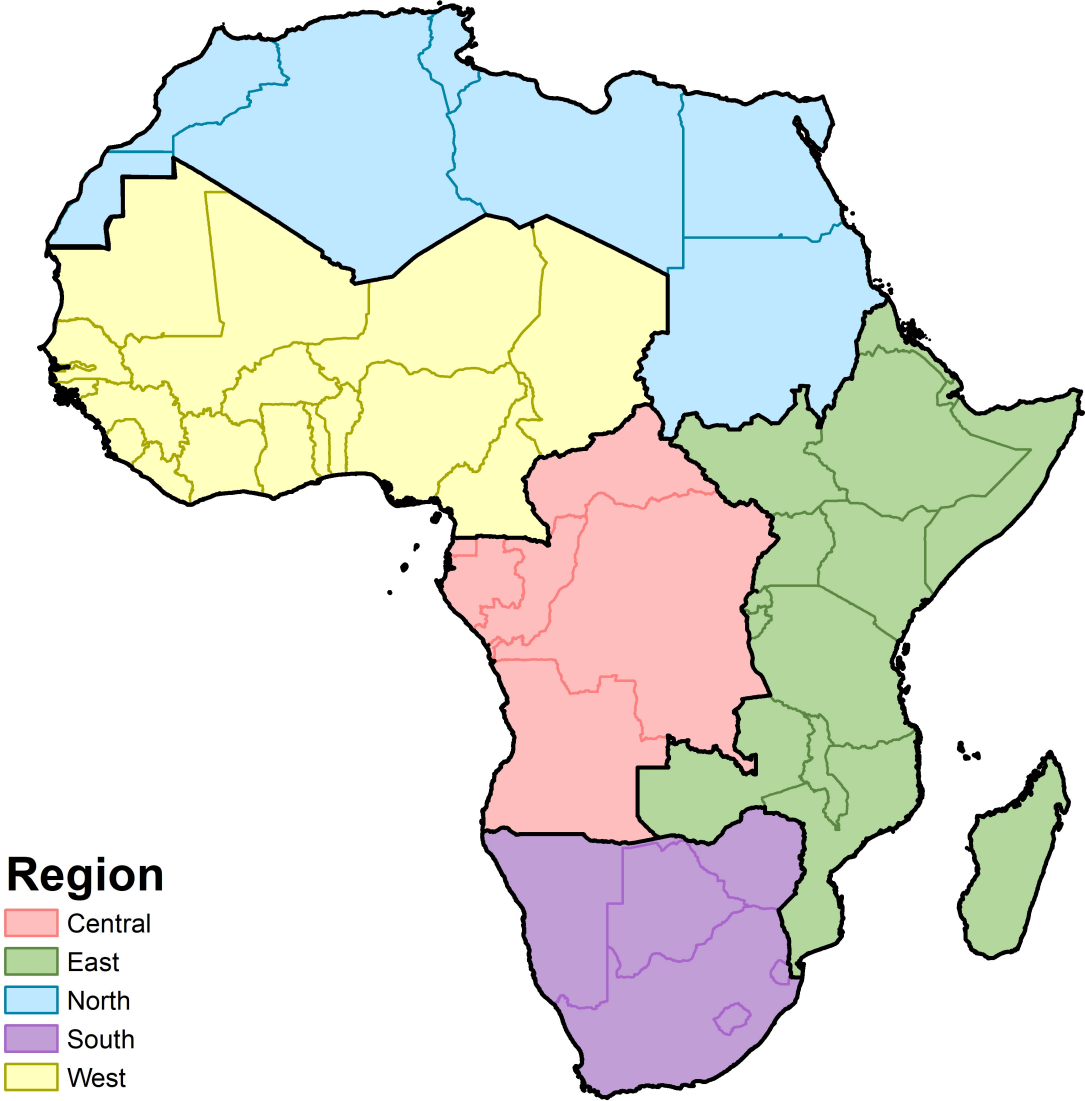
Map of GBD regions. Modelling regions were defined as the five GBD regions of Central (central SSA), East (eastern SSA), North (North Africa and the Middle East), South (southern SSA) and West Africa (western SSA)^57^. As this study was limited to mainland Africa and African island nations, select countries were excluded from the North Africa and Middle East region (Afghanistan, Bahrain, Iran, Iraq, Jordan, Kuwait, Lebanon, Oman, Palestinian territories, Qatar, Saudi Arabia, Syria, Turkey, United Arab Emirates, and Yemen). Western Sahara was included as part of the North region.
