# Supplementary material for: Mapping child growth failure in Africa between 2000 and 2015
Source: Nature. 2018 Mar 1;555(7694):41–7. doi: 10.1038/nature25760 (PMC6346257; doi:10.1038/nature25760)
Supplement: Supplementary Information — This file contains Supplementary Figures 1-46, Supplementary Tables 1-19, Supplementary Data, a Supplementary Discussion, Supplementary Methods and Supplementary References – see contents page for details. [file nature25760-s2.pdf]

doi:10.1038/nature25760

Table of Contents

Supplementary Figures 2
Supplementary Tables 4
1.0 GATHER compliance 5
2.0 Supplementary discussion 7
2.1 Remedial actions needed to reduce child growth failure 7
3.0 Supplementary data 9
3.1 Data excluded from model 15
4.0 Supplementary covariates 20
5.0 Supplementary methods 29
5.1 Seasonality adjustment 29
5.2 Cluster combination and spatial integration over polygon records 31
5.3 Geostatistical model 33
5.3.1 Model geographies 33
5.3.2 Ensemble covariate modelling 33
5.3.3 Model description 39
5.3.4 Priors 40
5.3.5 Mesh construction 41
5.3.6 Model fitting and estimate generation 41
5.3.7 Model Results 44
5.4 Model validation 51
5.4.1 In-sample metrics 51
5.4.2 Metrics of predictive validity 51
5.4.3 Model comparison 52
5.4.4 Stunting validation metrics 56
5.4.5 Wasting validation metrics 63
5.4.6 Underweight validation metrics 70
5.4.7 Absolute error plots 77
5.4.8 Post estimation calibration to national estimates 80
5.4.9 Verification and comparison against other subnational CGF estimates 84
5.4.10 Evaluation of projection methodology 85
6.0 Supplementary references 88

Supplementary Figures

Supplementary Figure 1. Stunting data availability by type and country, 2000–2015. 17
Supplementary Figure 2. Wasting data availability by type and country, 2000–2015. 18
Supplementary Figure 3. Underweight data availability by type and country, 2000–2015. 19
Supplementary Figure 4. Covariates. 28
Supplementary Figure 5. Periodic seasonality adjustment. 29
Supplementary Figure 6. Cluster and polygon-level seasonality wasting adjustments. 30
Supplementary Figure 7. Polygon resampling. 32
Supplementary Figure 8. Stunting covariate extrapolation. 35
Supplementary Figure 9. Wasting covariate extrapolation. 36
Supplementary Figure 10. Underweight covariate extrapolation. 37
Supplementary Figure 11. Ensemble predicted rasters. 38
Supplementary Figure 12. Finite elements mesh. 43
Supplementary Figure 13. Stunting posterior means and 95% uncertainty intervals. 48
Supplementary Figure 14. Wasting posterior means and 95% uncertainty intervals. 49
Supplementary Figure 15. Underweight posterior means and 95% uncertainty intervals. 50
Supplementary Figure 16. Stunting out-of-sample model comparisons. 53
Supplementary Figure 17. Wasting out-of-sample model comparisons. 54
Supplementary Figure 18. Underweight out-of-sample model comparisons. 55
Supplementary Figure 19. Stunting admin 0 aggregation in-sample. 57
Supplementary Figure 20. Stunting admin 0 aggregation out-of-sample. 58
Supplementary Figure 21. Stunting admin 1 aggregation in-sample. 59
Supplementary Figure 22. Stunting admin 1 aggregation out-of-sample. 60
Supplementary Figure 23. Stunting holdout units aggregation in-sample. 61
Supplementary Figure 24. Stunting holdout units aggregation out-of-sample. 62
Supplementary Figure 25. Wasting admin 0 aggregation in-sample. 64
Supplementary Figure 26. Wasting admin 0 aggregation out-of-sample. 65
Supplementary Figure 27. Wasting admin 1 aggregation in-sample. 66
Supplementary Figure 28. Wasting admin 1 aggregation out-of-sample. 67
Supplementary Figure 29. Wasting holdout units aggregation in-sample. 68
Supplementary Figure 30. Wasting holdout units aggregation out-of-sample. 69
Supplementary Figure 31. Underweight admin 0 aggregation in-sample. 71
Supplementary Figure 32. Underweight admin 0 aggregation out-of-sample. 72
Supplementary Figure 33. Underweight admin 1 aggregation in-sample. 73
Supplementary Figure 34. Underweight admin 1 aggregation out-of-sample. 74
Supplementary Figure 35. Underweight holdout units aggregation in-sample. 75
Supplementary Figure 36. Underweight holdout units aggregation out-of-sample. 76
Supplementary Figure 37. Plots of stunting bias in Africa out-of-sample. 77
Supplementary Figure 38. Plots of wasting bias in Africa out-of-sample. 78
Supplementary Figure 39. Plots of underweight bias in Africa out-of-sample. 79
Supplementary Figure 40. Comparison of aggregated stunting MBG estimates to GBD 2016 stunting estimates. 81

Supplementary Figure 41. Comparison of aggregated wasting MBG estimates to GBD 2016 stunting estimates..82
Supplementary Figure 42. Comparison of aggregated underweight MBG estimates to GBD 2016 stunting estimates..83
Supplementary Figure 43. Comparison of MBG estimates aggregated to admin 1 to DHS admin 1 estimates..84
Supplementary Figure 44. Stunting projection comparison. 85
Supplementary Figure 45. Wasting projection comparison...86
Supplementary Figure 46. Underweight projection comparison. 87

doi:10.1038/nature25760

Supplementary Tables

Supplementary Table 1. Guidelines for Accurate and Transparent Health Estimates Reporting (GATHER) checklist 5
Supplementary Table 2. Household surveys used in mapping. 9
Supplementary Table 3. Covariates used in mapping.....20
Supplementary Table 4. Spatial hyperparameter priors by region 41
Supplementary Table 5. Stunting fitted parameters.....45
Supplementary Table 6. Wasting fitted parameters.....46
Supplementary Table 7. Underweight fitted parameters. 47
Supplementary Table 8. Predictive metrics for stunting aggregated to admin 0.....56
Supplementary Table 9. Predictive metrics for stunting aggregated to admin 1.....56
Supplementary Table 10. Predictive metrics for stunting aggregated to admin 2.....56
Supplementary Table 11. Predictive metrics for stunting aggregated to holdout units.....56
Supplementary Table 12. Predictive metrics for wasting aggregated to admin 0. 63
Supplementary Table 13. Predictive metrics for wasting aggregated to admin 1. 63
Supplementary Table 14. Predictive metrics for wasting aggregated to admin 2. 63
Supplementary Table 15. Predictive metrics for wasting aggregated to holdout units. 63
Supplementary Table 16. Predictive metrics for underweight aggregated to admin 0.....70
Supplementary Table 17. Predictive metrics for underweight aggregated to admin 1 70
Supplementary Table 18. Predictive metrics for underweight aggregated to admin 2 70
Supplementary Table 19. Predictive metrics for underweight aggregated to holdout units.....70

1.0 GATHER compliance

Supplementary Table 1. Guidelines for Accurate and Transparent Health Estimates Reporting (GATHER) checklist

Item #	Checklist item	Reported on page #
**Objectives and funding**		
1	Define the indicator(s), populations (including age, sex, and geographic entities), and time period(s) for which estimates were made.	Main text: Introduction, Methods (Data)
2	List the funding sources for the work.	Main text: Acknowledgements
**Data Inputs**		
For all data inputs from multiple sources that are synthesized as part of the study:		
3	Describe how the data were identified and how the data were accessed.	Main text: Methods (Data, Data availability), Supplementary Information: 3.0 Supplementary data
4	Specify the inclusion and exclusion criteria. Identify all ad-hoc exclusions.	Main text: Methods (Data exclusion criteria), Supplementary Information: 3.1 Data excluded from model
5	Provide information on all included data sources and their main characteristics. For each data source used, report reference information or contact name/institution, population represented, data collection method, year(s) of data collection, sex and age range, diagnostic criteria or measurement method, and sample size, as relevant.	Supplementary Information: 3.0 Supplementary data
6	Identify and describe any categories of input data that have potentially important biases (e.g., based on characteristics listed in item 5).	Main text: Methods (Limitations)
For data inputs that contribute to the analysis but were not synthesized as part of the study:		
7	Describe and give sources for any other data inputs.	Main text: Methods (Data: Spatial covariates), Supplementary Information: 4.0 Supplementary covariates
For all data inputs:		
8	Provide all data inputs in a file format from which data can be efficiently extracted (e.g., a spreadsheet rather than a PDF), including all relevant meta-data listed in item 5. For any data inputs that cannot be shared because of ethical or legal reasons, such as third-party ownership, provide a contact name or the name of the institution that retains the right to the data.	Available at http://ghdx.healthdata.org/record/africa-child-growth-failure-geospatial-estimates-2000-2015 Supplementary Information: 3.0 Supplementary data

doi:10.1038/nature25760

**Data analysis**		
**9**	Provide a conceptual overview of the data analysis method. A diagram may be helpful.	Main text: Methods (Data), Supplementary Information: 5.1 Seasonality adjustment, 5.2 Cluster combination and spatial integration over polygon records
**10**	Provide a detailed description of all steps of the analysis, including mathematical formulae. This description should cover, as relevant, data cleaning, data pre-processing, data adjustments and weighting of data sources, and mathematical or statistical model(s).	Main text: Methods (Analysis), Supplementary Information: 5.3 Geostatistical model
**11**	Describe how candidate models were evaluated and how the final model(s) were selected.	Main text: Methods (Analysis: Model validation), Supplementary Information: 5.4 Model validation
**12**	Provide the results of an evaluation of model performance, if done, as well as the results of any relevant sensitivity analysis.	Main text: Methods (Analysis), Supplementary Information: 5.4 Model validation
**13**	Describe methods for calculating uncertainty of the estimates. State which sources of uncertainty were, and were not, accounted for in the uncertainty analysis.	Main text: Methods (Analysis: Geostatistical model), Supplementary Information: 5.3.3 Model description, 5.3.6 Model fitting and estimate generation
**14**	State how analytic or statistical source code used to generate estimates can be accessed.	Available at http://ghdx.healthdata.org/record/africa-child-growth-failure-geospatial-estimates-2000-2015
**Results and Discussion**		
**15**	Provide published estimates in a file format from which data can be efficiently extracted.	Raster files for spatial data and CSVs of estimates available at http://ghdx.healthdata.org/record/africa-child-growth-failure-geospatial-estimates-2000-2015
**16**	Report a quantitative measure of the uncertainty of the estimates (e.g. uncertainty intervals).	Main text: Figs 1f, 2f, Extended Data Fig 2f, Supplementary Information: Supplementary Figs 13-15
**17**	Interpret results in light of existing evidence. If updating a previous set of estimates, describe the reasons for changes in estimates.	Main text: Disparate progress in reducing child growth failure, Outlook for the 2025 GNT
**18**	Discuss limitations of the estimates. Include a discussion of any modelling assumptions or data limitations that affect interpretation of the estimates.	Main text: Methods (Limitations)

doi:10.1038/nature25760

2.0 Supplementary discussion

2.1 Remedial actions needed to reduce child growth failure

Effectively reducing child growth failure (CGF) requires delivery of proven prevention and intervention packages, including health services, nutrition, and education programmes for pregnant women and parents, treatment programmes for acute malnutrition, and targeted policies to stabilise food supplies, improve societal conditions predisposing to CGF, and maximise the efficiency with which appropriate help is delivered to the specific people in the specific communities that need it most.

The "first 1000 days" from conception to age 2 are a crucial development window[1], so first-line efforts in the locations with the highest rates of CGF should focus on prevention strategies in mothers and babies. Maternal problems predisposing to CGF include antenatal infections, inadequate birth spacing, maternal calorie and micronutrient deficiencies, poor education, and strenuous physical work. Infant- and toddler-focused interventions should aim both to prevent infections whose metabolic demands can quickly lead infants to have inadequate energy supply[2], and optimise nutritional intake, including breastfeeding, intermittent micronutrient supplementation, and complementary feeding when appropriate or needed.[3]

WHO guidelines recommend inpatient care for treatment of wasting (severe acute malnutrition [SAM] and moderate acute malnutrition [MAM]) at referral centers.[4,5] In many settings, likely including many of those identified clearly in these maps as having high burden and/or little improvement, a shortage of trained, adequately supported health providers can be a limiting factor in an inpatient-only approach. In such settings, an outpatient-oriented community management of acute malnutrition (CMAM) model is likely to have a more significant impact. By encouraging early identification of MAM and SAM, enhanced parental compliance, and provision of ready-to-use therapeutic foods (RUTF),[6,7] the CMAM model has been shown to be effective in inducing nutrition recovery. Precision public health mapping can help guide investment priorities for establishing and/or strengthening such programmes by examining the geographic distribution of CGF within relevant administrative units. Strategically placing CMAM programmes in high-burden rural settings may be appropriate, while inpatient programmes may be more successful – and have bigger impact – if they are situated in nearby population centers so as to ensure appropriate infrastructure, human resources, capital, and logistical support.

Governmental policies should focus on promoting sustainable agriculture and land management, universal food fortification, and preparedness for climactic and seasonal fluctuations in domestic crop yields.[2,8] Because of the close links between CGF and sociodemographic deprivation, society-wide efforts to improve education, reduce poverty, and ensure an equal status for women are likely to positively impact health outcomes.[9] Economic and financial policies, including engagement with stakeholders from private industry when possible, should aim to encourage widespread availability and consumption of nutritious foods.[10] All policy implementation efforts should be cognizant to employ optimal delivery platforms and ensure appropriate matching of interventions to local epidemiologic profiles. For example, while perinatal care and CMAM programmes may be naturally best centered in medical facilities, other distribution hubs like

markets, community centres, religious centres, or schools may be more appropriate for delivery of nutrition education programmes, fortified foods and supplements, or infection prevention programmes.[3] All of these strategies are better focused – and synergised – with the subnational insights afforded by precision public health mapping.

doi:10.1038/nature25760

3.0 Supplementary data

The data sources used to model CGF indicators are described below. Information on survey locations, years, source, and number of individuals, polygons, and/or geo-positioned clusters can be found in Supplementary Table 2. Supplementary Figures 1, 2, and 3 display data availability for each indicator. Data from 1998 and 1999 were mapped to 2000 due to both data scarcity in earlier years and to help establish a solid baseline. Reasons for datasets being excluded from analysis are detailed in Supplementary Information 3.1.

Supplementary Table 2. Household surveys used in mapping.
Number identification (NID) can be used to locate a particular data source in the Global Health Data Exchange at http://ghdx.healthdata.org/.

Country	Survey year(s)	Source	Number Identification (NID)	Number of individuals	Number of geo-positioned clusters	Number of polygons (areal)
Algeria	2002-2003	PAPFAM	627	4,728	0	47
Algeria	2012-2013	UNICEF MICS	210614	13,825	0	7
Angola	2001	UNICEF MICS	687	5,370	0	18
Angola	2015-2016	DHS Program	218555	6,487	625	0
Benin	2001	DHS Program	18950	4,509	247	0
Benin	2006	DHS Program	18959	13,361	0	12
Benin	2011-2012	DHS Program	79839	11,312	745	0
Botswana	2000	UNICEF MICS	1404	2,846	0	14
Botswana	2007-2008	Family Health Survey	22125	2,631	0	24
Burkina Faso	1998	World Bank Priority Survey	1912	3,676	0	33
Burkina Faso	1998-1999	DHS Program	19076	4,696	208	0
Burkina Faso	2003	CWIQ	1855	7,432	0	232
Burkina Faso	2003	DHS Program	19088	8,645	397	0
Burkina Faso	2006	UNICEF MICS	1927	4,947	195	0
Burkina Faso	2007	CWIQ	18499	4,077	0	13
Burkina Faso	2010-2011	DHS Program	19133	6,327	540	0
Burkina Faso	2014	Continuous Multisectoral Survey	236156	1,075	0	13
Burundi	2000	UNICEF MICS	1994	63	0	17
Burundi	2010-2011	DHS Program	30431	3,497	376	0
Cameroon	1998	DHS Program	19198	1,926	0	10
Cameroon	2004	DHS Program	19211	3,325	444	0
Cameroon	2006	UNICEF MICS	2063	6,114	0	191
Cameroon	2011	DHS Program	19274	5,179	574	0
Cameroon	2014	UNICEF MICS	244455	6,768	0	12

Country	Survey year(s)	Source	Number Identification (NID)	Number of individuals	Number of geo-positioned clusters	Number of polygons (areal)
Central African Republic	2000	UNICEF MICS	2209	13,399	0	17
Central African Republic	2006	UNICEF MICS	2223	6,910	0	12
Central African Republic	2010-2011	UNICEF MICS	82832	10,267	0	17
Chad	2000	UNICEF MICS	2244	5,253	0	15
Chad	2004	DHS Program	19315	4,627	0	9
Chad	2010	UNICEF MICS	76701	15,443	0	60
Chad	2014-2015	DHS Program	157025	10,422	623	0
Comoros	2000	UNICEF MICS	3114	4,267	0	3
Comoros	2012-2013	DHS Program	76850	2,601	241	0
Côte d'Ivoire	1998-1999	DHS Program	18531	1,599	140	0
Côte d'Ivoire	2006	UNICEF MICS	26433	8,550	0	52
Côte d'Ivoire	2011-2012	DHS Program	18533	3,192	341	0
Democratic Republic of Congo	2001	UNICEF MICS	3161	9,888	0	11
Democratic Republic of Congo	2007	DHS Program	19381	3,595	293	0
Democratic Republic of Congo	2010	UNICEF MICS	26998	10,859	0	11
Democratic Republic of Congo	2013	DHS Program	76878	7,711	492	0
Djibouti	2006	UNICEF MICS	3404	2,126	35	1
Djibouti	2012	PAPFAM	218035	3,464	0	6
Egypt	2000	DHS Program	19511	10,683	985	0
Egypt	2003	DHS Program	19529	5,401	876	0
Egypt	2005	DHS Program	19521	12,547	1287	0
Egypt	2008	DHS Program	26842	10,252	1221	0
Egypt	2013-2014	UNICEF MICS	159617	5,030	0	6
Egypt	2014	DHS Program	154897	15,045	1734	0
Equatorial Guinea	2000	UNICEF MICS	3655	2,364	0	7
Eritrea	2002	DHS Program	19539	5,660	0	6
Ethiopia	2000	DHS Program	19571	8,980	533	0
Ethiopia	2005	DHS Program	19557	4,151	520	0
Ethiopia	2010-2011	DHS Program	21301	9,594	571	0
Ethiopia	2011-2012	LSMS	93848	2,438	331	0

Country	Survey year(s)	Source	Number Identification (NID)	Number of individuals	Number of geo-positioned clusters	Number of polygons (areal)
Ethiopia	2013-2014	LSMS	235215	606	280	0
Ethiopia	2016	DHS Program	218568	9,106	623	4
Gabon	2000-2001	DHS Program	19579	3,564	0	40
Gabon	2012	DHS Program	76706	3,475	324	0
Gambia	2000	UNICEF MICS	3922	3,390	0	8
Gambia	2005-2006	UNICEF MICS	3935	6,414	0	37
Gambia	2013	DHS Program	77384	3,354	0	37
Ghana	1998-1999	DHS Program	19614	2,859	396	0
Ghana	2003	DHS Program	19627	3,237	407	0
Ghana	2006	UNICEF MICS	4694	3,390	0	10
Ghana	2007-2008	UNICEF MICS	160576	8,352	0	4
Ghana	2008	DHS Program	21188	2,502	400	0
Ghana	2010-2011	UNICEF MICS	56241	435	5	0
Ghana	2011	UNICEF MICS	63993	6,854	738	0
Ghana	2014	DHS Program	157027	2,735	414	0
Guinea	1999	DHS Program	19670	4,599	292	0
Guinea	2005	DHS Program	19683	2,694	290	0
Guinea	2012	DHS Program	69761	3,208	300	0
Guinea-Bissau	2000	UNICEF MICS	4808	5,612	0	9
Guinea-Bissau	2006	UNICEF MICS	4818	5,485	0	9
Guinea-Bissau	2014	UNICEF MICS	174049	7,526	0	9
Kenya	1998	DHS Program	20132	3,121	521	0
Kenya	2000	UNICEF MICS	7387	6,675	801	0
Kenya	2003	DHS Program	20145	4,916	397	0
Kenya	2005-2006	Kenya Integrated Household Budget Survey	7375	6,971	1,283	0
Kenya	2007	UNICEF MICS	155335	917	76	0
Kenya	2008	UNICEF MICS	7401	12,222	590	0
Kenya	2008-2009	DHS Program	21365	5,335	397	0
Kenya	2009	UNICEF MICS	56420	445	0	1
Kenya	2011	UNICEF MICS	135416	4,819	289	0
Kenya	2013-2014	UNICEF MICS	203664	1,026	50	0
Kenya	2014	DHS Program	157057	18,934	1583	0
Lesotho	2004-2005	DHS Program	20167	1,385	353	0
Lesotho	2009-2010	DHS Program	21382	1,642	383	0
Lesotho	2014	DHS Program	157058	1,349	369	0
Liberia	2006-2007	DHS Program	20191	4,471	290	0
Liberia	2013	DHS Program	77385	3,263	322	0
Madagascar	2003-2004	DHS Program	20223	4,703	0	6
Madagascar	2008-2009	DHS Program	21409	5,154	583	0

doi:10.1038/nature25760

Country	Survey year(s)	Source	Number Identification (NID)	Number of individuals	Number of geo-positioned clusters	Number of polygons (areal)
Malawi	2000	DHS Program	20252	9,768	559	0
Malawi	2004-2005	DHS Program	20263	8,875	520	0
Malawi	2004-2005	LSMS	46317	6,777	0	26
Malawi	2006	UNICEF MICS	7919	22,398	0	26
Malawi	2010	DHS Program	21393	4,784	812	0
Malawi	2010-2011	LSMS	93806	7,704	768	0
Malawi	2013	LSMS	224223	2,477	543	0
Malawi	2013-2014	UNICEF MICS	161662	18,525	0	31
Malawi	2015-2016	DHS Program	218581	5,235	850	0
Mali	2001	DHS Program	20315	9,752	395	0
Mali	2006	DHS Program	20274	11,504	404	0
Mali	2009-2010	UNICEF MICS	270627	22,972	0	9
Mali	2012-2013	DHS Program	77388	4,609	412	0
Mauritania	2000-2001	DHS Program	20322	4,006	0	13
Mauritania	2007	UNICEF MICS	8115	8,210	0	170
Mauritania	2011	UNICEF MICS	152783	8,887	0	194
Morocco	2003-2004	DHS Program	20361	5,666	479	0
Morocco	2010-2011	PAPFAM	126909	6,359	0	74
Mozambique	2003	DHS Program	20394	8,279	0	11
Mozambique	2008-2009	UNICEF MICS	27031	10,849	0	11
Mozambique	2011	DHS Program	55975	9,708	609	0
Namibia	2000	DHS Program	20417	3,034	256	0
Namibia	2006-2007	DHS Program	20428	3,758	484	0
Namibia	2009-2010	Household Income and Expenditure Survey	134371	5,573	0	13
Namibia	2013	DHS Program	150382	1,843	502	0
Niger	1998	DHS Program	20537	4,037	268	0
Niger	2000	UNICEF MICS	9439	4,863	0	8
Niger	2006	DHS Program	20499	3,862	0	8
Niger	2012	DHS Program	74393	5,119	0	8
Nigeria	1999	UNICEF MICS	9506	10,596	0	31
Nigeria	2003	DHS Program	20567	4,723	359	0
Nigeria	2007	UNICEF MICS	9516	15,939	0	37
Nigeria	2008	DHS Program	21433	22,768	886	0
Nigeria	2010	DHS Program	30991	2,062	207	0
Nigeria	2011	UNICEF MICS	76703	24,256	0	37
Nigeria	2012-2013	General Household Survey	151797	2,709	0	61
Nigeria	2013	DHS Program	77390	26,427	888	0
Nigeria	2015-2016	General Household Survey	274160	2,862	512	0

doi:10.1038/nature25760

Country	Survey year(s)	Source	Number Identification (NID)	Number of individuals	Number of geo-positioned clusters	Number of polygons (areal)
Republic of Congo	2005	DHS Program	19391	4,080	0	12
Republic of Congo	2011-2012	DHS Program	56151	4,534	0	12
Rwanda	2000	DHS Program	20722	6,317	0	12
Rwanda	2000	UNICEF MICS	26930	2,815	0	12
Rwanda	2005	DHS Program	20740	3,738	455	0
Rwanda	2006	Rwanda Comprehensive Food Security/ Vulnerability Assessment and Nutrition Survey	58185	1,405	0	225
Rwanda	2010-2011	DHS Program	56040	4,120	492	0
Rwanda	2012	Rwanda Comprehensive Food Security/ Vulnerability Assessment and Nutrition Survey	151436	4,424	0	406
Rwanda	2014-2015	DHS Program	157063	3,601	491	0
São Tomé & Príncipe	2000	UNICEF MICS	27055	1,750	0	4
São Tomé & Príncipe	2008-2009	DHS Program	26866	1,644	0	7
São Tomé & Príncipe	2014	UNICEF MICS	214640	1,952	0	7
Senegal	2000	UNICEF MICS	27044	7,037	0	10
Senegal	2005	DHS Program	26855	2,846	360	0
Senegal	2010-2011	DHS Program	56063	3,814	385	0
Senegal	2012-2013	DHS Program	111432	6,014	200	0
Senegal	2014	DHS Program	191270	6,040	0	14
Senegal	2015	DHS Program	218592	6,177	214	0
Senegal	2016	DHS Program	286772	6,032	214	0
Sierra Leone	2000	UNICEF MICS	11639	2,401	0	4
Sierra Leone	2005	UNICEF MICS	11649	5,080	0	14
Sierra Leone	2008	DHS Program	21258	2,239	340	0
Sierra Leone	2010	UNICEF MICS	76700	8,267	0	14
Sierra Leone	2013	DHS Program	131467	4,654	433	0
Somalia	2006	UNICEF MICS	11774	5,776	0	18
Somalia	2007-2010	KEMRI Wellcome	270669	49,362	911	0

Country	Survey year(s)	Source	Number Identification (NID)	Number of individuals	Number of geo-positioned clusters	Number of polygons (areal)
		Trust Research Programme				
South Africa	2002	Agincourt Integrated Family Survey	135825	195	0	1
South Africa	2004	Agincourt Integrated Family Survey	135826	300	0	1
South Africa	2008	National Income Dynamics Study	27885	2,028	0	47
South Africa	2010-2011	National Income Dynamics Study	133731	1,606	0	9
South Africa	2012	National Income Dynamics Study	133732	3,156	0	60
South Africa	2014-2015	National Income Dynamics Study	265153	3,858	0	57
South Sudan	2000	UNICEF MICS	12232	1,068	0	3
South Sudan	2010	UNICEF MICS	32189	6,564	0	10
Sudan	2000	UNICEF MICS	12243	19,272	0	8
Sudan	2010	UNICEF MICS	153643	12,150	0	15
Sudan	2014	UNICEF MICS	200617	12,795	0	18
Swaziland	2000	UNICEF MICS	12320	3,295	0	4
Swaziland	2006-2007	DHS Program	20829	2,065	268	0
Swaziland	2010	UNICEF MICS	30325	2,572	0	4
Swaziland	2014	UNICEF MICS	200707	2,654	0	4
Tanzania	1999	DHS Program	20865	2,542	173	0
Tanzania	2004	LSMS	14341	1,950	885	0
Tanzania	2004-2005	DHS Program	20875	7,296	0	26
Tanzania	2006-2007	CWIQ	31831	9,948	0	28
Tanzania	2009-2010	DHS Program	21331	6,722	458	0
Tanzania	2010-2011	LSMS	81005	2,733	0	127
Tanzania	2012-2013	LSMS	224096	3,345	0	139
Tanzania	2014-2016	LSMS	311265	2,456	421	0
Tanzania	2015-2016	DHS Program	218593	9,048	607	0
Togo	1998	DHS Program	20909	3,768	282	0
Togo	2006	UNICEF MICS	12896	3,974	0	6
Togo	2010	UNICEF MICS	40021	4,680	0	6

Country	Survey year(s)	Source	Number Identification (NID)	Number of individuals	Number of geo-positioned clusters	Number of polygons (areal)
Togo	2013	DHS Program	77515	3,232	328	0
Tunisia	2011-2012	UNICEF MICS	76709	2,742	0	9
Uganda	2000-2001	DHS Program	20993	4,690	266	0
Uganda	2006	DHS Program	21014	2,221	333	0
Uganda	2009-2010	LSMS	81004	1,418	287	7
Uganda	2010-2011	LSMS	142934	1,602	408	0
Uganda	2011	DHS Program	56021	2,086	391	0
Uganda	2011-2012	LSMS	142935	1,356	370	0
Uganda	2013-2014	LSMS	264959	1,616	0	413
Zambia	1998	LCMS	14015	9,921	0	72
Zambia	1999	UNICEF MICS	14122	4,268	0	71
Zambia	2001-2002	DHS Program	21102	5,651	0	9
Zambia	2002-2003	LCMS	14027	6,488	0	72
Zambia	2004-2005	LCMS	14063	11,231	0	72
Zambia	2006	LCMS	14105	7,756	0	72
Zambia	2007	DHS Program	21117	5,423	319	0
Zambia	2009	Access to Act National Integrated Family Survey	162031	1,293	1287	0
Zambia	2010	LCMS	58660	10,768	0	72
Zambia	2013-2014	DHS Program	77516	11,854	719	0
Zimbabwe	1999	DHS Program	21151	2,784	217	0
Zimbabwe	2005-2006	DHS Program	21163	4,196	394	0
Zimbabwe	2009	UNICEF MICS	35493	6,241	0	10
Zimbabwe	2010-2011	DHS Program	55992	4,278	393	0
Zimbabwe	2014	UNICEF MICS	152720	9,588	0	10
Zimbabwe	2015	DHS Program	157066	5,014	399	0

3.1 Data excluded from model

Select data sources were excluded for the following reasons: missing survey weights for areal data, missing gender variables, insufficient age granularity (in months) for HAZ and WAZ calculation in children age 0-2 years, incomplete sampling (e.g., only children age 0-3 years measured), or untrustworthy data (as determined by the survey administrator or by user inspection). Within each source, polygon survey clusters with a sample size of one were excluded. Untrustworthy data refers specifically to the exclusion of six surveys for the reasons described here. Two datasets, the 2009-2010 Ghana Socioeconomic Panel Survey and the 2005 Burkina Faso Core Welfare Indicator Questionnaire (CWIQ) Survey, were excluded because the national prevalence values reported for one or more indicators were determined to be implausibly high based on the country-level trend seen in the seven other Ghana and six other Burkina Faso sources. In addition, the data were only resolved to the first administrative

subdivision. This combined with the very coarse spatial resolution make the data of minor utility for our geospatial purposes. Two additional sources, the 2014 Multiple Indicator Cluster Surveys (MICS) Kenya Kakamega and Bungoma surveys, were excluded because, according to the survey documentation, the "anthropometric data suffered from digit preference for both weight and height," meaning the measurements were rounded with preference for certain numbers in a way that introduced considerable bias. The 2015 Ethiopia Living Standards Measurement Study – Integrated Surveys on Agriculture (LSMS-ISA) was excluded because the low prevalence of child growth failure in the Ogaden region was determined to be unrealistic by specialists in the field of child nutrition. Lastly, the 2015 Egypt Special Demographic and Health Survey (DHS) was excluded due a to non-proportional sample allocation designed to estimate the prevalence of hepatitis and certain other NCD risk factors such that the survey sampling was not equivalent to the rest of the surveys.

doi:10.1038/nature25760

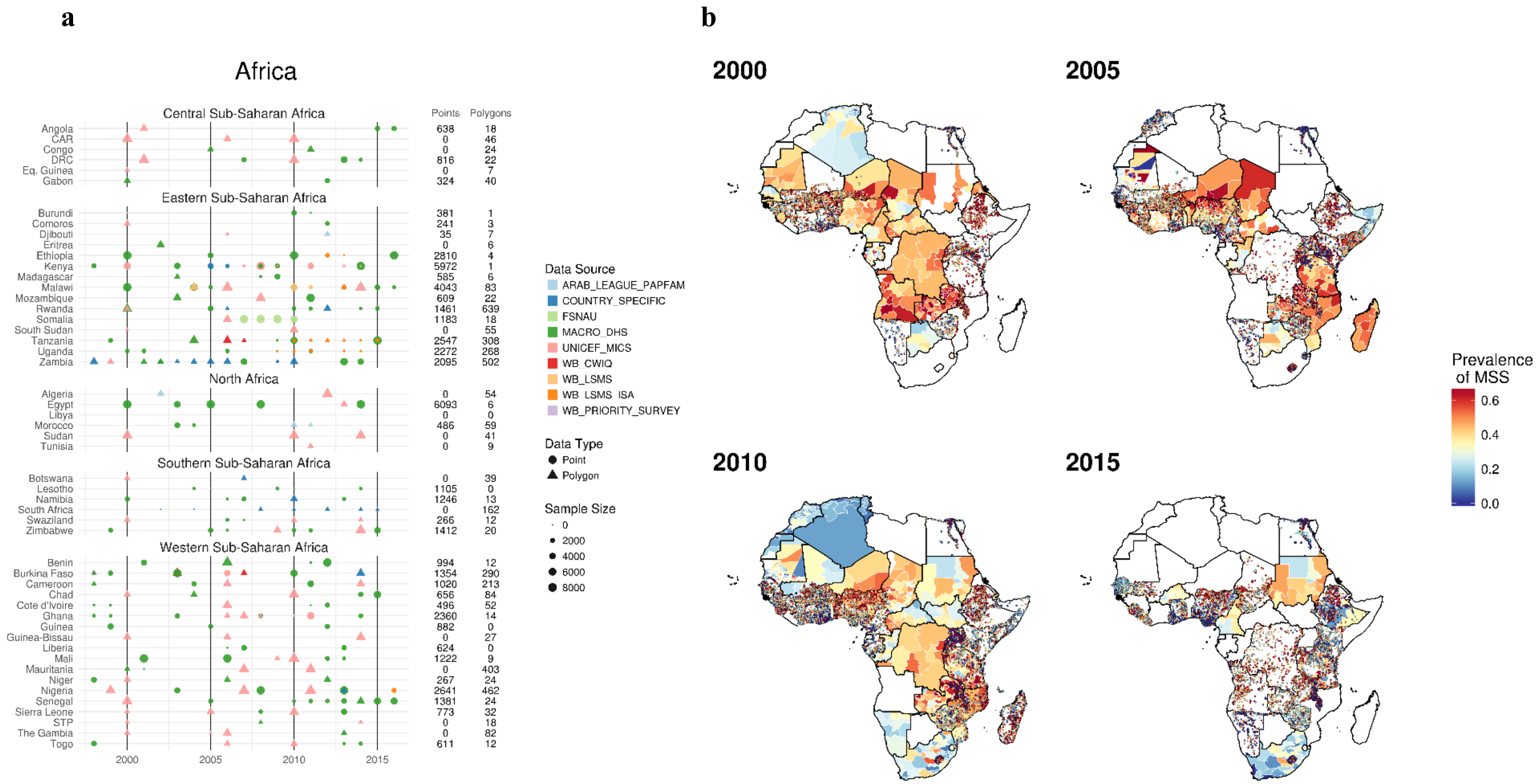

Supplementary Figure 1. Stunting data availability by type and country, 2000–2015.
All data are shown by country and year of survey and mapped at their corresponding geopositioned coordinate or area. The total number of points and polygons (areal) for each country are plotted by data source, type, and sample size (a). Sample size represents the number of individual microdata records for each survey. Mean stunting prevalence of the input coordinate or area is mapped (b). This database consists of 50,142 clusters and 4,253 polygons with a sample size totaling over 1.15 million children in Africa.

doi:10.1038/nature25760

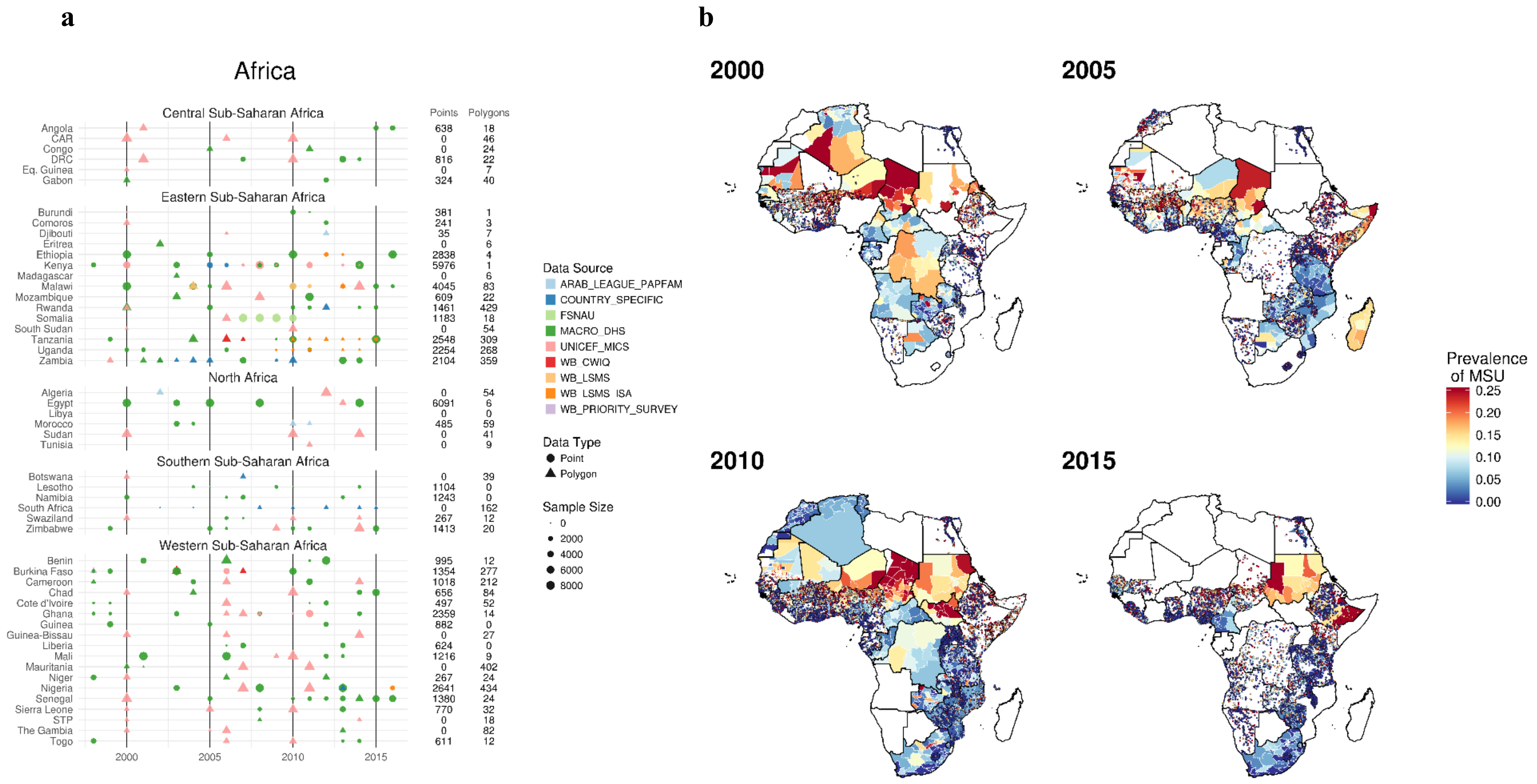

Supplementary Figure 2. Wasting data availability by type and country, 2000–2015.
All data are shown by country and year of survey and mapped at their corresponding geopositioned coordinate or area. The total number of points and polygons (areal) for each country are plotted by data source, type, and sample size (a). Sample size represents the number of individual microdata records for each survey. Mean stunting prevalence of the input coordinate or area is mapped (b). This database consists of 49,564 clusters and 3,844 polygons with a sample size totaling over 1.10 million children in Africa.

doi:10.1038/nature25760

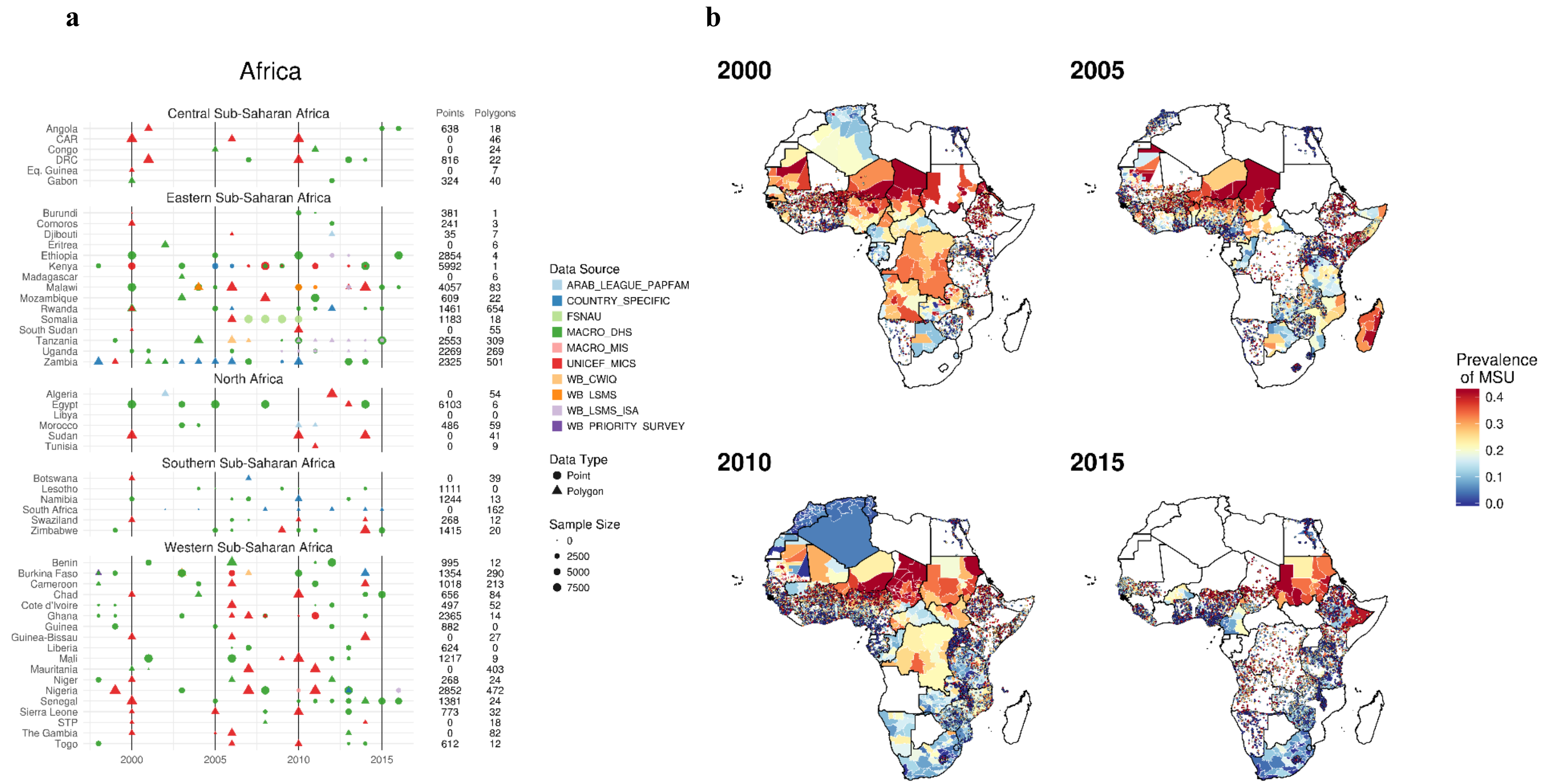

Supplementary Figure 3. Underweight data availability by type and country, 2000–2015.
All data are shown by country and year of survey and mapped at their corresponding geopositioned coordinate or area. The total number of points and polygons (areal) for each country are plotted by data source, type, and sample size (a). Sample size represents the number of individual microdata records for each survey. Mean stunting prevalence of the input coordinate or area is mapped (b). This database consists of 50,078 clusters and 4,279 polygons with a sample size of over 1.18 million children in Africa.

4.0 Supplementary covariates

A variety of socioeconomic and environmental variables were used to predict child growth failure outcomes. Where available, the finest spatio-temporal resolution of gridded data sets were used. In addition to the covariates detailed below, some country-level variables were included: lag distributed income per capita, and the proportion of the population with access to adequate sanitation, were included in models for stunting, wasting, and underweight. For wasting, the mortality rate due to famine, as produced by GBD 2016[11], was also included.

Supplementary Table 3. Covariates used in mapping

Covariate	Temporal resolution	Source	Reference
Aridity	Annual	Climatic Research Unit Time-Series (CRUTS)	Harris, I., Jones, P. d., Osborn, T. j. & Lister, D. h. Updated high-resolution grids of monthly climatic observations – the CRU TS3.10 dataset. *Int. J. Climatol*. **34**, 623–642 (2014).<br><br>University of East Anglia. Climatic Research Unit TS v. 3.24 dataset. Available at: https://crudata.uea.ac.uk/cru/data/hrg/cru_ts_3.24.01/. (Accessed: 24th July 2017).
Average daily maximum temperature	Annual	CRUTS	Harris, I., Jones, P. d., Osborn, T. j. & Lister, D. h. Updated high-resolution grids of monthly climatic observations – the CRU TS3.10 dataset. *Int. J. Climatol*. **34**, 623–642 (2014).<br><br>University of East Anglia. Climatic Research Unit TS v. 3.24 dataset. Available at: https://crudata.uea.ac.uk/cru/data/hrg/cru_ts_3.24.01/. (Accessed: 24th July 2017).
Average daily mean temperature	Annual	CRUTS	Harris, I., Jones, P. d., Osborn, T. j. & Lister, D. h. Updated high-resolution grids of monthly climatic observations – the CRU TS3.10 dataset. *Int. J. Climatol*. **34**, 623–642 (2014).<br><br>University of East Anglia. Climatic Research Unit TS v. 3.24 dataset. Available at: https://crudata.uea.ac.uk/cru/data/hrg/cru_ts_3.24.01/. (Accessed: 24th July 2017).
Average daily minimum temperature	Annual	CRUTS	Harris, I., Jones, P. d., Osborn, T. j. & Lister, D. h. Updated high-resolution grids of monthly climatic

Covariate	Temporal resolution	Source	Reference
			observations – the CRU TS3.10 dataset. *Int. J. Climatol*. **34**, 623–642 (2014).<br><br>University of East Anglia. Climatic Research Unit TS v. 3.24 dataset. Available at: https://crudata.uea.ac.uk/cru/data/hrg/cru_ts_3.24.01/. (Accessed: 24th July 2017).
Average Land Surface Temperature (LST)	Annual	MODIS	USGS & NASA. Land surface temperature and emissivity 8-day L3 global 1km MOD11A2 dataset. Available at: https://lpdaac.usgs.gov/dataset_discovery/modis/modis_products_table/mod11a2. (Accessed: 24th July 2017)<br><br>Wan, Z. MODIS Land-Surface Temperature Algorithm Theoretical Basis Document (LST ATBD).<br><br>Weiss, D. J. et al. An effective approach for gap-filling continental scale remotely sensed time-series. *Isprs J. Photogramm. Remote Sens*. **98**, 106–118 (2014).
Daytime LST	Annual	MODIS	USGS & NASA. Land surface temperature and emissivity 8-day L3 global 1km MOD11A2 dataset. Available at: https://lpdaac.usgs.gov/dataset_discovery/modis/modis_products_table/mod11a2. (Accessed: 24th July 2017)<br><br>Wan, Z. MODIS Land-Surface Temperature Algorithm Theoretical Basis Document (LST ATBD).<br><br>Weiss, D. J. et al. An effective approach for gap-filling continental scale remotely sensed time-series. *Isprs J. Photogramm. Remote Sens*. **98**, 106–118 (2014).
Distance to rivers	Static	Natural Earth Data (derived)	Natural Earth. Rivers and lake centerlines dataset. Available at: http://www.naturalearthdata.com/downloads/10m-physical-vectors/10m-rivers-lake-centerlines/. (Accessed: 24th July 2017)

Covariate	Temporal resolution	Source	Reference
Diurnal difference in LST	Annual	MODIS	USGS & NASA. Land surface temperature and emissivity 8-day L3 global 1km MOD11A2 dataset. Available at: https://lpdaac.usgs.gov/dataset_discovery/modis/modis_products_table/mod11a2. (Accessed: 24th July 2017)<br><br>Wan, Z. MODIS Land-Surface Temperature Algorithm Theoretical Basis Document (LST ATBD).<br><br>Weiss, D. J. et al. An effective approach for gap-filling continental scale remotely sensed time-series. *Isprs J. Photogramm. Remote Sens*. **98**, 106–118 (2014).
Diurnal temperature range	Annual	CRUTS	Harris, I., Jones, P. d., Osborn, T. j. & Lister, D. h. Updated high-resolution grids of monthly climatic observations – the CRU TS3.10 dataset. *Int. J. Climatol*. **34**, 623–642 (2014).<br><br>University of East Anglia. Climatic Research Unit TS v. 3.24 dataset. Available at: https://crudata.uea.ac.uk/cru/data/hrg/cru_ts_3.24.01/. (Accessed: 24th July 2017).
Enhanced Vegetation Index (EVI)	Annual	MODIS	Huete, A., Justice, C. & van Leeuwen, W. MODIS vegetation index (MOD 13) algorithm theoretical basis document. (1999).<br><br>USGS & NASA. Vegetation indices 16-Day L3 global 500m MOD13A1 dataset. Available at: https://lpdaac.usgs.gov/dataset_discovery/modis/modis_products_table/mod13a1. (Accessed: 25th July 2017)<br><br>Weiss, D. J. et al. An effective approach for gap-filling continental scale remotely sensed time-series. *Isprs J. Photogramm. Remote Sens*. **98**, 106–118 (2014).
Fertility	Annual	WorldPop (derived)	Lloyd, C. T., Sorichetta, A. & Tatem, A. J. High resolution global gridded data for use in population studies. *Sci. Data* **4**, sdata20171 (2017).

Covariate	Temporal resolution	Source	Reference
			World Pop. Get data. Available at: http://www.worldpop.org.uk/data/get_data/. (Accessed: 25th July 2017)
Frost day frequency	Annual	CRUTS	Harris, I., Jones, P. d., Osborn, T. j. & Lister, D. h. Updated high-resolution grids of monthly climatic observations – the CRU TS3.10 dataset. *Int. J. Climatol*. **34**, 623–642 (2014).<br><br>University of East Anglia. Climatic Research Unit TS v. 3.24 dataset. Available at: https://crudata.uea.ac.uk/cru/data/hrg/cru_ts_3.24.01/. (Accessed: 24th July 2017)
Growing season length	Static	FAO	FAO. GAEZ - Global Agro-Ecological Zones data portal. Available at: http://www.fao.org/nr/gaez/about-data-portal/en/. (Accessed: 25th July 2017)<br><br>FAO. GAEZ - Global Agro-Ecological Zones users guide. (2012).
Irrigation	Static	University of Frankfurt	Goethe-Universität. Generation of a digital global map of irrigation areas. Available at: https://www.uni-frankfurt.de/45218039/Global_Irrigation_Map. (Accessed: 25th July 2017)
Malaria incidence	Annual	Malaria Atlas Project	Bhatt, S. et al. The effect of malaria control on *Plasmodium falciparum* in Africa between 2000 and 2015. *Nature* **526**, 207–211 (2015).
Educational attainment in women of reproductive age (15-49 years old)	Annual	Institute for Health Metrics and Evaluation, University of Washington	Graetz, N. *et al*. Mapping local variation in educational attainment across Africa. *Nature* https://doi.org/10.1038/nature25761 (2018).
Nighttime LST	Annual	MODIS	USGS & NASA. Land surface temperature and emissivity 8-day L3 global 1km MOD11A2 dataset. Available at: https://lpdaac.usgs.gov/dataset_discovery/modis/modis_products_table/mod11a2. (Accessed: 24th July 2017)

Covariate	Temporal resolution	Source	Reference
			Wan, Z. MODIS Land-Surface Temperature Algorithm Theoretical Basis Document (LST ATBD).<br><br>Weiss, D. J. et al. An effective approach for gap-filling continental scale remotely sensed time-series. *Isprs J. Photogramm. Remote Sens.* **98**, 106–118 (2014).
Nighttime lights	Annual	NOAA DMSP	Savory et al. Intercalibration and Gaussian Process Modeling of Nighttime Lights Imagery for Measuring Urbanization Trends in Africa 2000–2013. *Remote Sens.* **9**, (2017).
Normalized Difference Vegetation Index (NDVI)	Annual	AVHRR	NASA & NOAA. Advanced Very High Resolution Radiometer (AVHRR) Normalized Difference Vegetation Index (NDVI) dataset. Available at: https://nex.nasa.gov/nex/projects/1349/. (Accessed: 25th July 2017)
Nutritional yield for calcium	Static	Herrero et al (modelled)	Herrero, M. et al. Farming and the geography of nutrient production for human use: a transdisciplinary analysis. *Lancet Planet. Health* **1**, e33–e42 (2017).
Nutritional yield for folate	Static	Herrero et al (modelled)	Herrero, M. et al. Farming and the geography of nutrient production for human use: a transdisciplinary analysis. *Lancet Planet. Health* **1**, e33–e42 (2017).
Nutritional yield for iron	Static	Herrero et al (modelled)	Herrero, M. et al. Farming and the geography of nutrient production for human use: a transdisciplinary analysis. *Lancet Planet. Health* **1**, e33–e42 (2017).
Nutritional yield for protein	Static	Herrero et al (modelled)	Herrero, M. et al. Farming and the geography of nutrient production for human use: a transdisciplinary analysis. *Lancet Planet. Health* **1**, e33–e42 (2017).
Nutritional yield for vitamin A	Static	Herrero et al (modelled)	Herrero, M. et al. Farming and the geography of nutrient production for human use: a transdisciplinary analysis. *Lancet Planet. Health* **1**, e33–e42 (2017).
Nutritional yield for vitamin B12	Static	Herrero et al (modelled)	Herrero, M. et al. Farming and the geography of nutrient production for human use: a transdisciplinary analysis. *Lancet Planet. Health* **1**, e33–e42 (2017).

Covariate	Temporal resolution	Source	Reference
Nutritional yield for zinc	Static	Herrero et al (modelled)	Herrero, M. et al. Farming and the geography of nutrient production for human use: a transdisciplinary analysis. *Lancet Planet. Health* **1**, e33–e42 (2017).
Population	Annual	WorldPop	Lloyd, C. T., Sorichetta, A. & Tatem, A. J. High resolution global gridded data for use in population studies. *Sci. Data* **4**, sdata20171 (2017).<br><br>World Pop. Get data. Available at: http://www.worldpop.org.uk/data/get_data/. (Accessed: 25th July 2017)
Potential Evapotranspiratio n (PET)	Annual	CRUTS	Harris, I., Jones, P. d., Osborn, T. j. & Lister, D. h. Updated high-resolution grids of monthly climatic observations – the CRU TS3.10 dataset. *Int. J. Climatol*. **34**, 623–642 (2014).<br><br>University of East Anglia. Climatic Research Unit TS v. 3.24 dataset. Available at: https://crudata.uea.ac.uk/cru/data/hrg/cru_ts_3.24.0 1/. (Accessed: 24th July 2017).
Precipitation	Annual	CRUTS	Harris, I., Jones, P. d., Osborn, T. j. & Lister, D. h. Updated high-resolution grids of monthly climatic observations – the CRU TS3.10 dataset. *Int. J. Climatol*. **34**, 623–642 (2014).<br><br>University of East Anglia. Climatic Research Unit TS v. 3.24 dataset. Available at: https://crudata.uea.ac.uk/cru/data/hrg/cru_ts_3.24.0 1/. (Accessed: 24th July 2017).
Shannon Diversity Index for nutrient yield	Static	Herrero et al (modelled)	Herrero, M. *et al*. Farming and the geography of nutrient production for human use: a transdisciplinary analysis. *Lancet Planet. Health* **1**, e33–e42 (2017).
Soil organic carbon stock	Static	ISRIC	Hengl, T. et al. SoilGrids250m: Global gridded soil information based on machine learning. *PLOS ONE* **12**, e0169748 (2017).<br><br>ISRIC World Soil Information. Explore Layers. Available at: http://geonode.isric.org/layers/?limit=100&offset= 0. (Accessed: 25th July 2017)

Covariate	Temporal resolution	Source	Reference
Soil pH	Static	ISRIC	Hengl, T. et al. SoilGrids250m: Global gridded soil information based on machine learning. *PLOS ONE* **12**, e0169748 (2017).<br><br>ISRIC World Soil Information. Explore Layers. Available at: http://geonode.isric.org/layers/?limit=100&offset=0. (Accessed: 25th July 2017)
Tassled cap brightness	Annual	MODIS	USGS & NASA. Nadir BRDF- Adjusted Reflectance Reflectance 16-Day L3 Global 1km dataset. Available at: https://lpdaac.usgs.gov/dataset_discovery/modis/modis_products_table/mcd43b4. (Accessed: 25th July 2017)<br><br>Strahler, A. H. & Muller, J.-P. MODIS BRDF/Albedo product: algorithm theoretical basis document version 5.0. (1999).<br><br>Weiss, D. J. et al. An effective approach for gap-filling continental scale remotely sensed time-series. *Isprs J. Photogramm. Remote Sens*. **98**, 106–118 (2014).
Tassled cap wetness	Annual	MODIS	USGS & NASA. Nadir BRDF- Adjusted Reflectance Reflectance 16-Day L3 Global 1km dataset. Available at: https://lpdaac.usgs.gov/dataset_discovery/modis/modis_products_table/mcd43b4. (Accessed: 25th July 2017)<br><br>Strahler, A. H. & Muller, J.-P. MODIS BRDF/Albedo product: algorithm theoretical basis document version 5.0. (1999).
Travel time to nearest settlement >50,000 inhabitants	Static	Malaria Atlas Project, Big Data Institute, Nuffield Department of Medicine, University of Oxford	Weiss, D. J. *et al*. A global map of travel time to cities to assess inequalities in accessibility in 2015. *Nature* **533**, 333-336 (2018).

Covariate	Temporal resolution	Source	Reference
Urbanicity	Annual	European Commission/ GHS	Pesaresi, M. et al. Operating procedure for the production of the Global Human Settlement Layer from Landsat data of the epochs 1975, 1990, 2000, and 2014. (Publications Office of the European Union, 2016).
Wet day frequency	Annual	CRUTS	Harris, I., Jones, P. d., Osborn, T. j. & Lister, D. h. Updated high-resolution grids of monthly climatic observations – the CRU TS3.10 dataset. *Int. J. Climatol*. **34**, 623–642 (2014).<br><br>University of East Anglia. Climatic Research Unit TS v. 3.24 dataset. Available at: https://crudata.uea.ac.uk/cru/data/hrg/cru_ts_3.24.01/. (Accessed: 24th July 2017).

doi:10.1038/nature25760

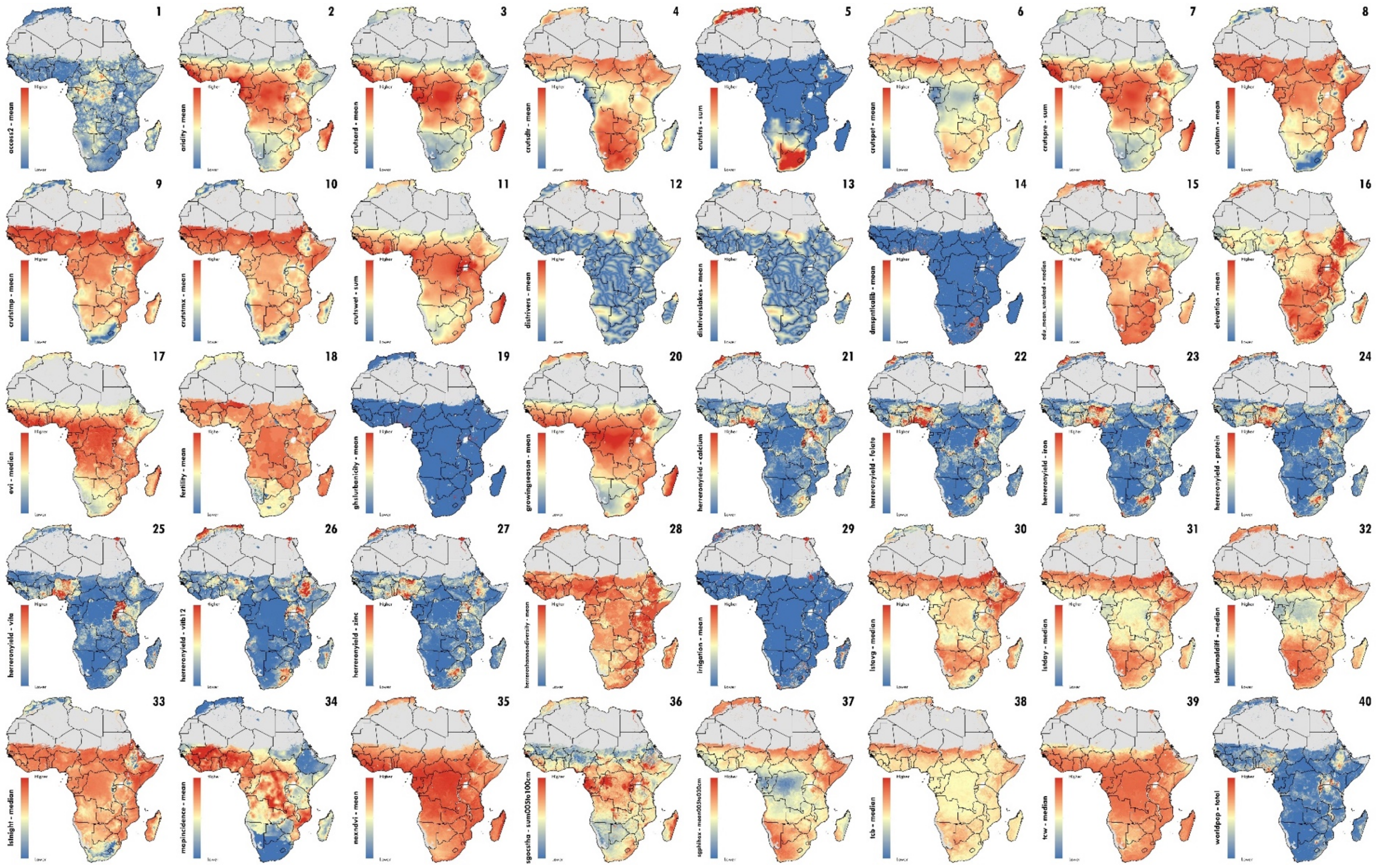

Supplementary Figure 4. Covariates.

Thirty-seven covariate raster layers of possible socioeconomic and environmental correlates of CGF in Africa were used as inputs for the stacking modelling process. Time-varying covariates are presented for the year 2015. For the year of production of non-time-varying covariates, please refer to the individual covariate citation in Supplementary Table 3 for additional detail.

5.0 Supplementary methods

5.1 Seasonality adjustment

Weight-for-height z-scores (WHZ) are used to calculate an individual's moderate and severe binary wasting status. As a data preprocessing step, we performed a seasonality adjustment on individual-level child weights in order to account for differences in observed child weight that may have been due to food scarcity around the month in which the survey was conducted. To adjust weight measurements, we fit a model for each region (Extended Data Fig. 2) with a 12-month seasonal spline, a country fixed effect, and a smooth spline over the duration of our data collection using the *mgcv* package in R and the following formula:

$$WHZ \sim s_{cc}(month) + s_{tp}(t) + as.factor(country).$$

WHZ is a child's weight-for-height z-score, $month$ is integer-valued month of the year (1, …, 12), t is the time of the interview in integer months since the earliest observation of any child in the dataset, and $country$ is a factor variable representing the country where the observation was recorded. We modelled the periodic component on months using 12 cyclic cubic regression splines basis functions (cc) and we accounted for a smooth longer time temporal trend using four thin-plate splines (tp). The country effects and the long-term temporal spline were included only to help avoid confounding during fitting of the seasonal spline fit and neither country effects nor the long-term trend were used in the seasonal adjustment. We then adjusted all observations to account for the difference in the seasonal period between the month of the interview and an average day of the year as determined by which days align with the mean of the periodic spline. See Supplementary Figure 5 for an example and visualisation of how individual data points are adjusted to the periodic mean. The impact of this adjustment on the prevalence of wasting is minor, and is visualised in Supplementary Figure 6.

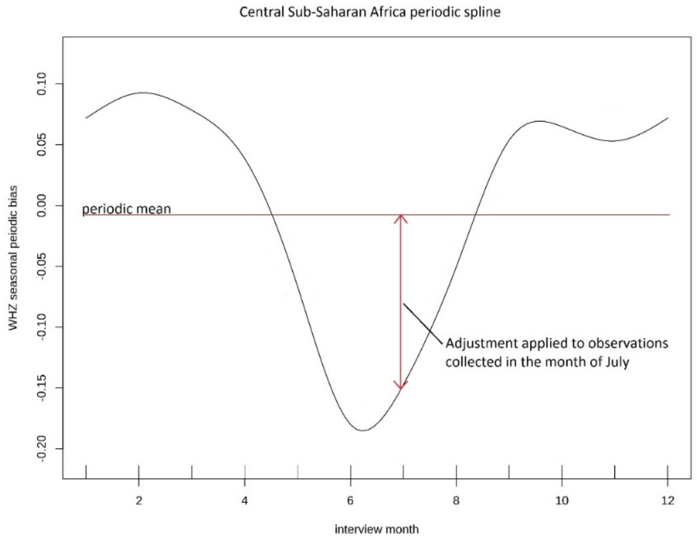

Supplementary Figure 5. Periodic seasonality adjustment.
The fitted seasonal periodic spline for wasting in central sub-Saharan Africa along with the mean of the periodic function and an example of the adjustment that would be applied to weight-for-height z-scores collected in this region in the month of July.

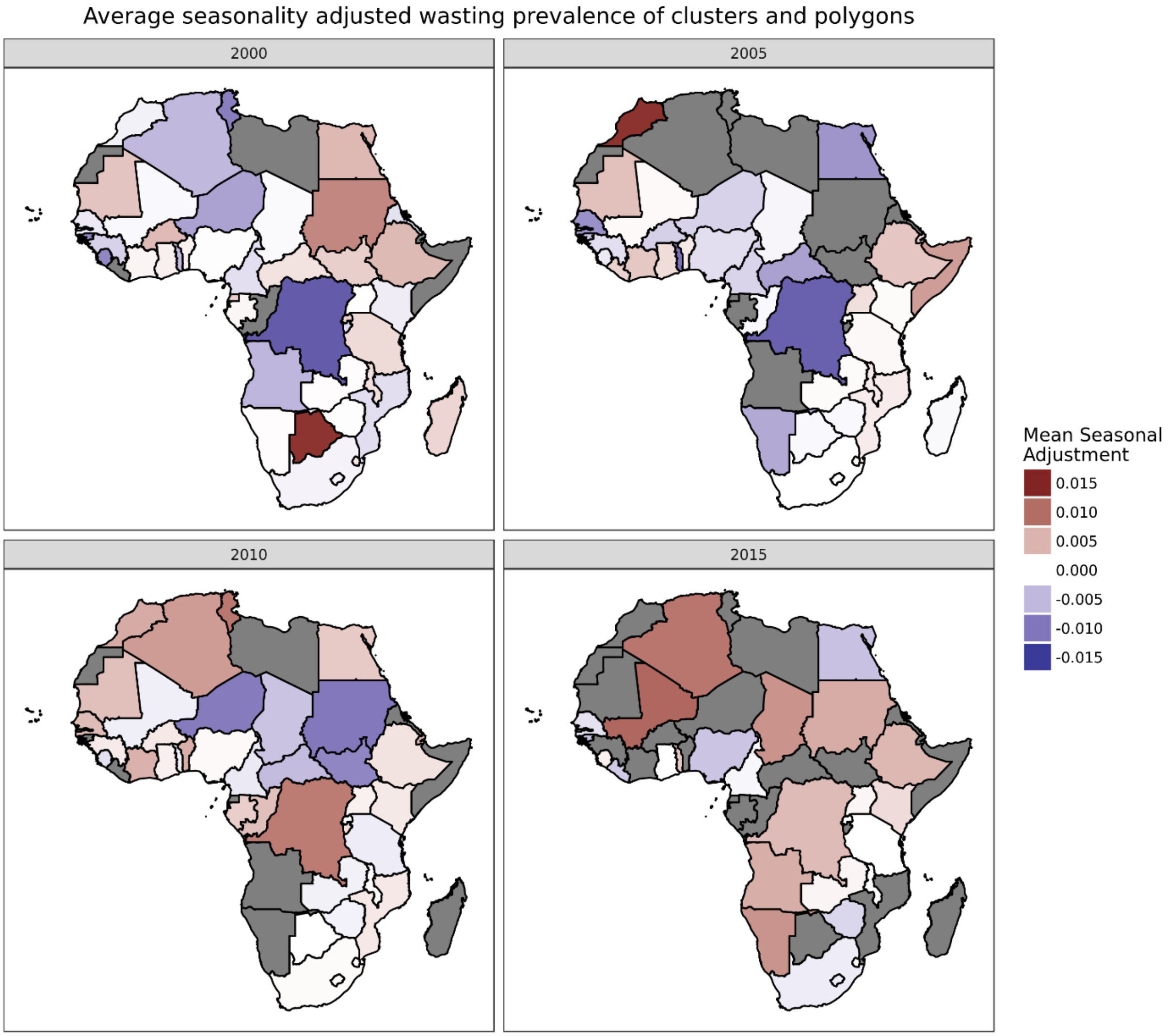

Supplementary Figure 6. Cluster and polygon-level seasonality wasting adjustments. The sample-size weighted mean cluster and polygon seasonal prevalence adjustments were averaged within countries and five-year periods. Areas shown in grey reflect geographic regions in which no data were observed for that country within that time period.

5.2 Cluster combination and spatial integration over polygon records

Our individual-level data were collapsed (summarised) into clusters if they could be georeferenced to latitude-longitude pairs. Otherwise, we collapsed our individual-level data to the smallest polygon that could be referenced. We used survey weights and the *survey* package in R to account for matching our observations to a higher resolution than the representative resolution of the survey.[12] The *survey* package was used to adjust the mean response in the higher resolution polygons using post-stratification weighted averages,[13] but we found that the package somewhat regularly produced very large design effects estimates that rendered our polygon sample size adjustments to be nonsensical. Instead, we use the classic Kish's effective sample size calculation where the effective sample size for a polygon is calculated as:

$$n_{eff} = \frac{(\sum w_i)^2}{\sum w_i^2},$$

where w_i is the survey weight associated with data observation i, and the summations in the effective sample size calculation are both taken over all observation within a polygon.[14]

Data without latitude and longitude, but that could be geolocated to an administrative area, were resampled to generate candidate point locations based on the underlying population of the administrative area. The main concept is to leverage covariate values across the polygon when performing the regression, while simultaneously accounting for a population-driven survey design. The methods used for the resampling are consistent with those used in geospatial modelling of under-5 mortality, published previously.[15]

For each polygon-level observation, 10,000 points were randomly sampled from within the polygon (regardless of the polygon's area) using the WorldPop total population raster[16,17] to weight the locations of the draws. K-means clustering was performed on the candidate points to generate integration points (1 per 1,000 pixels) used in the modelling. Weights were assigned to each integration point proportionally to the number of candidate points that entered into the k-means cluster, such that the weight of each point represented the number of population-sampled locations contained within the K-means cluster location, divided by the number of sampled points generated (10,000). Each point generated by this process is assigned the prevalence of CGF observed from the survey for that polygon. These sample weights are used in model fit (see additional detail on ensemble covariate modelling and model fitting and estimate generation in the Supplementary Methods).

Supplementary Figure 7 shows examples of this resampling process for a survey in which data existed for the administrative area of Komo, Gabon. The polygon resampling process was repeated three times to show how the spread of point locations, and the magnitude of the weights generated using K-means clustering, compare across different iterations of the point-generation process (in which point locations are generated at random, such that more populated areas are more likely to have been "sampled"). Higher sample weights from the K-means clustering process are observed in each iteration near the more densely populated areas of the Komo Department.

doi:10.1038/nature25760

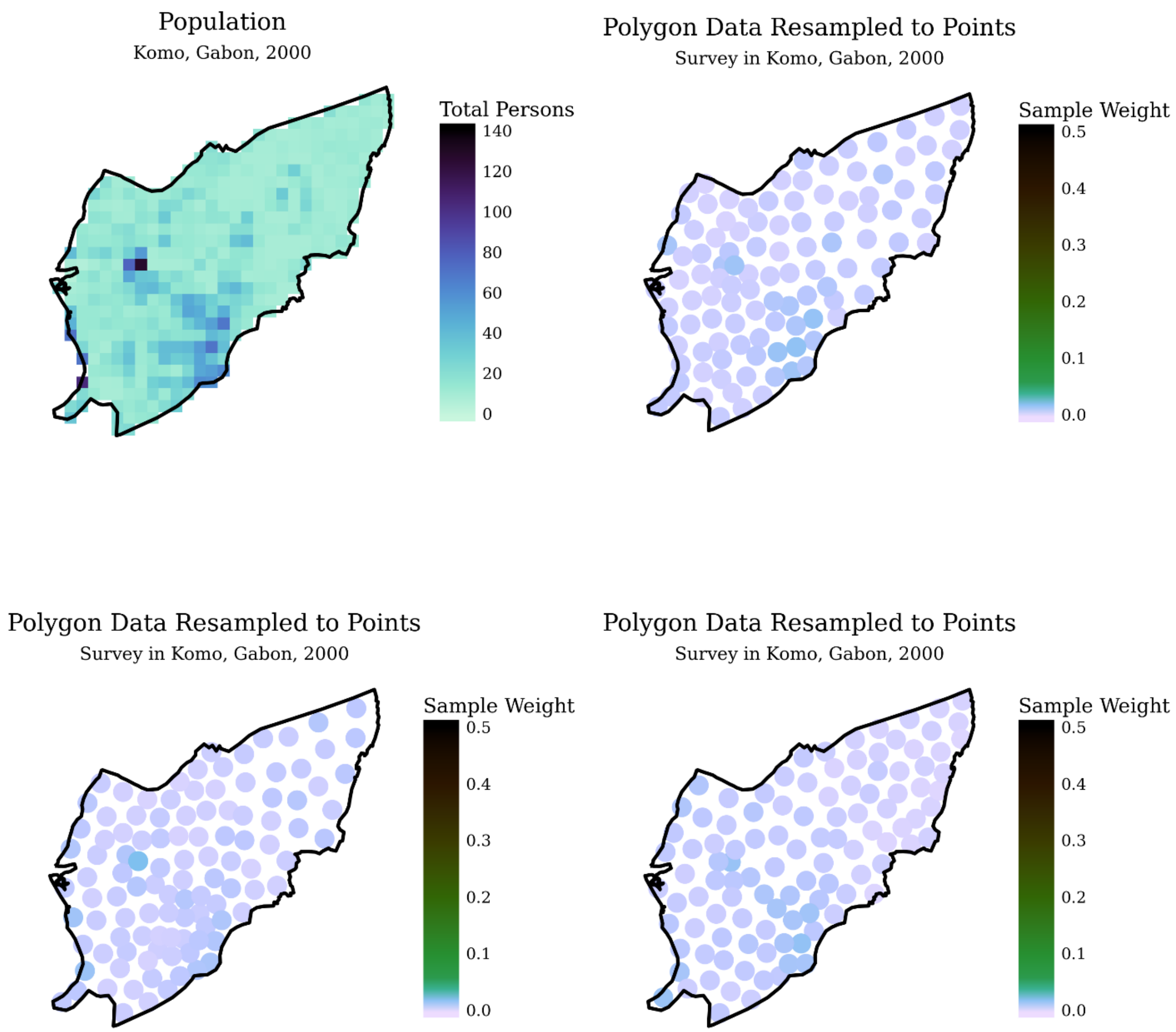

Supplementary Figure 7. Polygon resampling.
The total population in Komo, Gabon, in the year 2000 (top left), and three different example iterations of the polygon resampling process for a survey undertaken in 2000 that contained data for Komo, Gabon.

5.3 Geostatistical model

5.3.1 Model geographies

A total of five models were run for each indicator based on continuous geographic regions within Africa chosen to align with the regions used in the Global Burden of Disease Study, which determines regions based on both proximity and epidemiological similarity (see tables in Supplementary Fig. 1-3 for listing of regions and countries). Minor changes were made to the GBD regions to ensure spatial contiguity across Africa (see Extended Data Fig. 2 for an illustration of the modelling regions). All data within the spatial region, and within a one-degree buffer from the boundaries of each region, were included in each model to minimise edge effects.

5.3.2 Ensemble covariate modelling

An ensemble covariate modelling method was implemented in order to select covariates and capture possible non-linear effects and complex interactions between them.[27] For each region, three sub-models were fit to our dataset, using all of our covariate data as explanatory predictors: generalised additive models, boosted regression trees, and lasso regression. Country level fixed effects were also included in the boosted regression tree model as dummy-coded covariates. Sample weights are used in sub-models, where applicable, such that cluster locations with latitude and longitude had a sample weight of 1, while cluster locations where the latitude and longitude was generated by the polygon resampling process had a weight based on the K-means clustering process (see 5.2, Cluster combination and spatial integration over polygon records).

Each sub-model is fit using five-fold cross-validation to avoid overfitting. The out-of-sample predictions from across the five holdouts are compiled into a single comprehensive set of predictions from that model. Additionally, the same sub-models were also run using 100% of the data, and a full set of in-sample predictions were created. The five sets of out-of-sample sub-model predictions are fed into the full geostatistical model as the explanatory covariates when performing the model fit. The in-sample predictions from the sub-models are used as the covariates when generating predictions using the fitted full geostatistical model. A recent study has shown that this ensemble approach can improve predictive validity by up to 25% over an individual model.[27]

Predictions from each sub-model are generated based on patterns and relationships between the raw covariates and prevalence survey data, while predictions from the full geostatistical model are generated based on patterns and relationships between the predictions from the ensemble of sub-models and prevalence survey data. To discover the relationships between the sub-model prediction layers (used as covariates in the full geostatistical model) and the prevalence data, the only values of the covariates (sub-model prediction layers) “seen” by the model are the values underlying the locations of surveys. As such, it is possible that estimates will be generated in areas where the values of the covariates exceed the minimum and maximum values observed by

the model. In these areas, the estimates are generated by extrapolating from the patterns observed within the range of covariates underlying the survey data.

Supplementary Fig. 8-10 illustrate that very few estimates were generated due to the extrapolation of covariate values. The maps on the right panel of the graphics shows the total number of instances (summed over each of the sub-models, for each year of covariate values, from 2000 to 2010) that the model extrapolated estimates for each 5x5 km pixel, divided by the total number of sub-model-years in which predictions were generated. The stacked bar chart on the left shows the number of pixels in which the model extrapolated estimates, divided by the total number of pixels within sub-models, and across all years, broken down by sub-model type.

Supplementary Fig. 8-10 are calculated in two ways: such that the covariate values at each pixel are compared to the range observed in the full time series for each modelling region (top panel), and to the range observed within each year, for each modelling region (bottom panel). When the minimum and maximum covariate values are calculated across the full time series of the data (top panel), it is revealed that very few of the pixels were extrapolated when considering the range of covariates across the time series. The same figure, calculated such that pixel covariate values are compared to the minimum and maximum observed within each given year (bottom panel), reveals more nuance and shows that estimates for pixels were frequently generated based on patterns observed in the full time series of the data, but not within the year the estimates were generated for.

doi:10.1038/nature25760

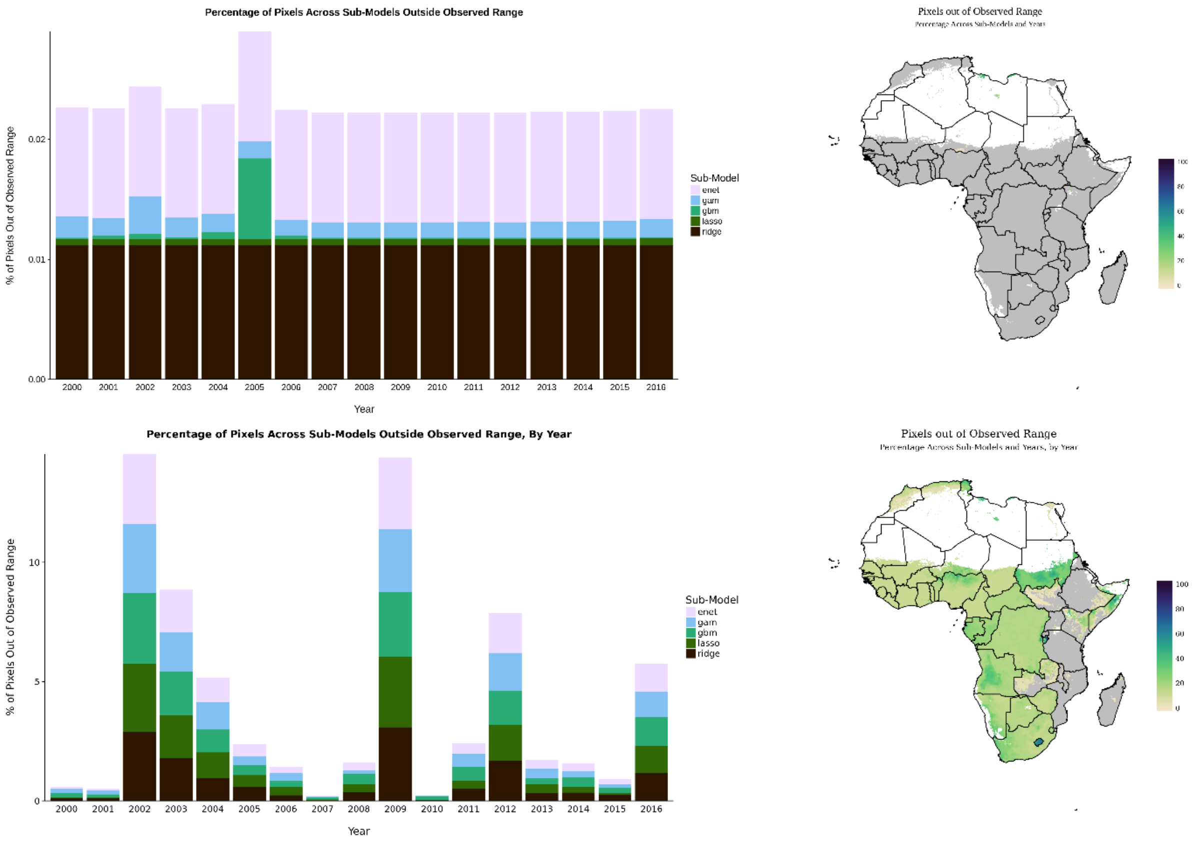

Supplementary Figure 8. Stunting covariate extrapolation.
The map (right) shows the percentage of year-sub-model combinations for each pixel that required extrapolation of covariate patterns for prediction generation within the full geostatistical model. The stacked bar chart (left) shows the percentage of pixels extrapolated out of the total number of pixels from all sub-models that were extrapolated, broken down by sub-model type, for each year. This graphic is calculated two ways: such that the covariate values at each pixel are compared to the minimum and maximum of the covariate values observed across all time (top) and observed within the year for which the estimates were generated (bottom). Areas in grey reflect pixels in which no sub-model-years required extrapolation.

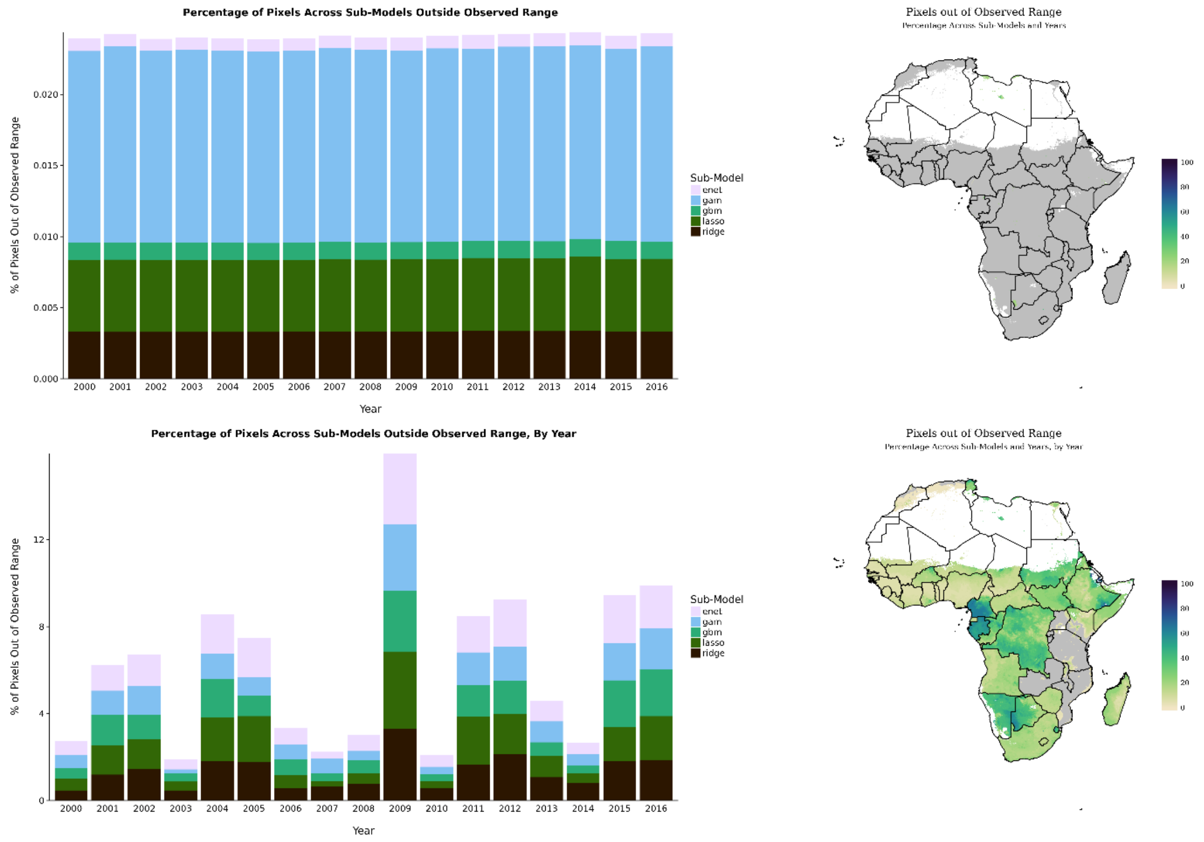

Supplementary Figure 9. Wasting covariate extrapolation.
The map (right) shows the percentage of year-sub-model combinations for each pixel that required extrapolation of covariate patterns for prediction generation within the full geostatistical model. The stacked bar chart (left) shows the percentage of pixels extrapolated out of the total number of pixels from all sub-models that were extrapolated, broken down by sub-model type, for each year. This graphic is calculated two ways: such that the covariate values at each pixel are compared to the minimum and maximum of the covariate values observed across all time (top) and observed within the year for which the estimates were generated (bottom). Areas in grey reflect pixels in which no sub-model-years required extrapolation.

doi:10.1038/nature25760

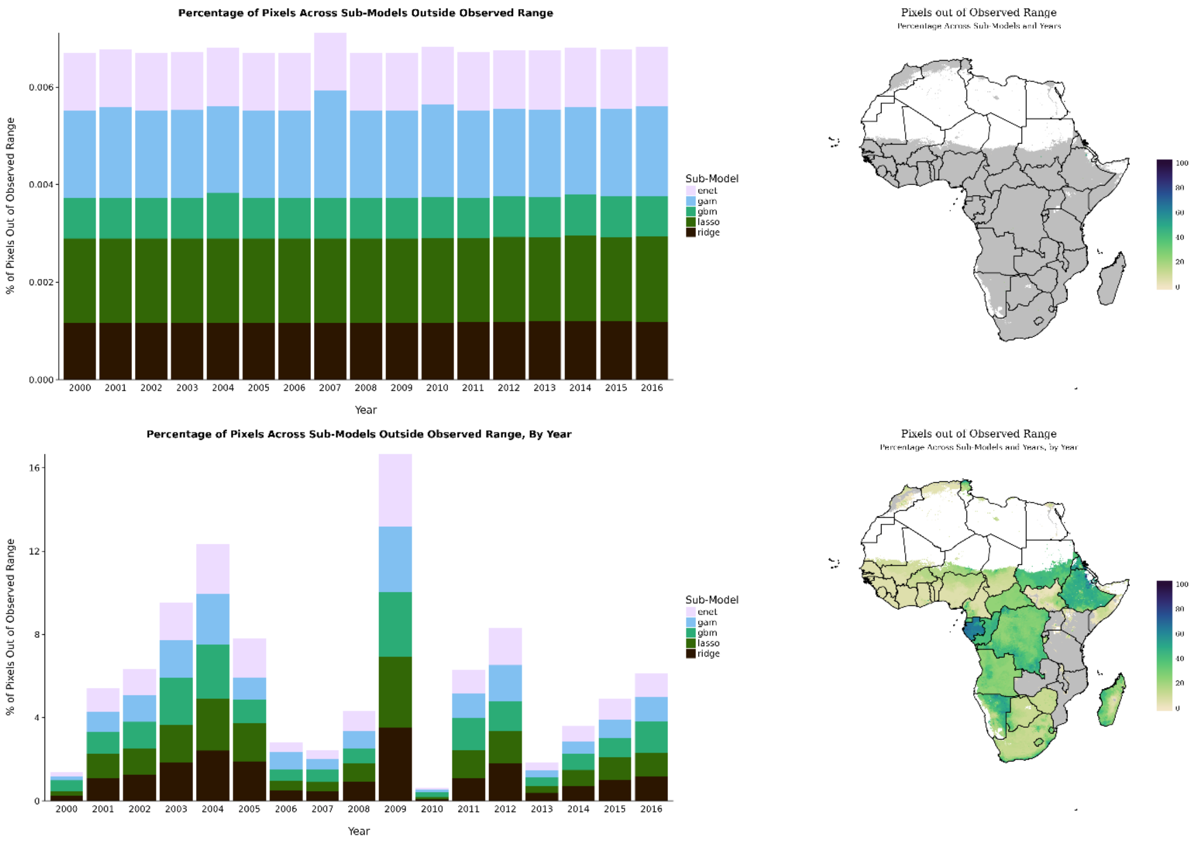

Supplementary Figure 10. Underweight covariate extrapolation.
The map (right) shows the percentage of year-sub-model combinations for each pixel that required extrapolation of covariate patterns for prediction generation within the full geostatistical model. The stacked bar chart (left) shows the percentage of pixels extrapolated out of the total number of pixels from all sub-models that were extrapolated, broken down by sub-model type, for each year. This graphic is calculated two ways: such that the covariate values at each pixel are compared to the minimum and maximum of the covariate values observed across all time (top) and observed within the year for which the estimates were generated (bottom). Areas in grey reflect pixels in which no sub-model-years required extrapolation.

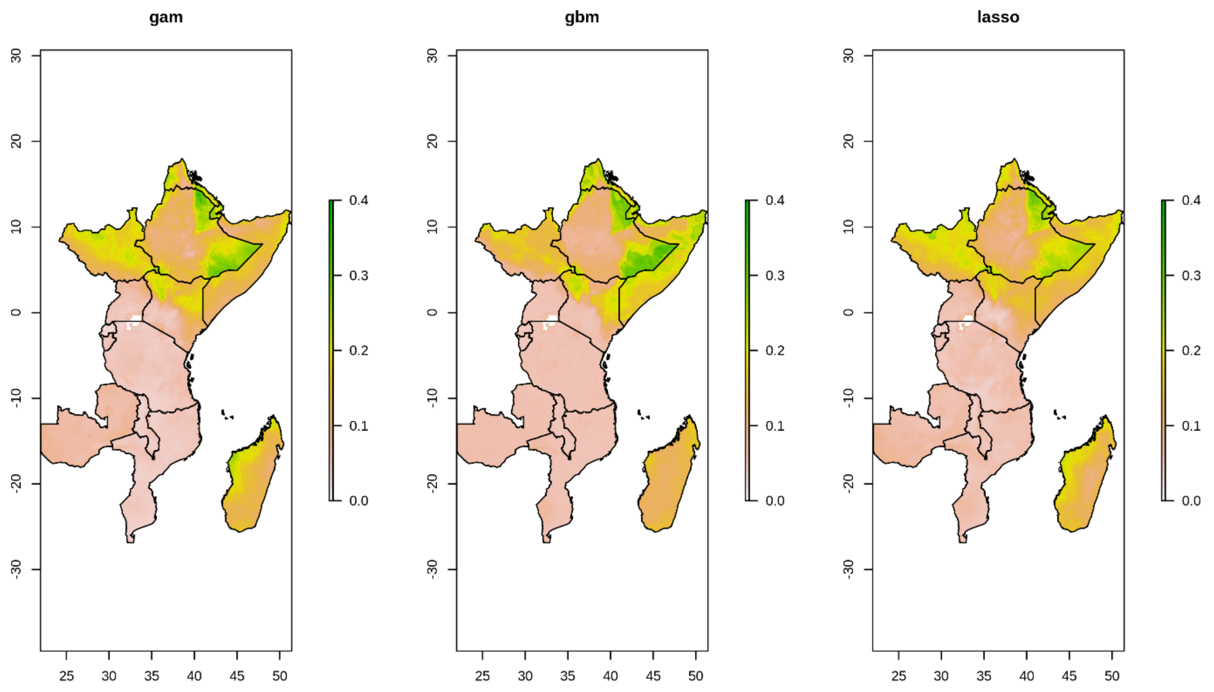

Supplementary Figure 11. Ensemble predicted rasters.
Predicted 2015 rasters, for use as covariates in the INLA modelling, shown for the eastern sub-Saharan Africa region. The *gam* plot shows the predictions from a generalised additive model fit, the *gbm* plot shows the predictions from a boosted regression tree fit, the *lasso* plot shows the predictions from a lasso penalised regression model fit.

5.3.3 Model description

Binomial count data are modelled within a Bayesian hierarchical modelling framework using a logit link function and a spatially and temporally explicit hierarchical generalised linear regression model to fit prevalence of each of our indicators in five regions of Africa as defined in GBD (Northern, Western, Southern, Central, and Eastern; see Extended Data Fig. 2).[18] For each GBD region, we explicitly write the hierarchy that defines our Bayesian model as follows:

$$C_i | p_i,\ N_i \sim Binomial(p_i,\ N_i)$$

$$logit(p_i) = \ \beta_0 + \boldsymbol{X_i\beta} + \epsilon_{GP_i} + \epsilon_i$$

$$\sum \boldsymbol{\beta} \ = 1$$

$$\epsilon_i \sim N(0, \sigma_{ug}^2)$$

$$\epsilon_{GP} | \Sigma_{\text{space}}, \ \Sigma_{time} \sim GP(0,\ \Sigma_{\text{space}} \otimes \ \Sigma_{time})$$

$$\Sigma_{\text{space}} = \frac{2^{1-\nu}}{\tau \times \Gamma(\nu)} \times (\kappa \boldsymbol{D})^{\nu} \times \mathrm{K}_{\nu}(\kappa \boldsymbol{D})$$

$$\Sigma_{time_{j,k}} = \rho^{|\, t_k - t_j|}.$$

For each risk factor and region, we model the number of children at cluster i, among a sample size, N_i, who are afflicted with a risk factor as binomial count data, C_i. We have suppressed the notation, but the counts, C_i, probabilities, p_i, predictions from the three submodels $\boldsymbol{X_i}$, and residual terms ϵ_* are all indexed at a space-time coordinate. The probabilities, p_i represent both the annual prevalence at the space-time location and the probability that an individual child will be afflicted with the risk factor given that they live at that particular location. The logit of annual prevalence, p_i, of our indicators was modelled as a linear combination of the three sub-models (GAM, BRT, and lasso), $\boldsymbol{X_{i,}}$a correlated spatiotemporal error term, ϵ_{GP_i}, and an independent nugget effect, ϵ_i. Coefficients, $\boldsymbol{\beta}$, on the sub-models represent their respective predictive weighting in the mean logit link and are constrained to sum to 1. In order for this constraint to make any sense we ensure that the predictions from the sub-models enter into INLA in the link space (logit) without having been centre-scaled. The joint error term, ϵ_{GP}, accounts for residual spatiotemporal autocorrelation between individual data points that remains after accounting for the predictive effect of the sub-model covariates, and the nugget, ϵ_i, which is an independent error term for each data point, representing measurement error for that observation. The residuals, ϵ_{GP}, are modelled as a three-dimensional Gaussian process in space-time centered at zero and with a covariance matrix constructed from a Kroenecker product of spatial and temporal covariance kernels. The spatial covariance, Σ_{space}, is modelled using an isotropic and stationary Matérn function,[19] and temporal covariance, Σ_{time}, as an annual autoregressive order 1 (AR1) function over the 17 years represented in the model. This approach leveraged the data's residual correlation structure to more accurately predict prevalence estimates for locations with no data,

while also propagating the dependence in the data through to uncertainty estimates.[20] The posterior distributions were fit using computationally efficient and accurate approximations in R-INLA[21,22] (integrated nested Laplace approximation) with the stochastic partial differential equations (SPDE)[23] approximation to the Gaussian process residuals. Pixel-level uncertainty intervals (UIs) were generated from 1,000 draws (i.e., statistically plausible candidate maps)[24] created from the posterior-estimated distributions of modelled parameters.

5.3.4 Priors

The following priors were used for all three of our child growth failure models:

- $\beta_0 \sim N(\mu = 0, \sigma^2 = 3^2)$,
- $\boldsymbol{\beta} \sim^{iid} N\left(\mu = \frac{1}{\text{\# ensemble models}}, \sigma^2 = 3^2\right)$,
- $log\left(\frac{1+\rho}{1-\rho}\right) \sim N(\mu = 0, \sigma^2 = 1/0.15)$,
- $log\left(\frac{1}{\sigma^2_{nug}}\right) \sim loggamma(\alpha = 1, \gamma = 2)$.
- $\theta_1 = \log(\tau) \sim N(\mu_{\theta_1}, \sigma^2_{\theta_1})$
- $\theta_2 = \log(\kappa) \sim N(\mu_2, \sigma^2_{\theta_2})$.

Given that our covariates used in INLA, i.e. the predicted outputs from the ensemble models, should be on the same scale as our predictive target, we believe that the intercept in our model should be close to zero and that the regression coefficients should sum to one. As such, we have chosen the prior for our intercept to be $\mathrm{N}(0, \sigma^2 = 3^2)$, and the prior for the fixed effect coefficients to be $\mathrm{N}(\frac{1}{\text{\# ensemble models}}, \sigma^2 = 3^2)$. The prior on the temporal correlation parameter ρ is chosen to be mean zero, showing no prior preference for either positive or negative auto-correlation structure, and with a distribution that is wide enough such that within three standard deviations of the mean the prior includes values of ρ ranging from -0.95 to 0.95. The priors on the random effects variances were chosen to be relatively loose given that we believe our fixed effects covariates should be well correlated with our outcome of interest, which might suggest relatively small random effects values. At the same time, we wanted to avoid using a prior that was so diffuse as to actually put high prior weight on large random effect variances. For stability, we used the uncorrelated multivariate normal priors that INLA automatically determines (based on the finite elements mesh) for the log-transformed spatial hyperparameters κ and τ. The mean and variance parameters for the hyperpriors selected by INLA for the meshes in each region can be found in Supplementary Table 4. In our parameterization we represent α and γ in the *loggamma* distribution as scale and shape, respectively.

Supplementary Table 4. Spatial hyperparameter priors by region

Region	μ_{θ_1}	$\sigma^2_{\theta_1}$	μ_2	$\sigma^2_{\theta_2}$
**Central sub-Saharan Africa**	-0.23082	10	-1.03469	10
**Eastern sub-Saharan Africa**	0.104454	10	-1.36997	10
**Northern Africa**	0.22028	10	-1.48579	10
**Southern sub-Saharan Africa**	-0.17385	10	-1.09166	10
**Western sub-Saharan Africa**	0.181774	10	-1.44729	10

5.3.5 Mesh construction

We constructed the finite elements mesh for the stochastic partial differential equation approximation to the Gaussian process regression using a simplified polygon boundary (in which coastlines and complex boundaries were smoothed) for each of the regions within our model. We set the inner mesh triangle maximum edge length (the mesh size for areas over land) to be 0.2 degrees, and the buffer maximum edge length (the mesh size for areas over the ocean) to be 5.0 degrees. An example finite elements mesh constructed for eastern sub-Saharan mesh can be found in Supplementary Figure 12.

5.3.6 Model fitting and estimate generation

Models were fit in INLA with methods consistent with those used in geospatial modelling of under-5 mortality, published previously.[15]

Resampling K-means weights (Supplementary Methods 5.2) were used within the INLA fit by multiplying the corresponding log-likelihood evaluation for the specific observation by the observation's K-means weight. These weights are used to ensure that we do not artificially inflate the amount of information in the dataset by effectively using them to inflate the dispersion in the log-likelihood for resampled-polygon points. While the model this induces is not necessarily generative it does yield a well-defined target distribution. This is analogous to how weighting is often done in generalized additive models.[25] Data points that could be georeferenced to latitude-longitude locations were assigned a weight of 1, ensuring that when the log-likelihood contribution from that observation was evaluated it contributed only to the log-likelihood at the observation's space-time location. For cluster locations generated based on the polygon resampling process, the log-likelihood of those points contributed proportionate to the K-means weights, effectively diffusing the evaluation of the observation across the polygon.

As part of the ensemble modelling process, prediction surfaces from the out-of-sample ensemble sub-models were used as covariates in the spatiotemporal model. Estimates of the fixed effects beta coefficients derived from the contribution of each of the sub-models to INLA's predicted prevalence estimates, in conjunction with parameter estimates of the contribution of location and time (based on estimated parameters described in model description in the Supplementary Methods 5.3.3) were generated and can be found in Supplementary Tables 5-7. To create final estimates, the in-sample prediction surfaces of prevalence from the sub-models (serving as covariates) were used as covariates in conjunction with the fitted random effects from INLA to predict and calculate estimates of prevalence for each pixel in each year.

Our implementation of INLA using the R-INLA software relies on a Gaussian approximation of the full conditional distribution of latent variables, and uses the empirical Bayes approximation for the hyperparemeters.[26] We have tried the full hyperparameter grid integration and CCD integration in various settings and have found our models to be nearly indistinguishable. For the sake of computing resource efficiency (with which we always operate at the margins), we have proceeded with using the empirical Bayes procedure. In a very similar setting with malaria household survey data other authors (including the senior author here) compared the INLA results directly with results from Hamiltonian Markov Chain Monte Carlo and found nearly identical results between the two fits.[27]

All estimates were generated by taking 1,000 draws from the posterior distribution, which yielded 1,000 candidate maps used to summarise the pixel- and aggregated-level statistics. For estimates at the pixel level, these draws were used directly to generate estimates and uncertainty. Aggregated estimates, in which estimates at the pixel level were summarised to administrative boundaries, were generated by creating population-weighted averages for each administrative boundary, for each draw. 95% uncertainty intervals around the mean of our estimates (see Supplementary Fig. 13-15) were generated by taking the 5% and 95% quantiles of each of the draws, at the pixel or administrative level.

doi:10.1038/nature25760

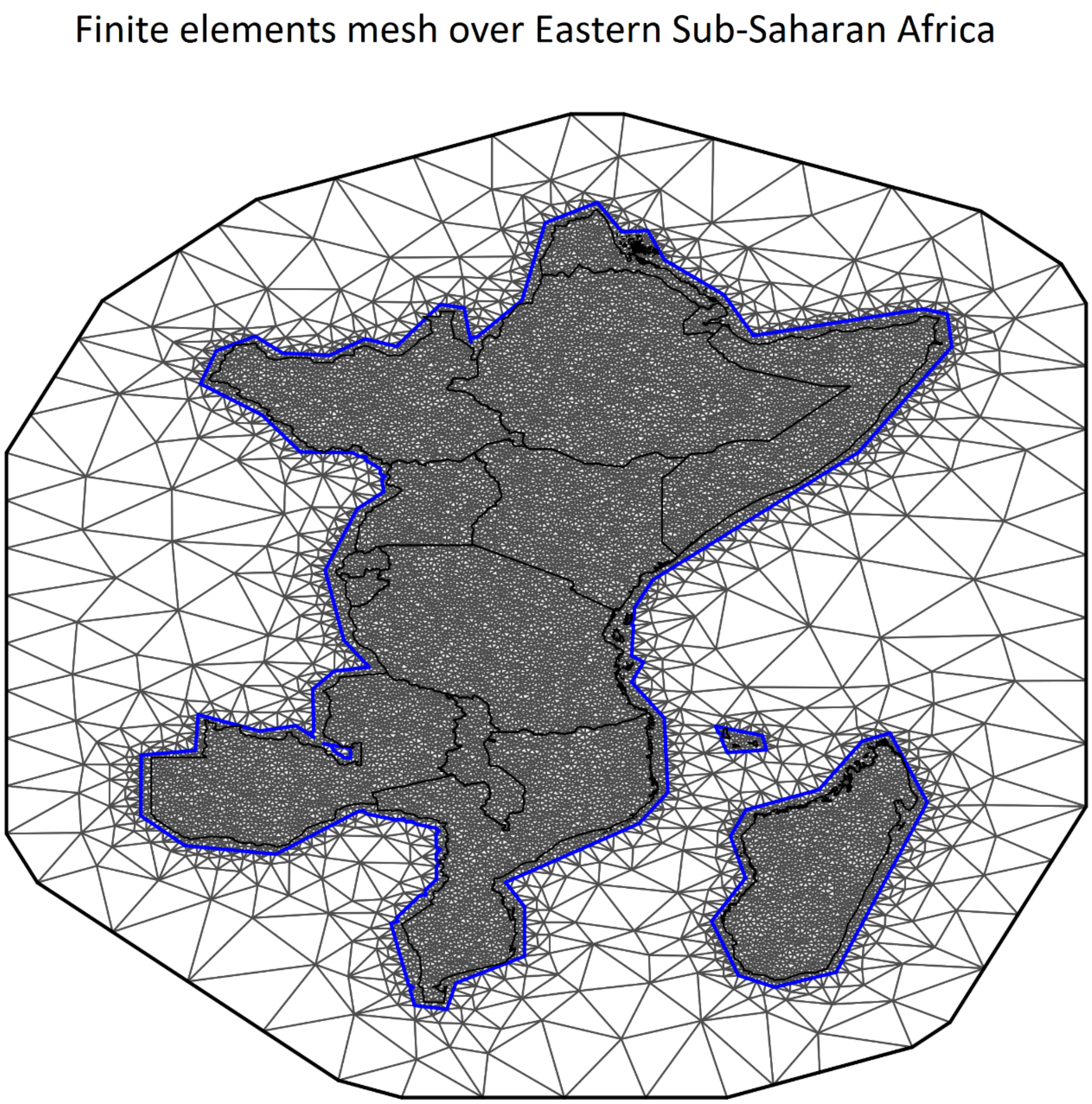

Supplementary Figure 12. Finite elements mesh.
The finite elements mesh used to fit the space-time correlated error for the Eastern sub-Saharan Africa (ESSA) region overlaid on the countries in ESSA. Both the fine-scale mesh over land in the modelling region and the coarser buffer region mesh are shown. The simplified region polygon used to determine the boundary for the modelling region is shown in blue.

5.3.7 Model Results

Fitted parameters and hyperparameters, as well as their 95% uncertainty intervals are shown by indicator and region in Supplementary Tables 5-7. Spatial hyperparameters (τ and κ) and their uncertainties have been transformed into more interpretable nominal variance and range parameters. Nominal variance, approximating the variance at any single point, is calculated as $nom.var = 4\pi\kappa^2\tau^2$, and nominal range, approximating the distance before spatial correlation decays by 90%, as $range = \sqrt{8}/\kappa$.[20]

doi:10.1038/nature25760

Supplementary Table 5. Stunting fitted parameters.

Lower, median, and upper quantiles (0.025%, 0.50%, 0.975%) are displayed for the main parameters from the stunting models by region. The fixed effects covariates corresponding to the predicted ensemble rasters are shown in the first five rows, while fitted values for the spatiotemporal field hyperparameters and the precisions (inverse variance) for our random effects are shown in the bottom five rows.

	Central sub-Saharan Africa quantiles			Eastern sub-Saharan Africa quantiles			Northern Africa quantiles			Southern sub-Saharan Africa quantiles			Western sub-Saharan Africa quantiles		
	**0.025**	**0.500**	**0.975**	**0.025**	**0.500**	**0.975**	**0.025**	**0.500**	**0.975**	**0.025**	**0.500**	**0.975**	**0.025**	**0.500**	**0.975**
**int**	-0.090	-0.042	0.006	-0.065	-0.022	0.021	-0.075	-0.036	0.004	-0.106	-0.039	0.028	-0.064	-0.023	0.017
**gam**	0.295	0.466	0.637	0.205	0.312	0.419	0.150	0.266	0.382	0.268	0.475	0.681	0.186	0.306	0.426
**gbm**	0.407	0.567	0.726	0.547	0.649	0.750	0.715	0.845	0.974	0.507	0.773	1.040	0.265	0.355	0.446
**lasso**	-0.237	-0.032	0.172	-0.082	0.039	0.160	-0.252	-0.111	0.031	-0.501	-0.248	0.005	0.190	0.338	0.487
**Nominal Range**	3.212	4.197	5.585	1.717	1.957	2.226	0.997	1.224	1.492	2.698	4.259	6.836	2.092	2.384	2.685
**Nominal Variance**	0.025	0.035	0.047	0.068	0.081	0.092	0.071	0.085	0.101	0.015	0.026	0.045	0.062	0.071	0.082
**Ar1 ρ**	-0.116	0.152	0.403	0.478	0.580	0.653	-0.030	0.102	0.222	0.120	0.506	0.775	0.123	0.223	0.316
**Precision for IID.ID**	0.014	0.016	0.019	0.068	0.075	0.081	0.025	0.032	0.040	0.019	0.023	0.028	0.024	0.028	0.032

doi:10.1038/nature25760

Supplementary Table 6. Wasting fitted parameters.

Lower, median, and upper quantiles (0.025%, 0.50%, 0.975%) are displayed for the main parameters from the wasting models by region. The fixed effects covariates corresponding to the predicted ensemble rasters are shown in the first five rows, while fitted values for the spatiotemporal field hyperparameters and the precisions (inverse variance) for our random effects are shown in the bottom five rows.

	Central sub-Saharan Africa quantiles			Eastern sub-Saharan Africa quantiles			Northern Africa quantiles			Southern sub-Saharan Africa quantiles			Western sub-Saharan Africa quantiles		
	**0.025**	**0.500**	**0.975**	**0.025**	**0.500**	**0.975**	**0.025**	**0.500**	**0.975**	**0.025**	**0.500**	**0.975**	**0.025**	**0.500**	**0.975**
**int**	-0.112	-0.042	0.027	-0.088	-0.045	-0.003	-0.094	-0.030	0.033	-0.100	-0.052	-0.004	-0.070	-0.012	0.046
**gam**	0.105	0.262	0.419	0.181	0.315	0.448	0.150	0.353	0.555	0.130	0.253	0.375	0.302	0.460	0.617
**gbm**	0.117	0.222	0.328	0.603	0.715	0.827	0.616	0.806	0.995	0.736	0.850	0.964	0.734	0.886	1.037
**lasso**	0.349	0.516	0.682	-0.177	-0.030	0.118	-0.329	-0.158	0.013	-0.238	-0.103	0.032	-0.532	-0.346	-0.159
**Nominal Range**	2.235	2.707	3.245	1.505	1.743	2.021	1.250	3.429	12.522	1.034	1.308	1.637	3.309	5.441	9.142
**Nominal Variance**	0.088	0.106	0.128	0.073	0.084	0.096	0.002	0.011	0.038	0.090	0.110	0.136	0.010	0.019	0.035
**Ar1 ρ**	0.377	0.513	0.630	-0.091	0.021	0.128	-0.782	-0.178	0.521	-0.306	-0.157	0.001	-0.505	-0.011	0.491
**Precision for IID.ID**	0.071	0.082	0.094	0.028	0.034	0.040	0.039	0.054	0.073	0.024	0.031	0.039	0.019	0.023	0.029

46

doi:10.1038/nature25760

Supplementary Table 7. Underweight fitted parameters.
Lower, median, and upper quantiles (0.025%, 0.50%, 0.975%) are displayed for the main parameters from the underweight models by region. The fixed effects covariates corresponding to the predicted ensemble rasters are shown in the first five rows, while fitted values for the spatiotemporal field hyperparameters and the precisions (inverse variance) for our random effects are shown in the bottom five rows.

	Central sub-Saharan Africa quantiles			Eastern sub-Saharan Africa quantiles			Northern Africa quantiles			Southern sub-Saharan Africa quantiles			Western sub-Saharan Africa quantiles		
	**0.025**	**0.500**	**0.975**	**0.025**	**0.500**	**0.975**	**0.025**	**0.500**	**0.975**	**0.025**	**0.500**	**0.975**	**0.025**	**0.500**	**0.975**
**int**	-0.096	-0.037	0.021	-0.076	-0.024	0.027	-0.086	-0.020	0.046	-0.098	-0.031	0.036	-0.089	-0.046	-0.003
**gam**	0.243	0.425	0.608	0.268	0.390	0.513	0.325	0.446	0.567	0.191	0.398	0.606	0.098	0.213	0.329
**gbm**	0.544	0.717	0.889	0.400	0.500	0.599	0.304	0.429	0.554	0.351	0.569	0.786	0.402	0.496	0.591
**lasso**	-0.377	-0.142	0.093	-0.021	0.110	0.241	-0.040	0.125	0.291	-0.177	0.033	0.242	0.163	0.290	0.418
**Nominal Range**	3.399	4.662	6.400	2.018	2.337	2.759	1.859	2.354	2.991	2.545	4.442	7.937	1.806	2.022	2.284
**Nominal Variance**	0.022	0.032	0.048	0.071	0.083	0.098	0.092	0.118	0.151	0.009	0.021	0.042	0.073	0.082	0.097
**Ar1 ρ**	0.065	0.356	0.600	0.529	0.625	0.706	-0.261	-0.086	0.100	-0.163	0.348	0.714	0.267	0.360	0.448
**Precision for IID.ID**	0.014	0.017	0.020	0.068	0.075	0.083	0.016	0.020	0.024	0.023	0.029	0.036	0.030	0.034	0.039

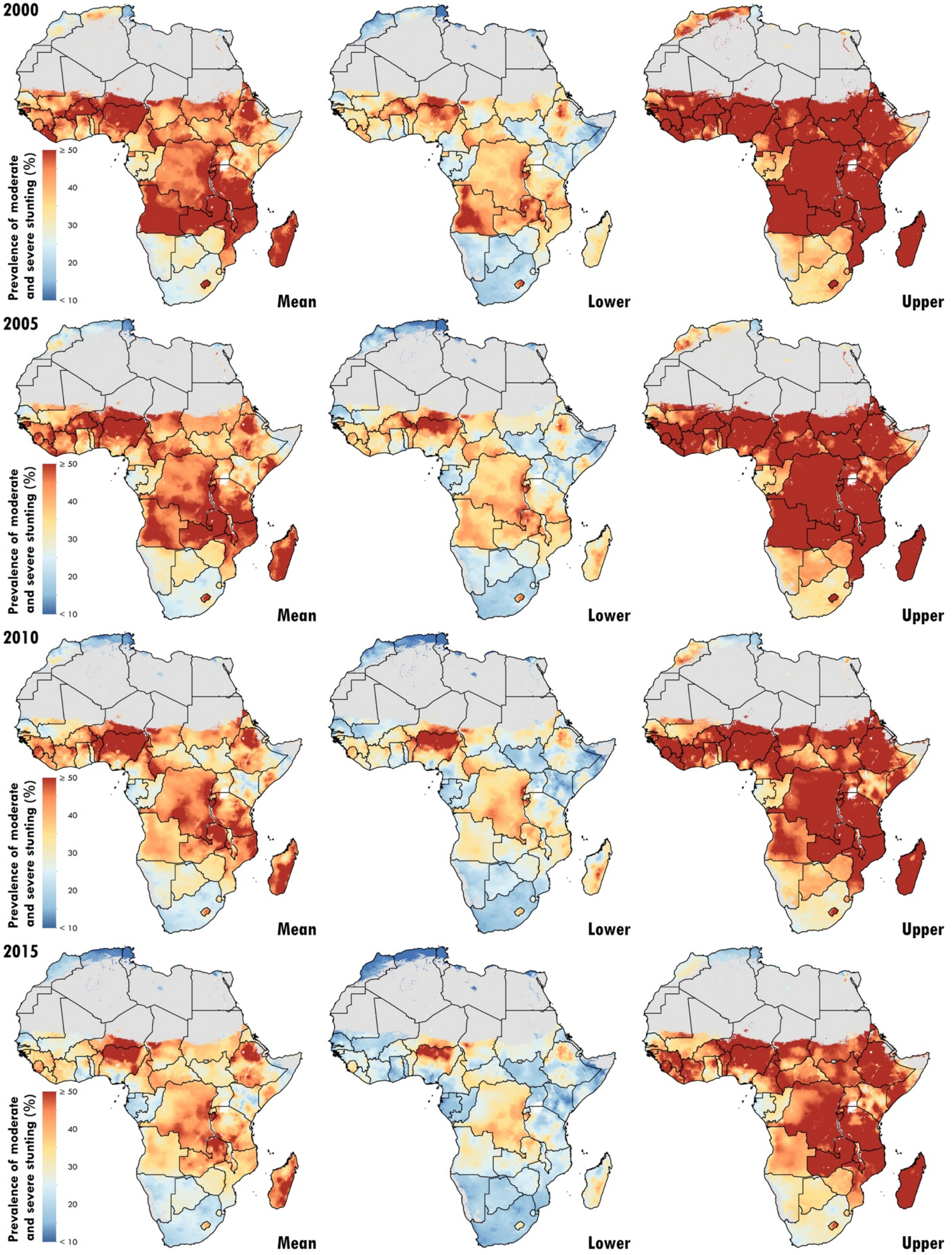

Supplementary Figure 13. Stunting posterior means and 95% uncertainty intervals.

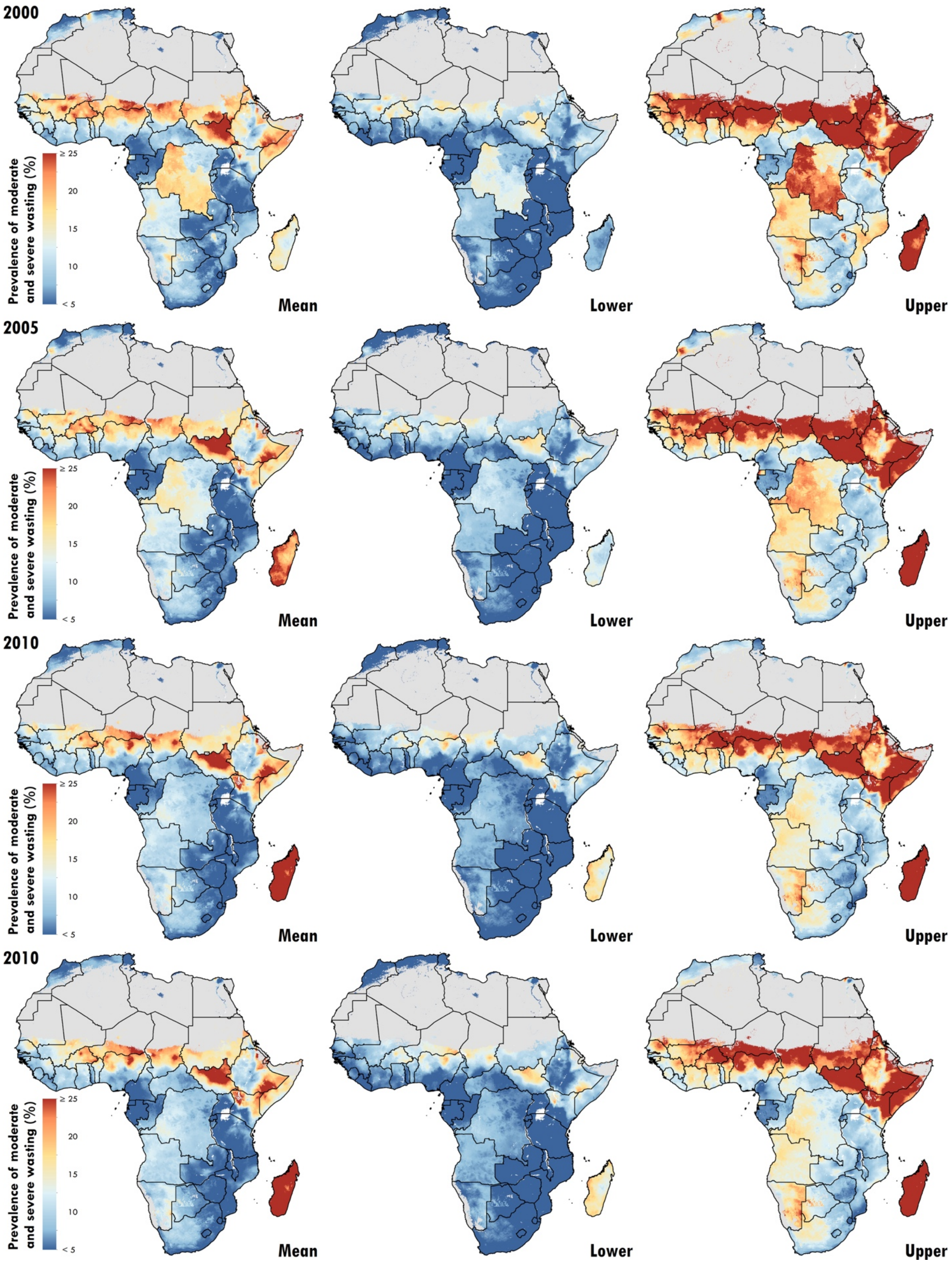

Supplementary Figure 14. Wasting posterior means and 95% uncertainty intervals.

doi:10.1038/nature25760

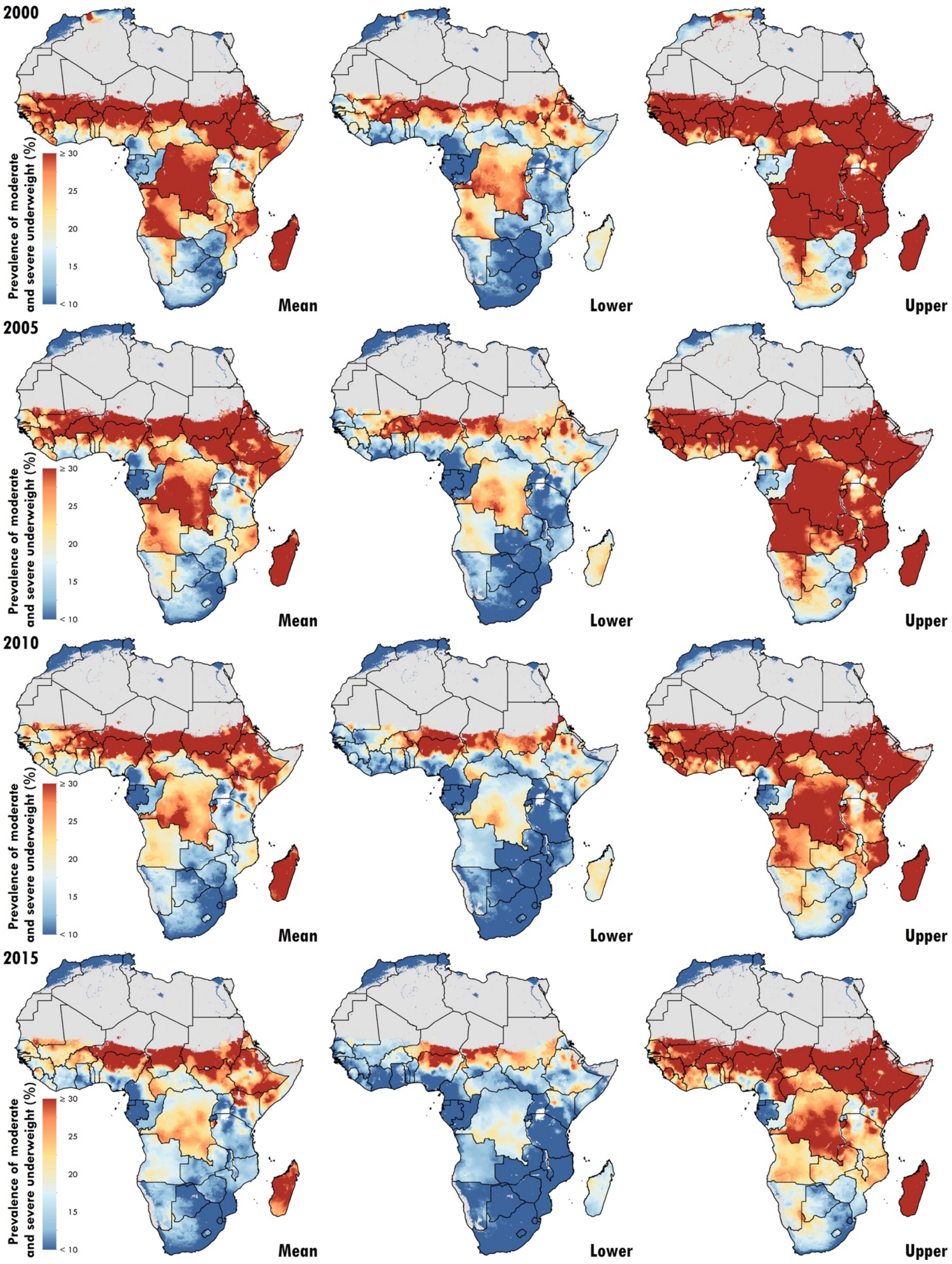

Supplementary Figure 15. Underweight posterior means and 95% uncertainty intervals.

5.4 Model validation

5.4.1 In-sample metrics

For each indicator, we generated a suite of diagnostic plots for each region and country estimated, in order to assess the in-sample performance of our model and compare to national-level estimates produced by GBD.

To explore residual error over space and time, absolute error (data minus predicted posterior mean estimates at the corresponding pixels) were produced at five-year intervals (2000, 2005, 2010, and 2015) for each modelled region (see Supplementary Fig. 25-27).

5.4.2 Metrics of predictive validity

In order to assess the predictive validity of our estimates, we validated our models using spatially stratified five-fold out-of-sample cross-validation.[28] To construct each spatial fold, we used a modified bi-tree algorithm to spatially aggregate data points. This algorithm recursively partitions two-dimensional space, alternating between horizontal and vertical splits on the weighted data sample size medians, until the data contained within each spatial partition are of a similar sample size. The depth of recursive partitioning is constrained by the target sample size within a partition and the minimum number of clusters or pseudo-clusters allowed within each spatial partition (in this case, a minimum sample size of 500 was used). These spatial partitions are then allocated to one of five folds for cross-validation. For validation, each geostatistical model was run five times, each time holding out data from one of the folds, generating a set of out-of-sample predictions for the held-out data. For each indicator, a full suite of out-of-sample predictions over the entire dataset was generated by combing the out-of-sample predictions from the five cross-validation runs.

Using these out-of-sample predictions, we then calculated mean error (ME, or bias), root-mean-squared-error (RMSE, which summarises total variance), coefficient of variation (CoV, defined to be the standard deviation divided by the mean and multiplied by 100, which is a measure of relative variability), and 95% coverage of our predictive intervals (the proportion of observed out-of-sample data that fall within our predicted 95% credible intervals) aggregated up to different administrative levels (levels 0, 1, and 2) as defined by FAO Global Administrative Unit Layers (GAUL).[29] Administrative level 0 borders correspond to national boundaries, administrative level 1 borders generally correspond to regions, provinces, or state-level boundaries within a country, and administrative level 2 borders correspond to the next finer subdivision, often districts, within regions. These metrics are summarised in Supplementary Tables 4-12 for each indicator and are calculated across all regions. Included in the sample tables for comparison are the same metrics calculated on in-sample predictions.

5.4.3 Model comparison

To demonstrate the utility if the stacking ensemble, for each indicator we ran five, five-fold cross-validation holdout experiments using different combinations of covariates and random effects. The following five models were compared:

1) Raw covariates:
$$\mathrm{logit}(p_i) = \beta_0 + X_i\beta_{raw} + \epsilon_i$$

2) Stacking predictions as covariates:
$$\mathrm{logit}(p_i) = \beta_0 + X_i\beta_{stack} + \epsilon_i$$

3) Gaussian Process (GP):
$$\mathrm{logit}(p_i) = \beta_0 + \epsilon_{GP_i} + \epsilon_i$$

4) Raw covariates + GP:
$$\mathrm{logit}(p_i) = \beta_0 + X_i\beta_{raw} + \epsilon_{GP_i} + \epsilon_i$$

5) Stacking covariates + GP:
$$\mathrm{logit}(p_i) = \beta_0 + X_i\beta_{stack} + \epsilon_{GP_i} + \epsilon_i$$

For stunting, wasting, and underweight we have aggregated our out-of-sample results (RMSE, coverage, and bias) from the cross-validation to the spatial resolution used to generate the 5-folds (i.e. to quad-tree leafs) and compared these numbers to the raw prevalence data observations which have been aggregated in the same way. By doing this at the draw level from each of the 5 experiments we can compare out-of-sample bias, root-mean-square error, and 95% predictive coverage. Across regions and years, we see that using the stacking ensemble in conjunction with the Gaussian process tends to improve our out-of-sample predictive metrics across the board. These metrics are plotted for comparison in Supplementary Fig. 16-18.

doi:10.1038/nature25760

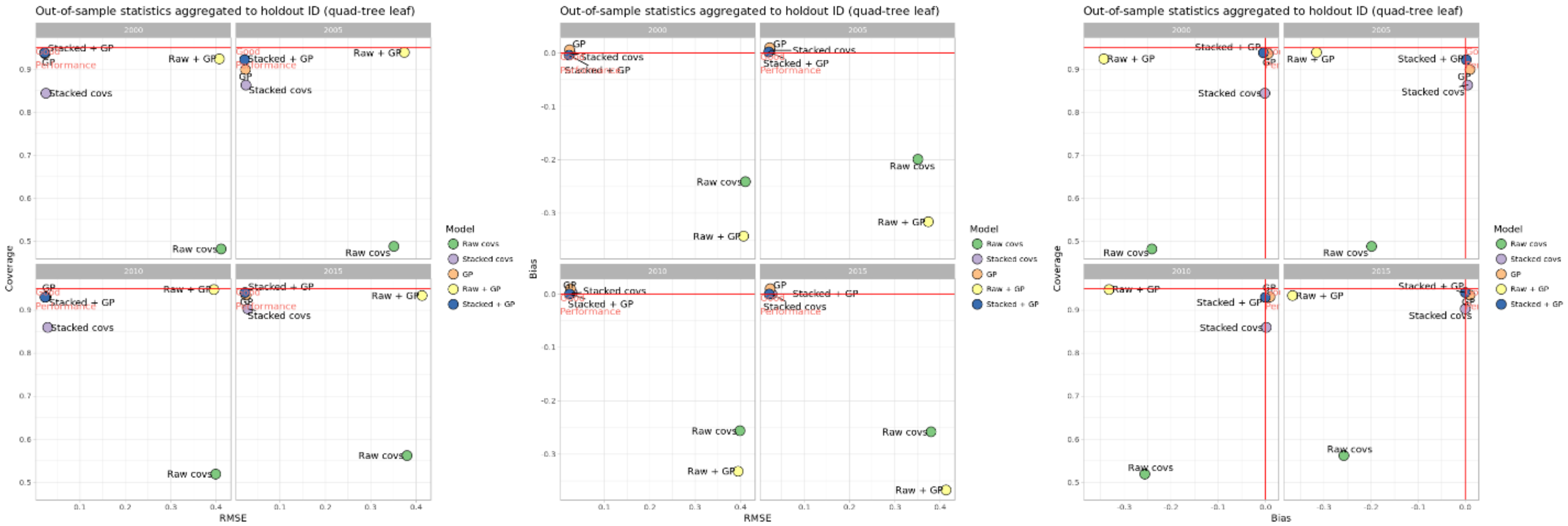

Supplementary Figure 16. Stunting out-of-sample model comparisons.

Fit statistics are aggregated by periods 2000-2002, 2003-2007, 2008-2012, and 2013-2015. “Raw Covs” is the INLA model fit with linear terms on all raw satellite covariates; “Stacked covs” corresponds to an INLA fit with all stacking ensemble prediction surfaces; “GP” is fit only with the space-time Gaussian process; “Raw + GP” is fit with linear terms on all raw covariates and the space-time Gaussian process; “Stacked + GP” is fit with all stacking surfaces and the space-time Gaussian process. Red lines and text draw your attention to the region of the plot that indicates best performance (low bias, low RMSE, and 95% coverage).

doi:10.1038/nature25760

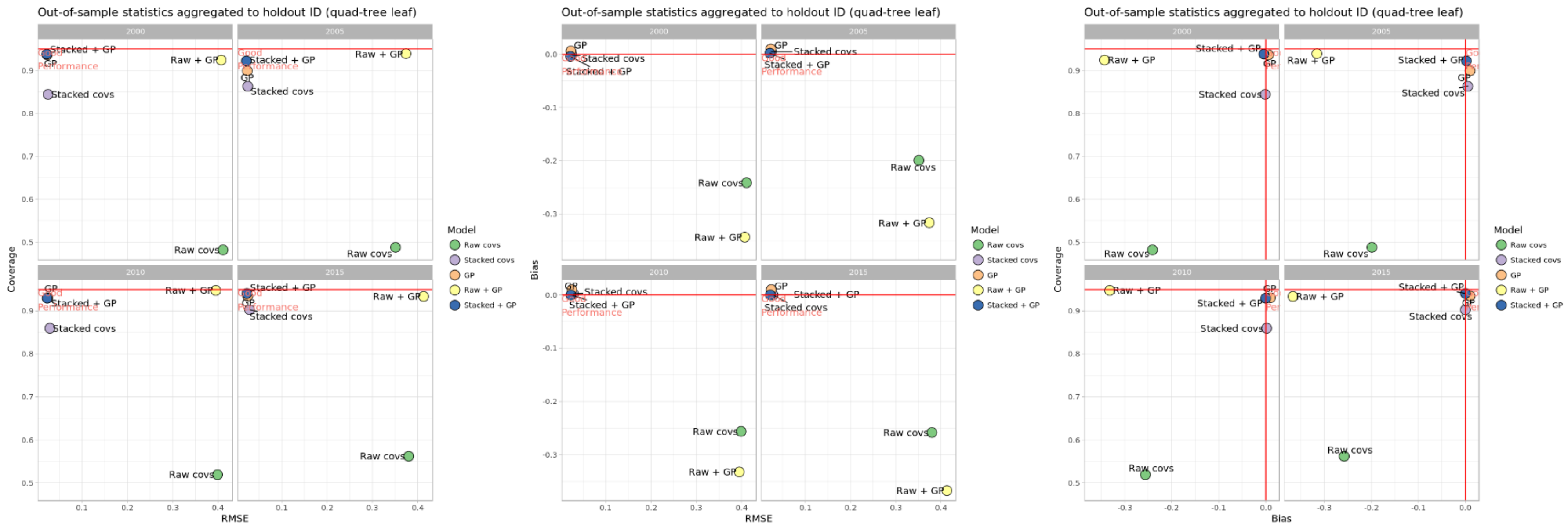

Supplementary Figure 17. Wasting out-of-sample model comparisons.

Fit statistics are aggregated by periods 2000-2002, 2003-2007, 2008-2012, and 2013-2015. “Raw Covs” is the INLA model fit with linear terms on all raw satellite covariates; “Stacked covs” corresponds to an INLA fit with all stacking ensemble prediction surfaces; “GP” is fit only with the space-time Gaussian process; “Raw + GP” is fit with linear terms on all raw covariates and the space-time Gaussian process; “Stacked + GP” is fit with all stacking surfaces and the space-time Gaussian process. Red lines and text draw your attention to the region of the plot that indicates best performance (low bias, low RMSE, and 95% coverage).

doi:10.1038/nature25760

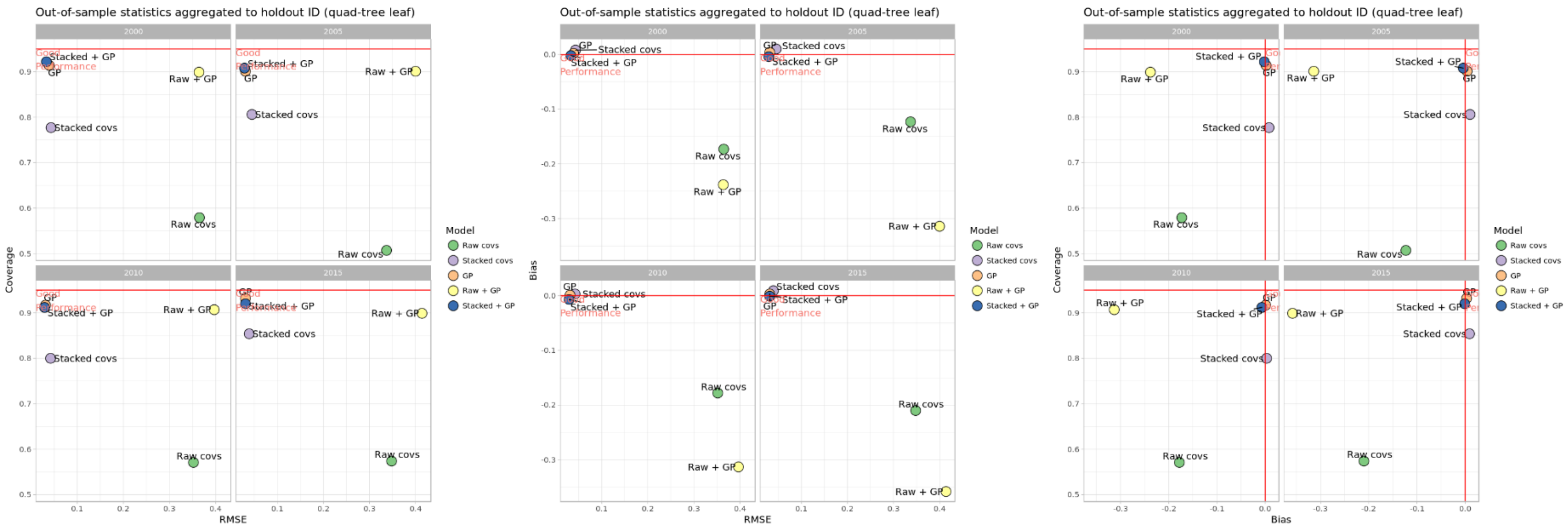

Supplementary Figure 18. Underweight out-of-sample model comparisons.

Fit statistics are aggregated by periods 2000-2002, 2003-2007, 2008-2012, and 2013-2015. “Raw Covs” is the INLA model fit with linear terms on all raw satellite covariates; “Stacked covs” corresponds to an INLA fit with all stacking ensemble prediction surfaces; “GP” is fit only with the space-time Gaussian process; “Raw + GP” is fit with linear terms on all raw covariates and the space-time Gaussian process; “Stacked + GP” is fit with all stacking surfaces and the space-time Gaussian process. Red lines and text draw your attention to the region of the plot that indicates best performance (low bias, low RMSE, and 95% coverage).

5.4.4 Stunting validation metrics

Supplementary Table 8. Predictive metrics for stunting aggregated to admin 0.
The out-of-sample (OOS) column indicates whether the metric was calculated using in-sample or out-of-sample predictions.

Year	OOS	Median SS	Mean err.	RMSE	CoV (%)	Corr.	95% Cov.
**2000**	FALSE	4325.853	-0.004	0.012	2.782	0.992	0.973
**2005**	FALSE	4707.000	0.000	0.014	3.517	0.984	0.947
**2010**	FALSE	6406.000	-0.005	0.013	3.791	0.989	0.943
**2015**	FALSE	5597.084	-0.013	0.021	6.121	0.978	0.949

Supplementary Table 9. Predictive metrics for stunting aggregated to admin 1.

Year	OOS	Median SS	Mean err.	RMSE	CoV (%)	Corr.	95% Cov.
**2000**	FALSE	211.609	-0.004	0.032	7.538	0.963	0.972
**2005**	FALSE	308.102	0.000	0.031	7.618	0.961	0.948
**2010**	FALSE	337.062	-0.005	0.034	9.597	0.957	0.943
**2015**	FALSE	377.000	-0.013	0.032	9.512	0.963	0.949

Supplementary Table 10. Predictive metrics for stunting aggregated to admin 2.

Year	OOS	Median SS	Mean err.	RMSE	CoV (%)	Corr.	95% Cov.
**2000**	FALSE	27.693	-0.004	0.048	11.386	0.922	0.972
**2005**	FALSE	33.059	0.000	0.052	12.852	0.904	0.947
**2010**	FALSE	41.054	-0.005	0.059	16.671	0.892	0.943
**2015**	FALSE	36.000	-0.013	0.066	19.709	0.859	0.948

Supplementary Table 11. Predictive metrics for stunting aggregated to holdout units.

Year	OOS	Median SS	Mean err.	RMSE	CoV (%)	Corr.	95% Cov.
**2000**	FALSE	555.000	-0.004	0.021	4.962	0.979	0.973
**2005**	FALSE	677.671	0.000	0.025	6.063	0.968	0.947
**2010**	FALSE	713.475	-0.005	0.025	7.090	0.967	0.943
**2015**	FALSE	803.370	-0.013	0.026	7.575	0.970	0.945
**2000**	TRUE	555.000	0.000	0.041	9.696	0.914	0.941
**2005**	TRUE	677.671	0.002	0.036	9.014	0.929	0.932
**2010**	TRUE	713.475	-0.007	0.041	11.585	0.906	0.915
**2015**	TRUE	803.370	-0.012	0.034	10.043	0.935	0.938

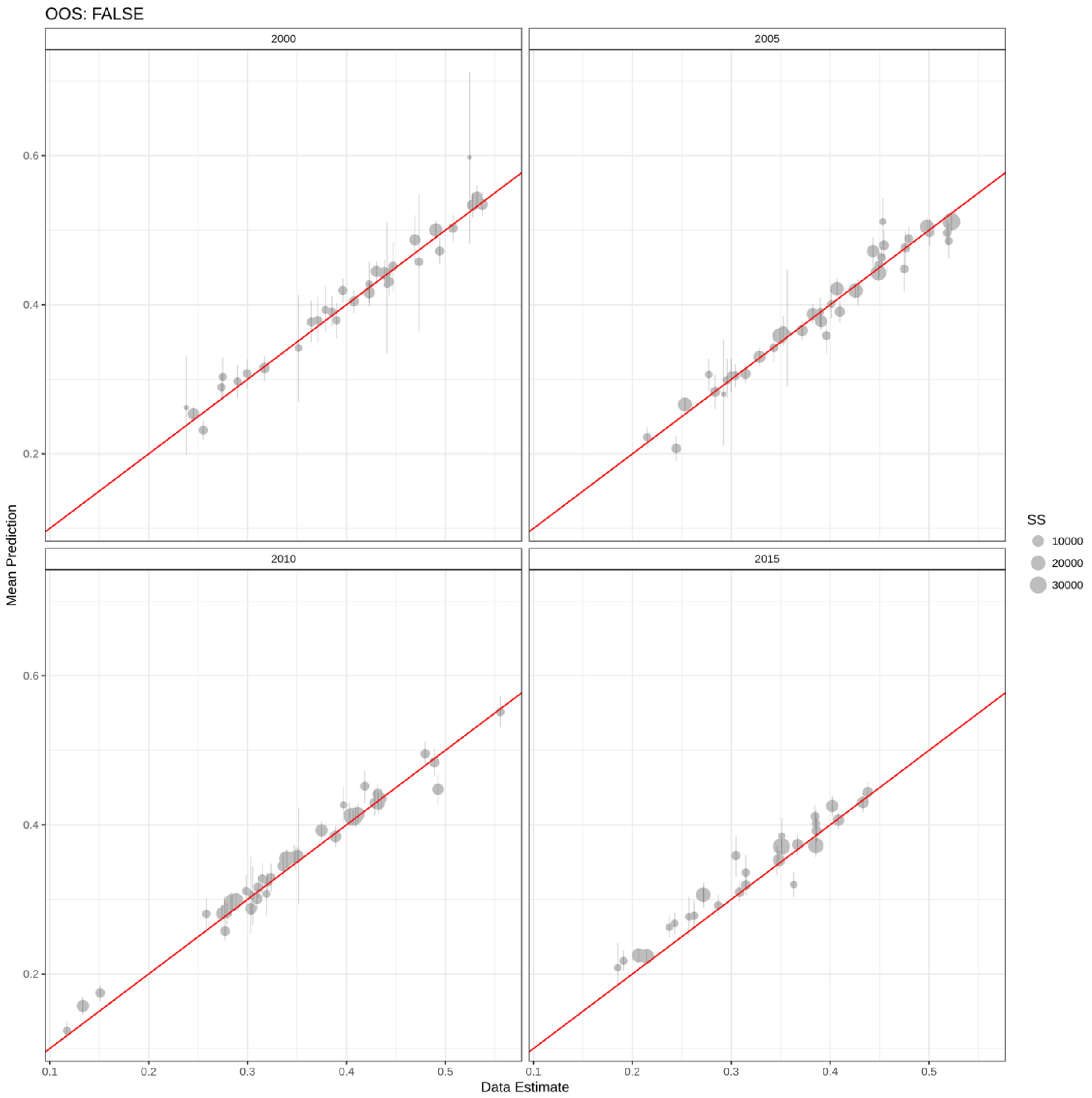

Supplementary Figure 19. Stunting admin 0 aggregation in-sample.
Comparison of in-sample stunting predictions aggregated to admin 0 with 95% uncertainty intervals plotted against admin 0 aggregated data observations.

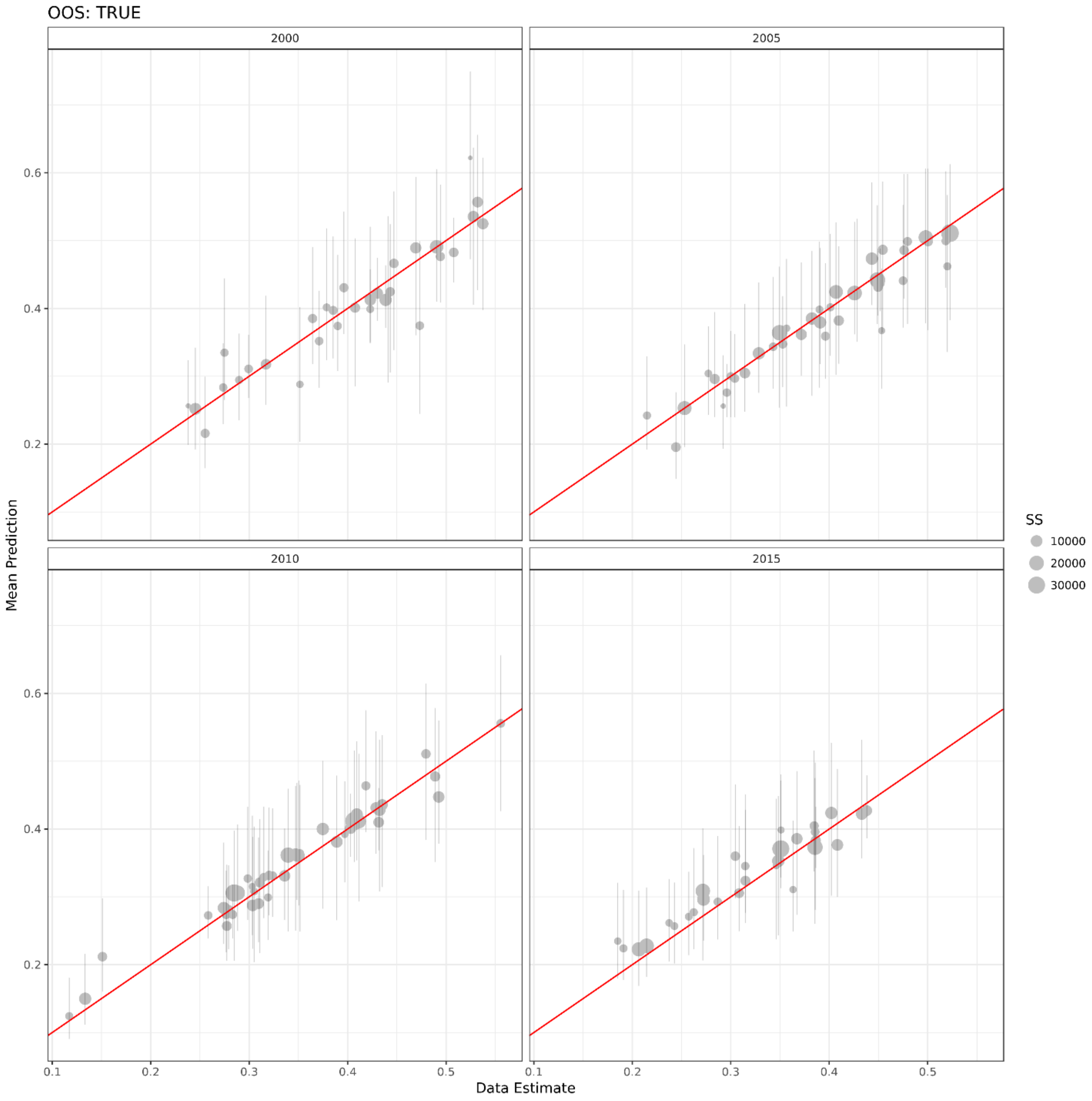

Supplementary Figure 20. Stunting admin 0 aggregation out-of-sample.
Comparison of out-of-sample stunting predictions aggregated to admin 0 with 95% uncertainty intervals plotted against admin 0 aggregated data observations.

doi:10.1038/nature25760

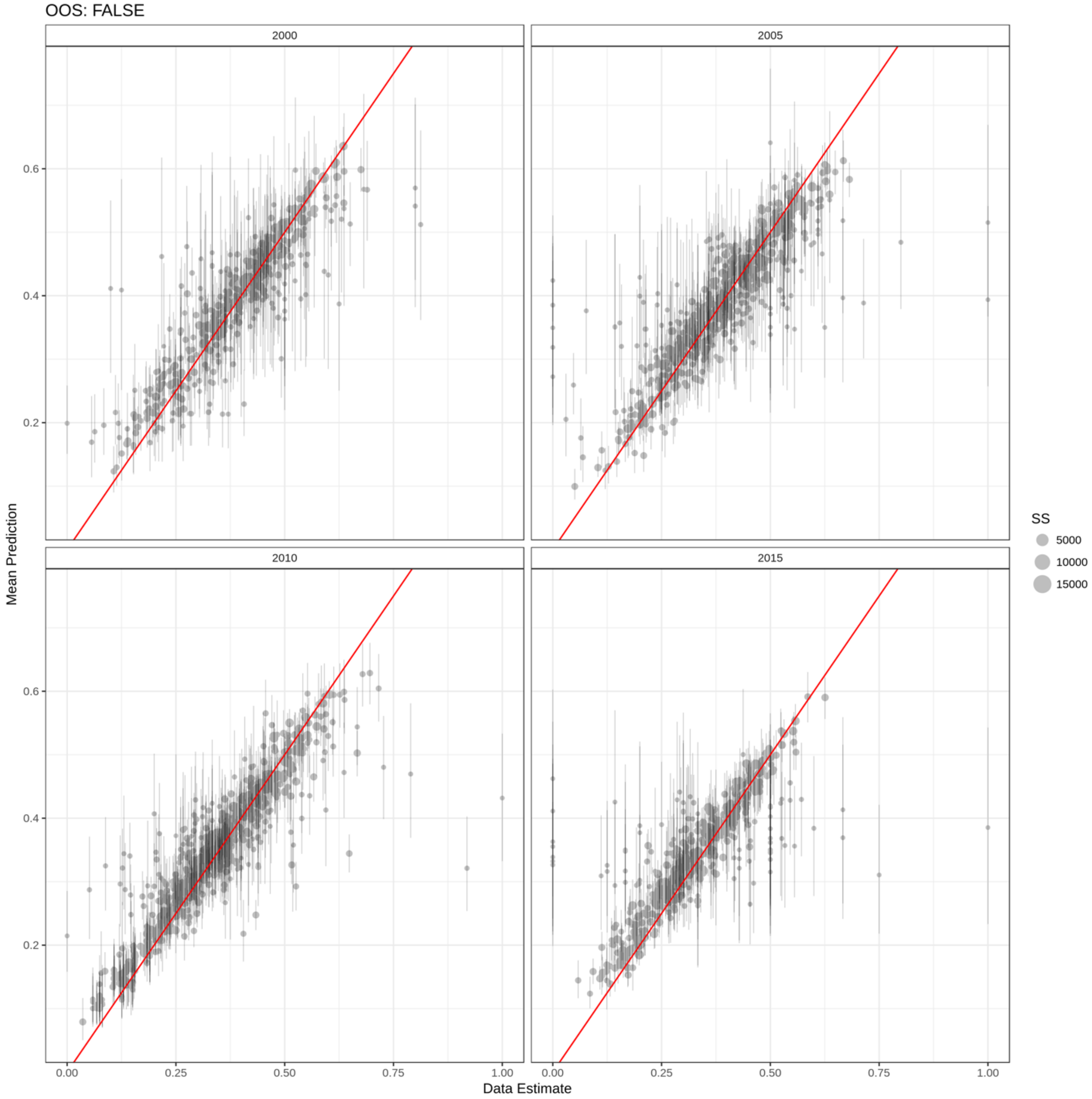

Supplementary Figure 21. Stunting admin 1 aggregation in-sample.
Comparison of in-sample stunting predictions aggregated to admin 1 with 95% uncertainty intervals plotted against admin 1 aggregated data observations.

doi:10.1038/nature25760

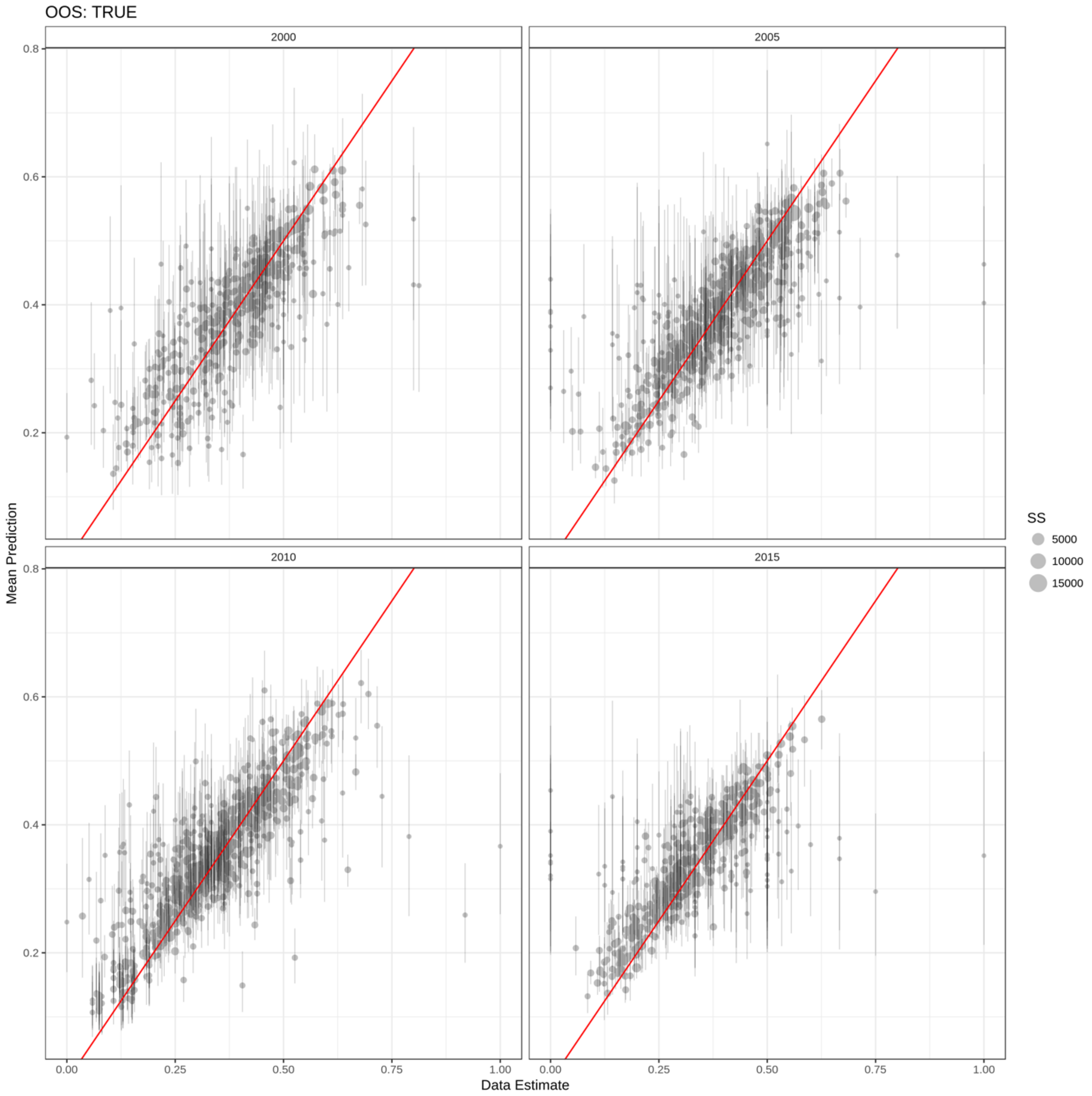

Supplementary Figure 22. Stunting admin 1 aggregation out-of-sample.
Comparison of out-of-sample stunting predictions aggregated to admin 1 with 95% uncertainty intervals plotted against admin 1 aggregated data observations.

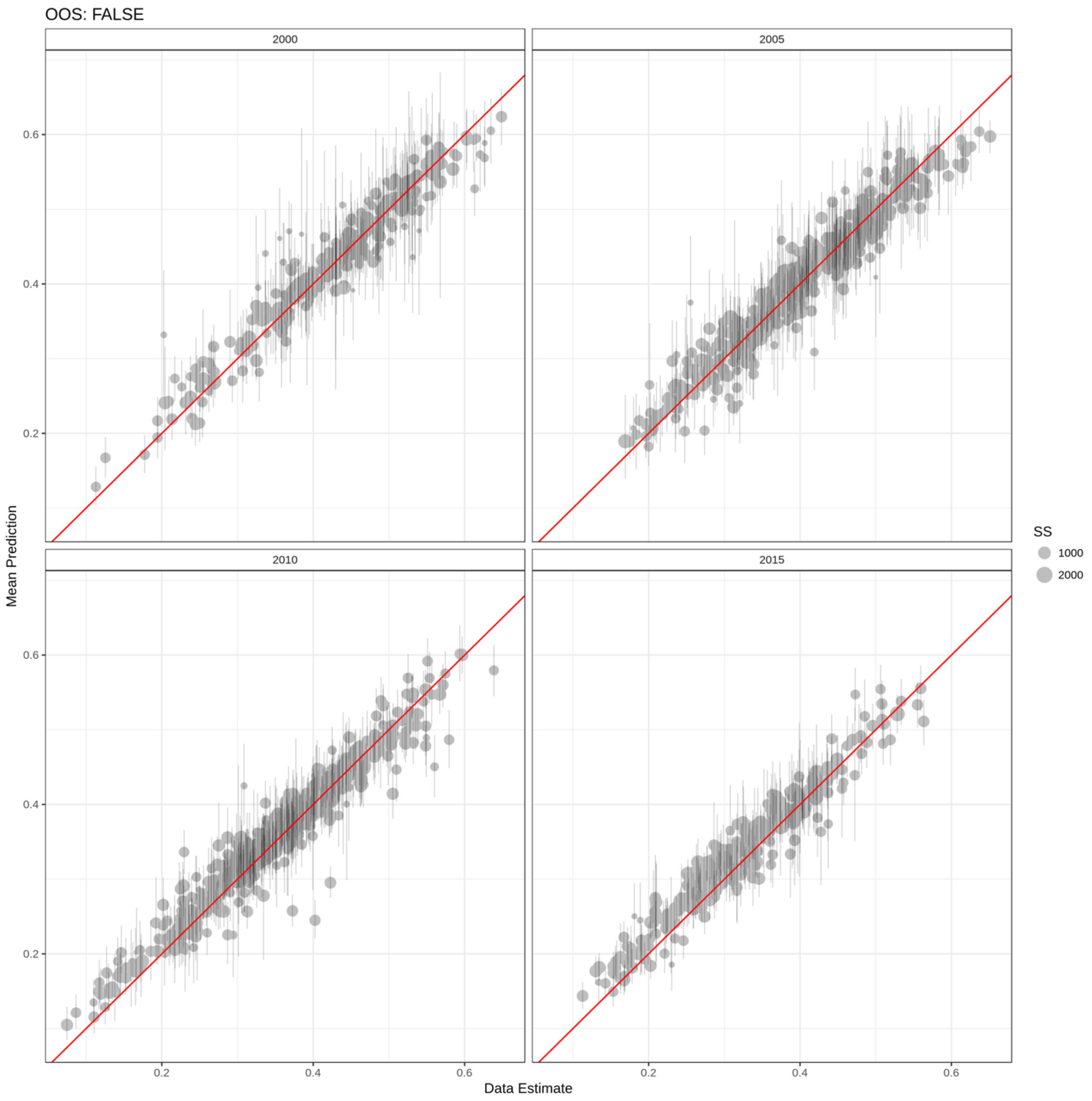

Supplementary Figure 23. Stunting holdout units aggregation in-sample. Comparison of in-sample stunting predictions aggregated to holdout units generated from recursive quad-tree with 95% uncertainty intervals plotted against aggregated data observations from the same spatial region.

doi:10.1038/nature25760

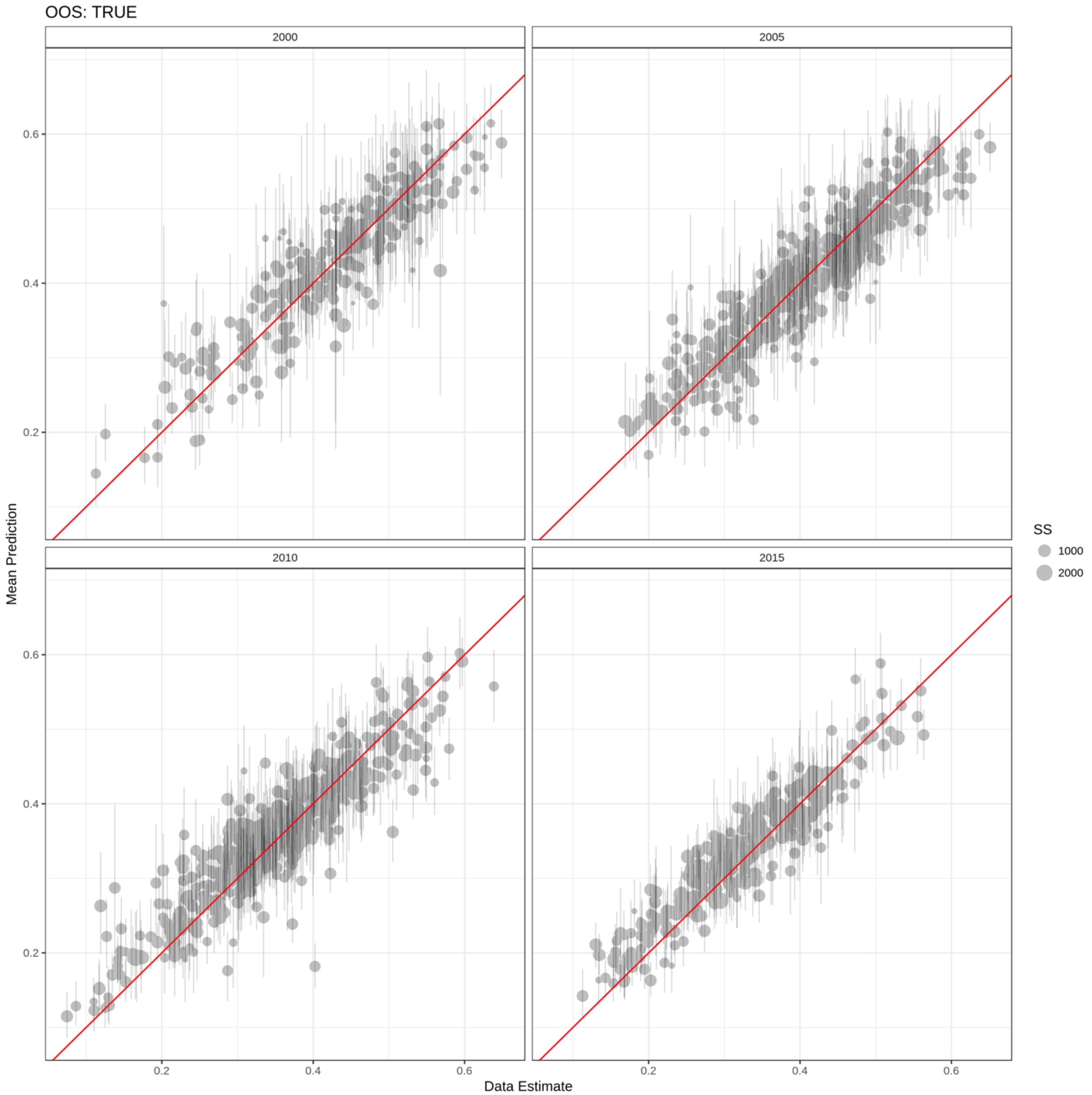

Supplementary Figure 24. Stunting holdout units aggregation out-of-sample. Comparison of out-of-sample predictions aggregated to holdout units generated from recursive quad-tree with 95% uncertainty intervals plotted against aggregated data observations from the same spatial region.

5.4.5 Wasting validation metrics

Supplementary Table 12. Predictive metrics for wasting aggregated to admin 0.
The out-of-sample (OOS) column indicates whether the metric was calculated using in-sample or out-of-sample predictions.

Year	OOS	Median SS	Mean err.	RMSE	CoV (%)	Corr.	95% Cov.
2000	FALSE	4335.433	-0.005	0.013	11.698	0.976	0.969
2005	FALSE	4050.000	-0.001	0.011	11.698	0.977	0.940
2010	FALSE	6412.000	-0.002	0.011	10.558	0.981	0.955
2015	FALSE	5223.361	-0.001	0.006	6.908	0.992	0.962

Supplementary Table 13. Predictive metrics for wasting aggregated to admin 1.

Year	OOS	Median SS	Mean err.	RMSE	CoV (%)	Corr.	95% Cov.
2000	FALSE	208.500	-0.005	0.022	20.397	0.924	0.969
2005	FALSE	300.517	-0.001	0.020	20.645	0.945	0.941
2010	FALSE	347.000	-0.002	0.020	19.644	0.953	0.956
2015	FALSE	368.500	-0.001	0.021	25.380	0.945	0.962

Supplementary Table 14. Predictive metrics for wasting aggregated to admin 2.

Year	OOS	Median SS	Mean err.	RMSE	CoV (%)	Corr.	95% Cov.
2000	FALSE	27.269	-0.005	0.029	26.885	0.879	0.969
2005	FALSE	32.833	-0.001	0.030	30.953	0.891	0.943
2010	FALSE	41.354	-0.002	0.034	34.088	0.881	0.955
2015	FALSE	35.000	-0.001	0.043	50.796	0.831	0.962

Supplementary Table 15. Predictive metrics for wasting aggregated to holdout units.

Year	OOS	Median SS	Mean err.	RMSE	CoV (%)	Corr.	95% Cov.
2000	FALSE	605.000	-0.005	0.015	14.015	0.950	0.970
2005	FALSE	671.144	-0.001	0.016	15.909	0.958	0.943
2010	FALSE	689.981	-0.002	0.015	15.558	0.961	0.955
2015	FALSE	809.000	-0.001	0.017	20.148	0.939	0.963
2000	TRUE	605.000	-0.006	0.023	21.513	0.873	0.943
2005	TRUE	671.144	-0.001	0.022	22.071	0.916	0.930
2010	TRUE	689.981	-0.003	0.023	22.827	0.912	0.938
2015	TRUE	809.000	-0.001	0.023	27.489	0.877	0.950

doi:10.1038/nature25760

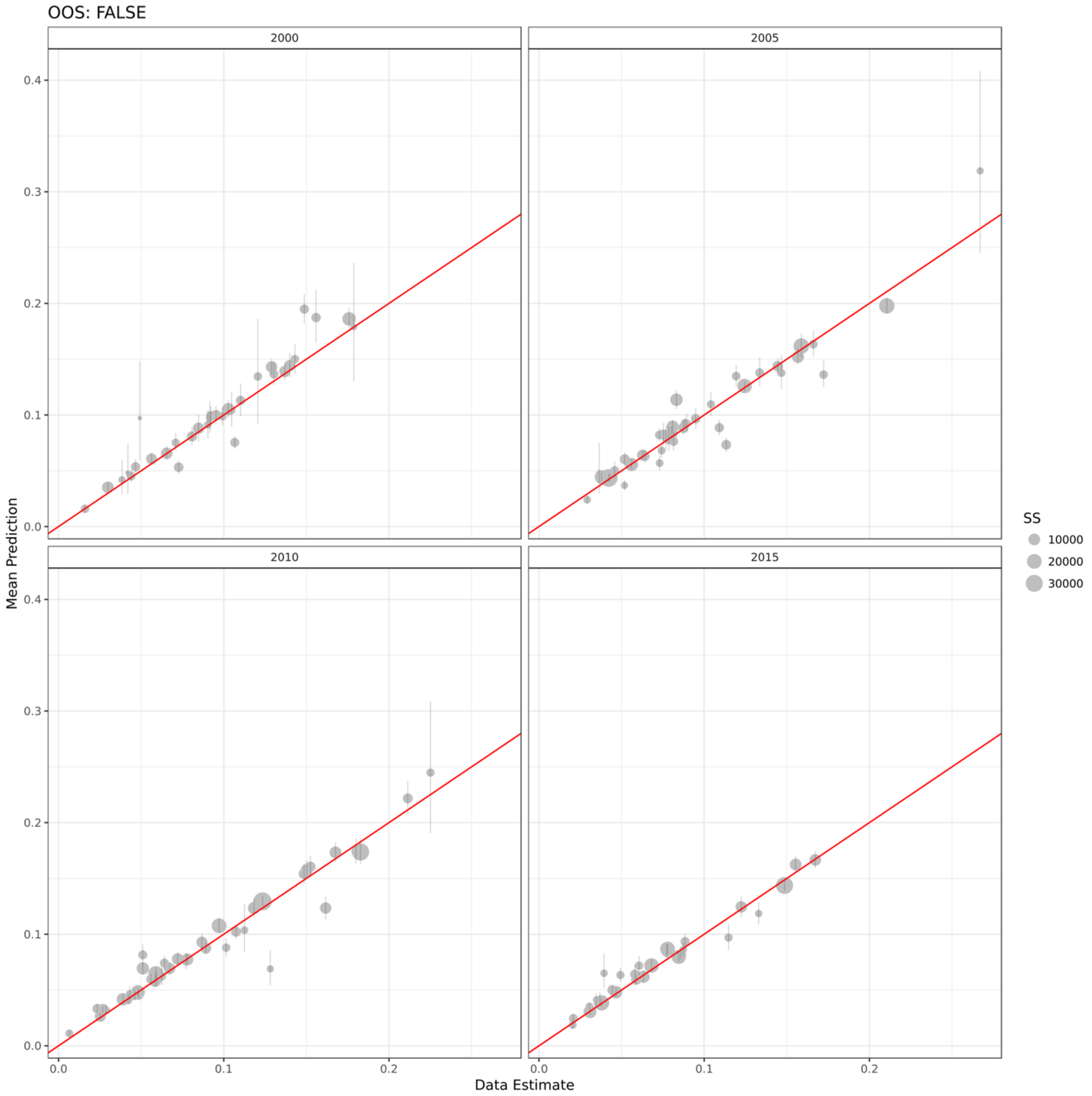

Supplementary Figure 25. Wasting admin 0 aggregation in-sample.
Comparison of in-sample wasting predictions aggregated to admin 0 with 95% uncertainty intervals plotted against admin 0 aggregated data observations.

doi:10.1038/nature25760

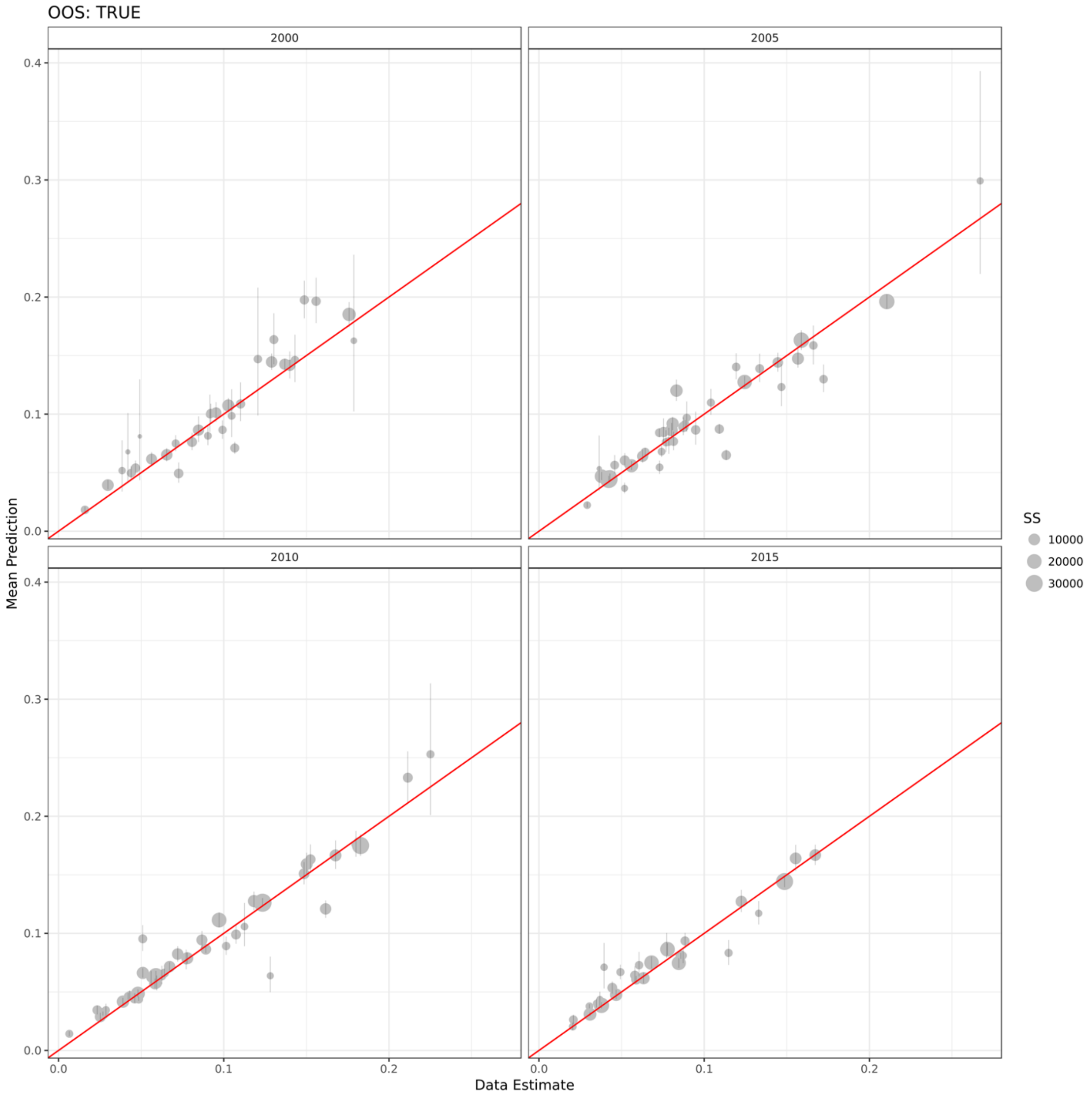

Supplementary Figure 26. Wasting admin 0 aggregation out-of-sample.
Comparison of out-of-sample wasting predictions aggregated to admin 0 with 95% uncertainty intervals plotted against admin 0 aggregated data observations.

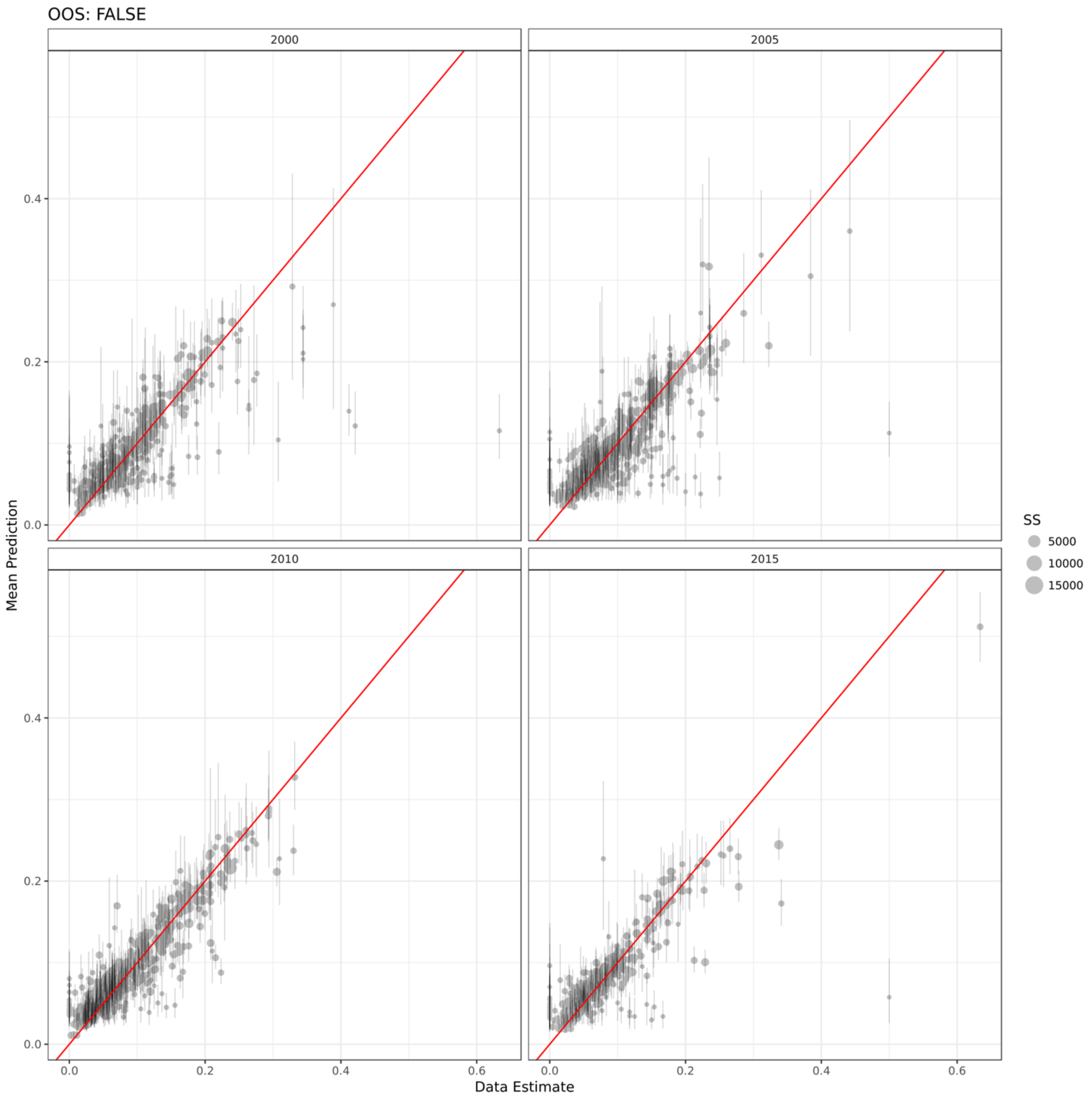

Supplementary Figure 27. Wasting admin 1 aggregation in-sample.
Comparison of in-sample wasting predictions aggregated to admin 1 with 95% uncertainty intervals plotted against admin 1 aggregated data observations.

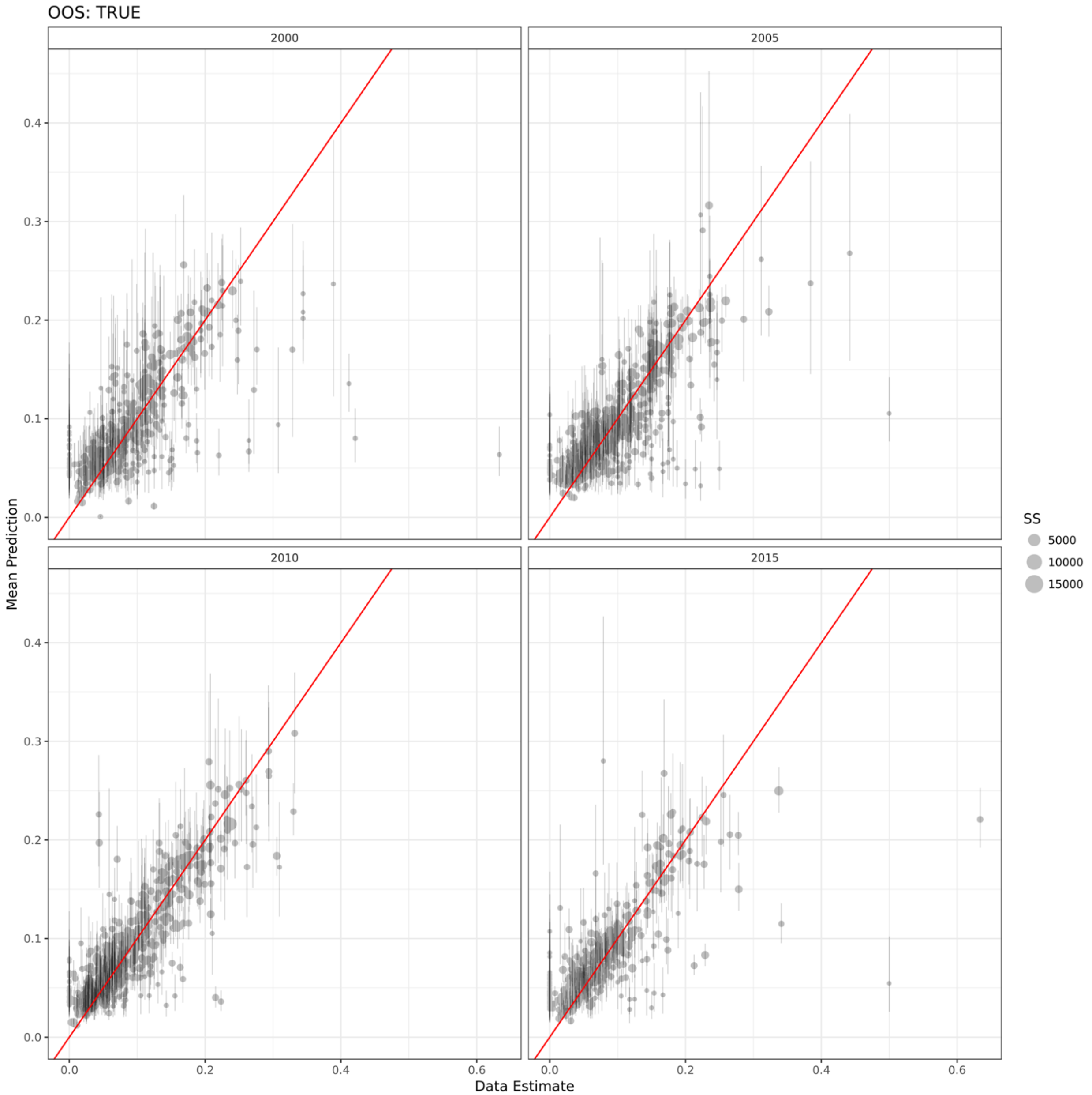

Supplementary Figure 28. Wasting admin 1 aggregation out-of-sample.
Comparison of out-of-sample wasting predictions aggregated to admin 1 with 95% uncertainty intervals plotted against admin 1 aggregated data observations.

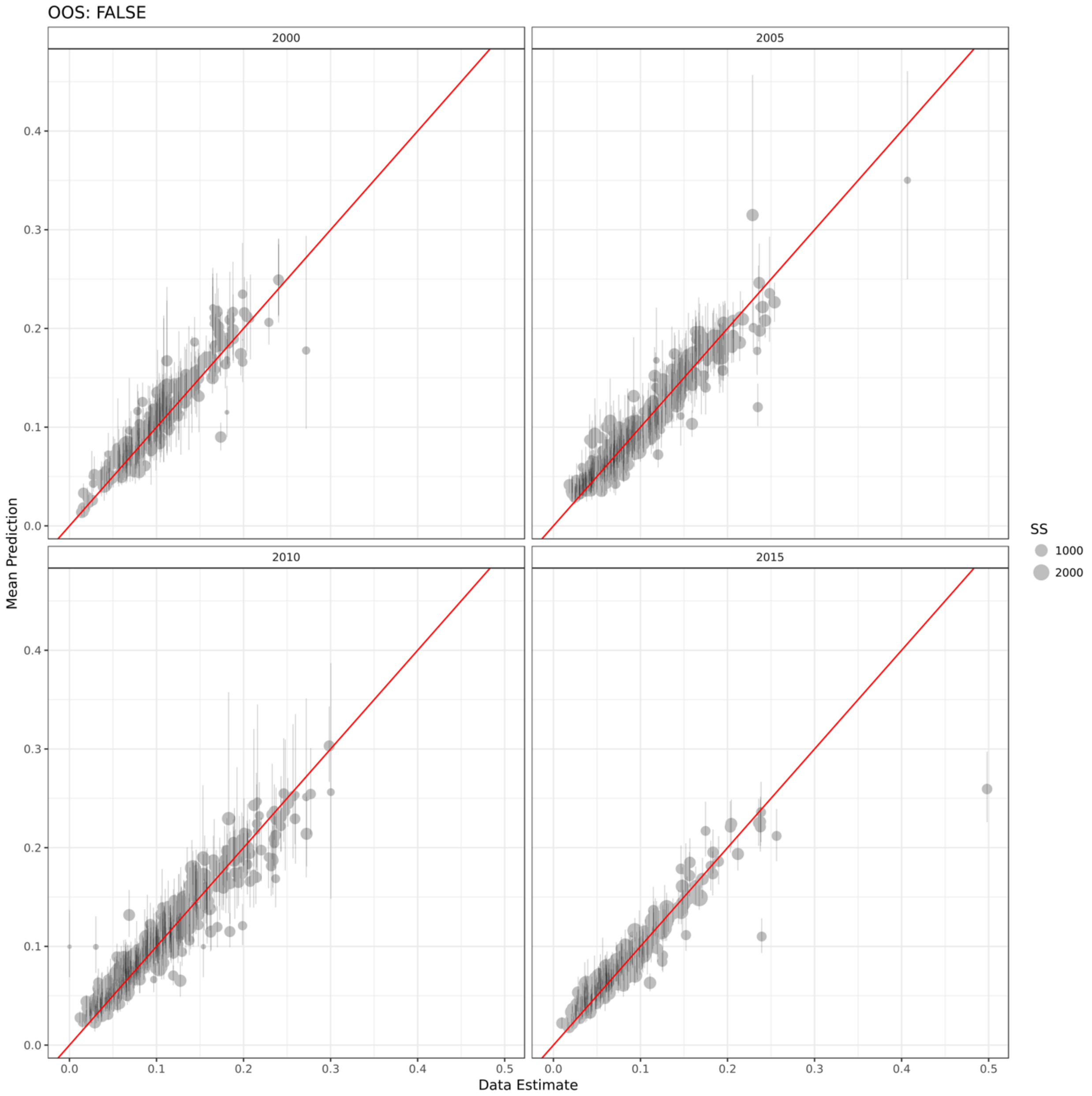

Supplementary Figure 29. Wasting holdout units aggregation in-sample.
Comparison of in-sample wasting predictions aggregated to holdout units generated from recursive quad-tree with 95% uncertainty intervals plotted against aggregated data observations from the same spatial region.

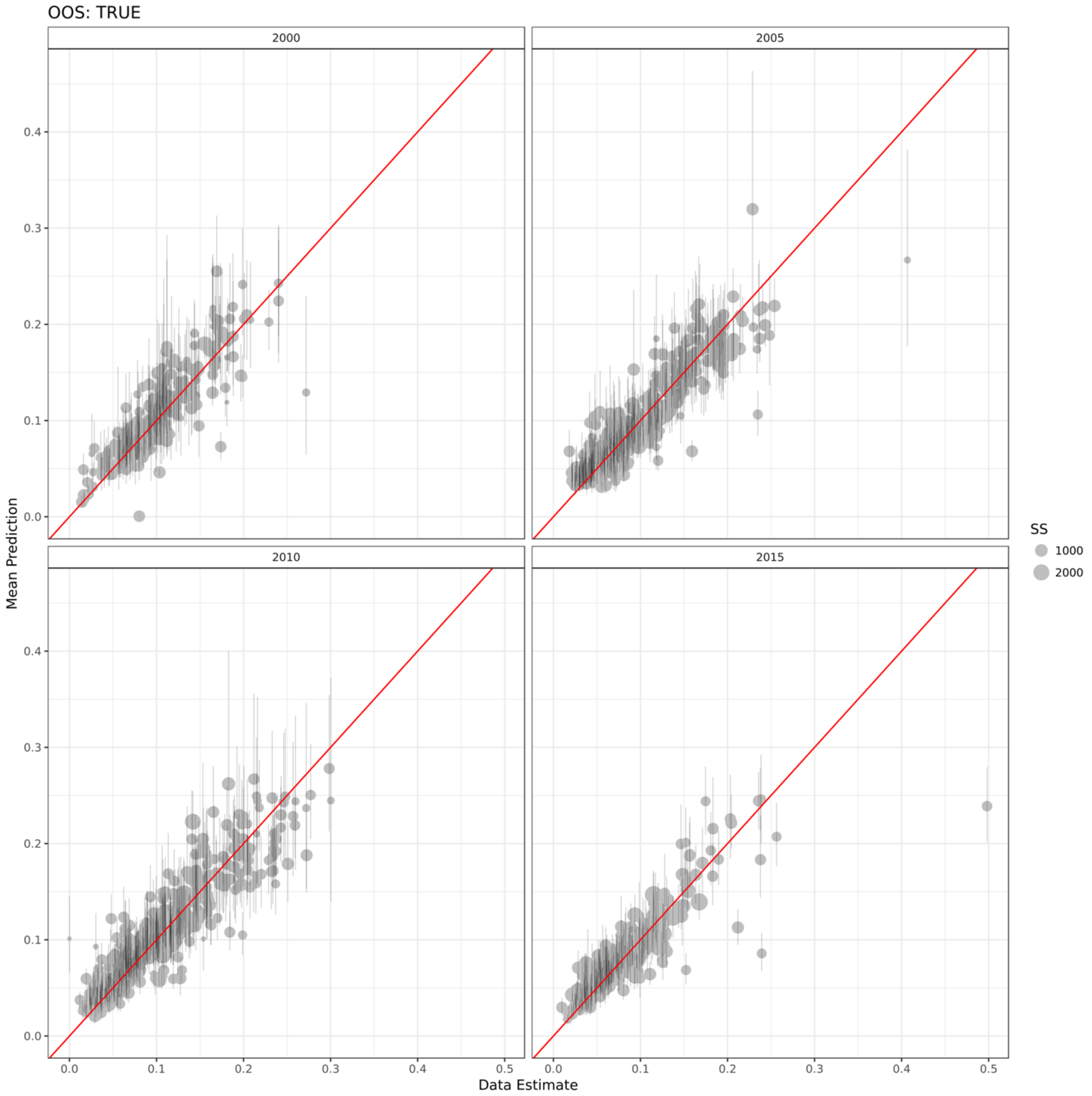

Supplementary Figure 30. Wasting holdout units aggregation out-of-sample. Comparison of out-of-sample wasting predictions aggregated to holdout units generated from recursive quad-tree with 95% uncertainty intervals plotted against aggregated data observations from the same spatial region.

5.4.6 Underweight validation metrics

Supplementary Table 16. Predictive metrics for underweight aggregated to admin 0.
The out-of-sample (OOS) column indicates whether the metric was calculated using in-sample or out-of-sample predictions.

Year	OOS	Median SS	Mean err.	RMSE	CoV (%)	Corr.	95% Cov.
**2000**	FALSE	4424.000	4424.000	-0.003	0.014	5.638	0.991
**2005**	FALSE	4837.000	4837.000	-0.003	0.012	5.584	0.990
**2010**	FALSE	7074.000	7074.000	-0.006	0.009	4.614	0.996
**2015**	FALSE	5657.082	5657.082	-0.003	0.010	5.429	0.992

Supplementary Table 17. Predictive metrics for underweight aggregated to admin 1

Year	OOS	Median SS	Mean err.	RMSE	CoV (%)	Corr.	95% Cov.
**2000**	FALSE	214.000	-0.003	0.029	12.076	0.967	0.972
**2005**	FALSE	312.500	-0.003	0.024	11.366	0.970	0.955
**2010**	FALSE	364.062	-0.006	0.021	10.496	0.982	0.951
**2015**	FALSE	388.326	-0.003	0.024	13.386	0.972	0.956

Supplementary Table 18. Predictive metrics for underweight aggregated to admin 2

Year	OOS	Median SS	Mean err.	RMSE	CoV (%)	Corr.	95% Cov.
**2000**	FALSE	27.774	-0.003	0.041	16.930	0.939	0.972
**2005**	FALSE	33.567	-0.003	0.040	18.749	0.926	0.954
**2010**	FALSE	43.969	-0.006	0.042	20.774	0.933	0.951
**2015**	FALSE	36.849	-0.003	0.054	29.737	0.889	0.957

Supplementary Table 19. Predictive metrics for underweight aggregated to holdout units

Year	OOS	Median SS	Mean err.	RMSE	CoV (%)	Corr.	95% Cov.
**2000**	FALSE	550.987	-0.003	0.020	8.465	0.978	0.972
**2005**	FALSE	641.625	-0.003	0.021	9.623	0.974	0.954
**2010**	FALSE	713.000	-0.006	0.018	8.631	0.984	0.951
**2015**	FALSE	807.000	-0.003	0.019	10.629	0.974	0.957
**2000**	TRUE	550.987	-0.003	0.032	13.472	0.943	0.947
**2005**	TRUE	641.625	-0.003	0.029	13.673	0.946	0.945
**2010**	TRUE	713.000	-0.006	0.029	14.064	0.953	0.930
**2015**	TRUE	807.000	-0.001	0.030	16.657	0.934	0.948

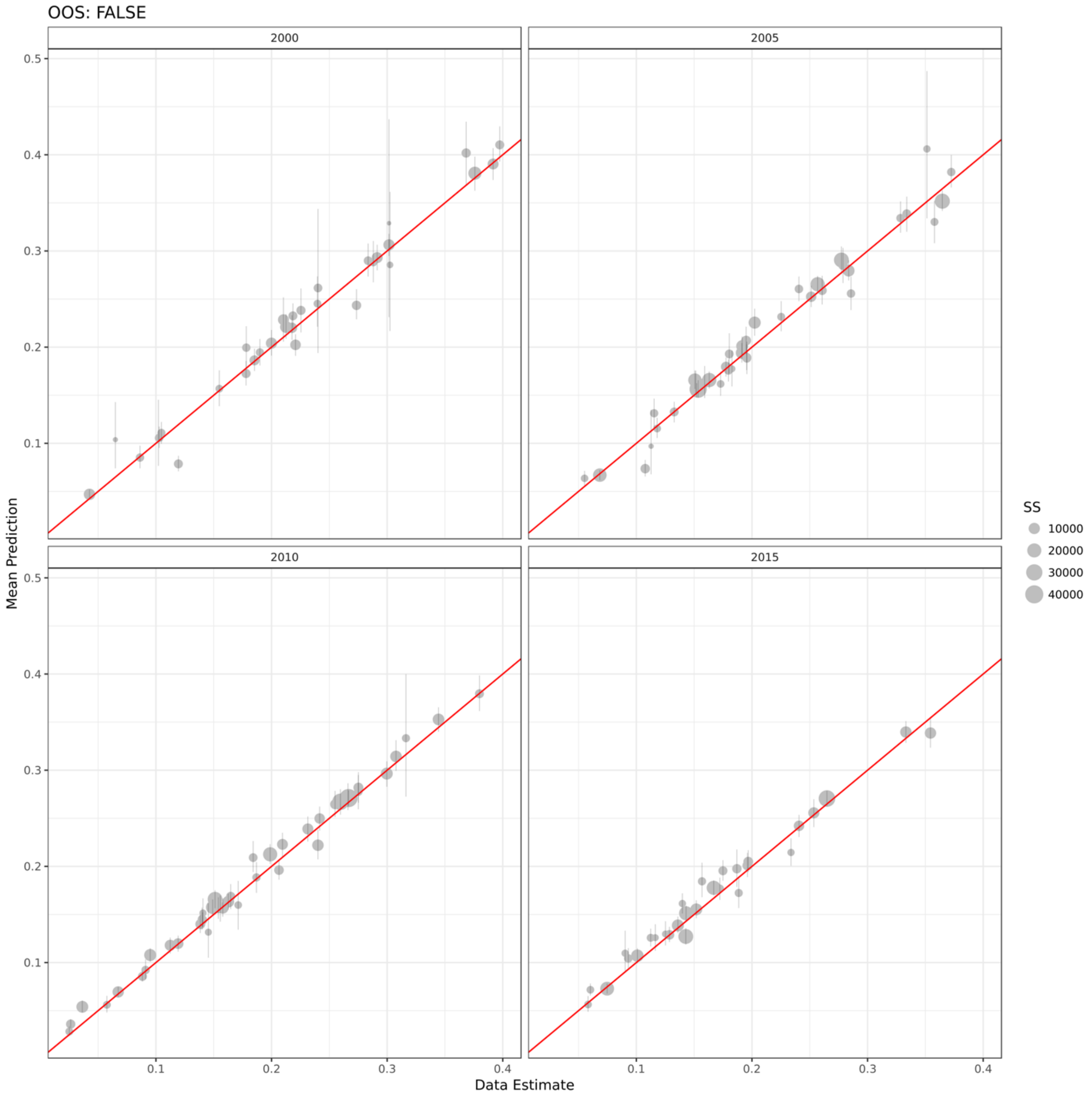

Supplementary Figure 31. Underweight admin 0 aggregation in-sample.
Comparison of in-sample underweight predictions aggregated to admin 0 with 95% uncertainty intervals plotted against admin 0 aggregated data observations.

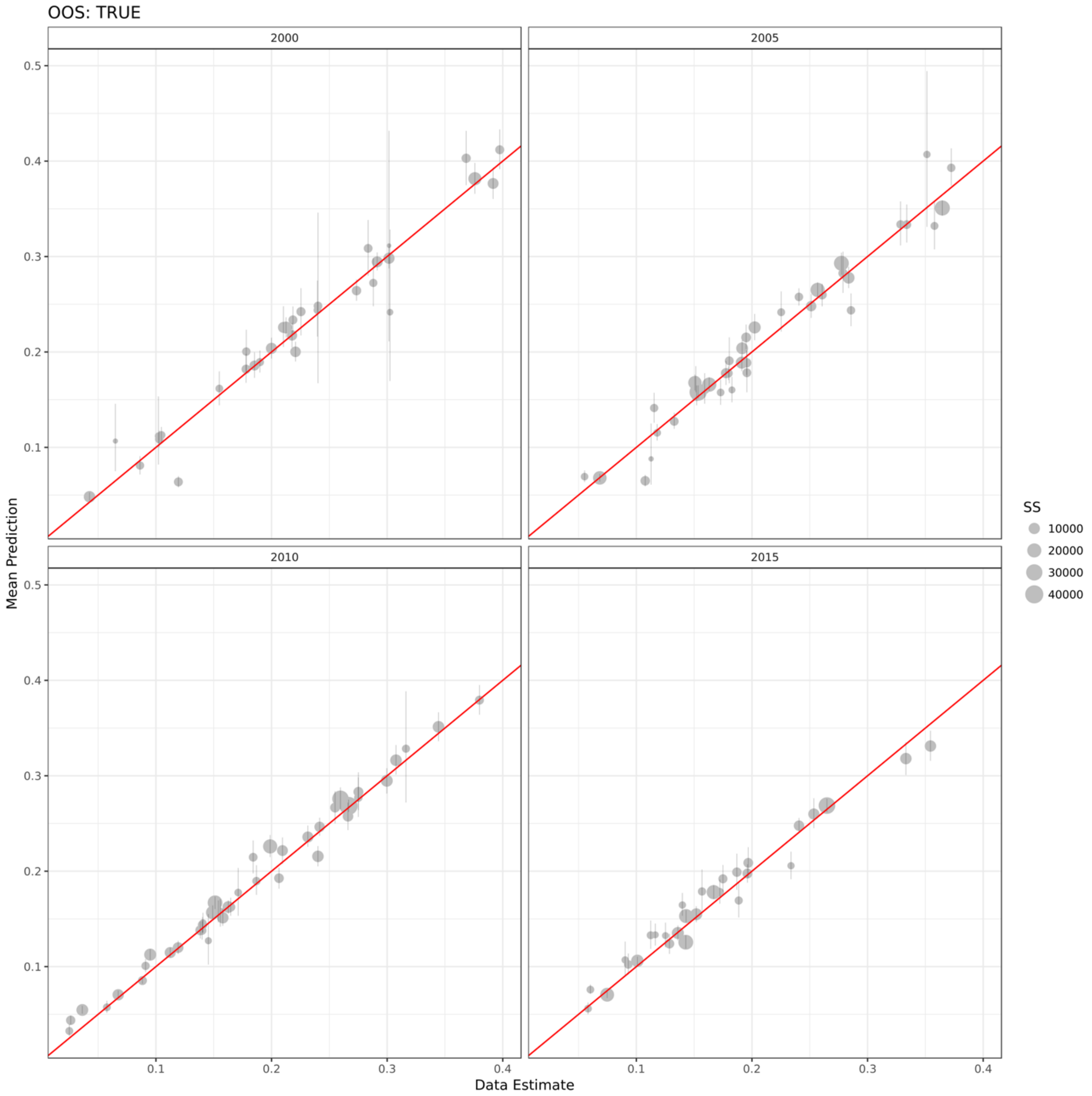

Supplementary Figure 32. Underweight admin 0 aggregation out-of-sample. Comparison of out-of-sample underweight predictions aggregated to admin 0 with 95% uncertainty intervals plotted against admin 0 aggregated data observations.

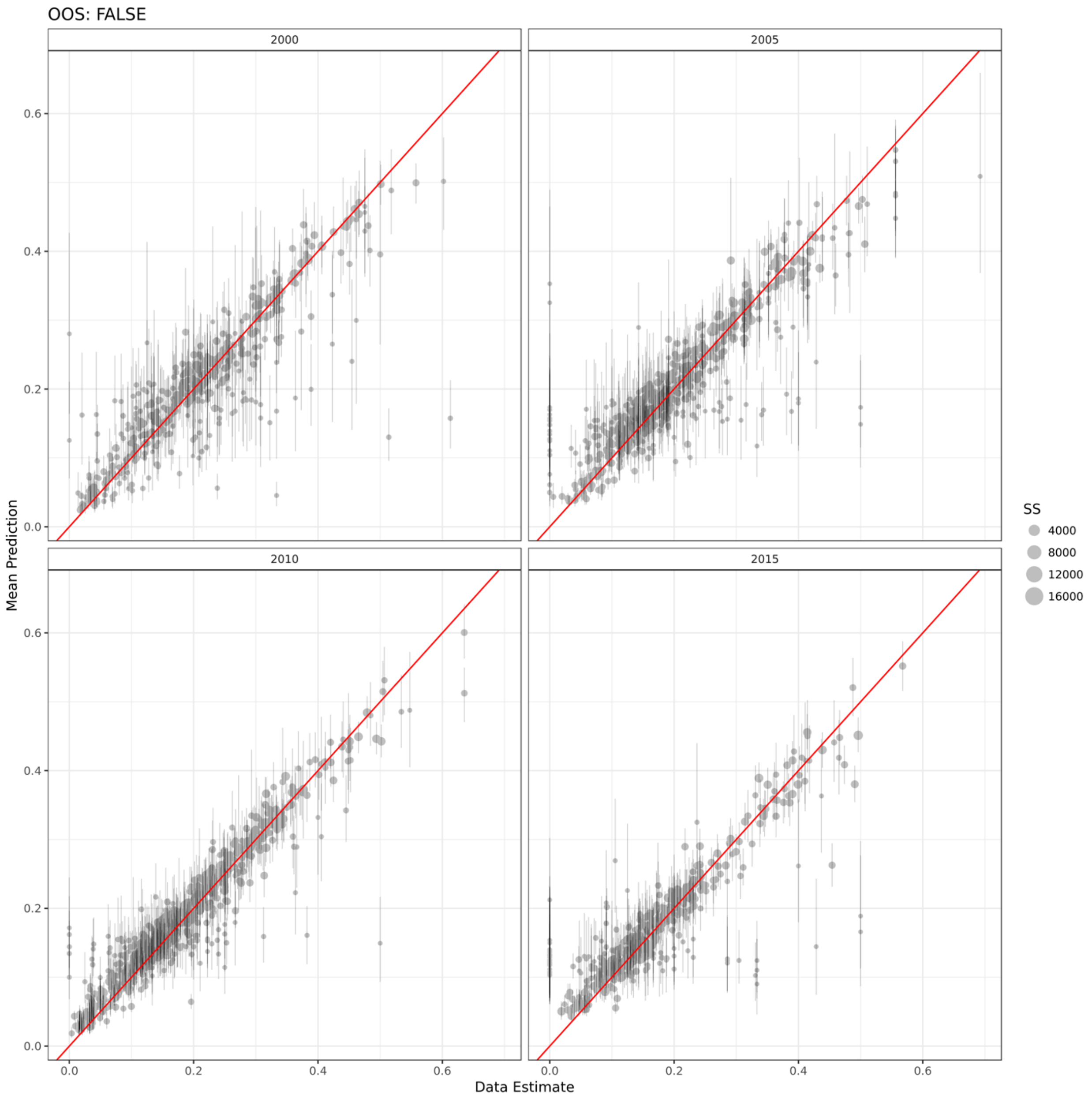

Supplementary Figure 33. Underweight admin 1 aggregation in-sample.
Comparison of in-sample underweight predictions aggregated to admin 1 with 95% uncertainty intervals plotted against admin 1 aggregated data observations.

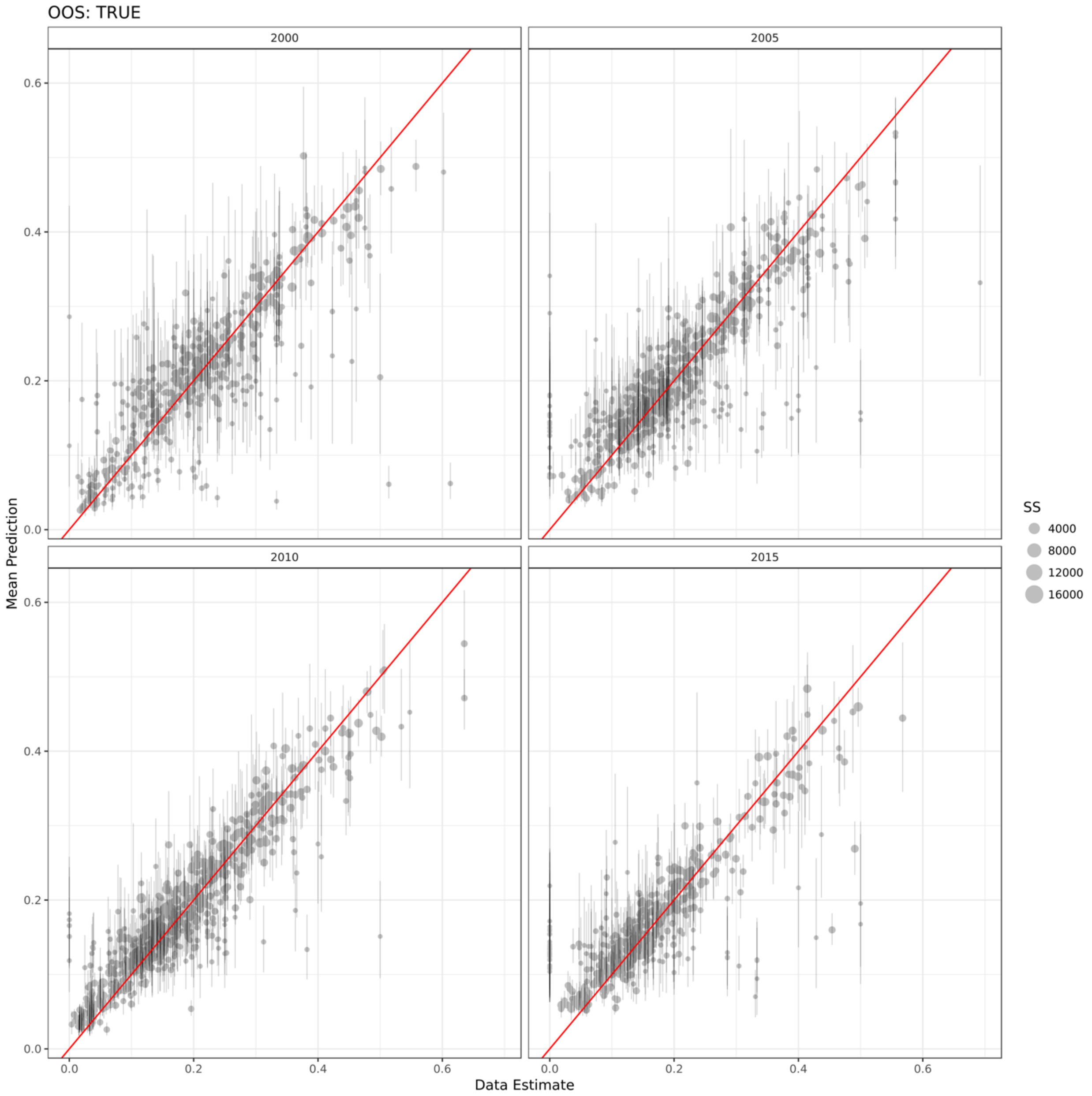

Supplementary Figure 34. Underweight admin 1 aggregation out-of-sample. Comparison of out-of-sample underweight predictions aggregated to admin 1 with 95% uncertainty intervals plotted against admin 1 aggregated data observations.

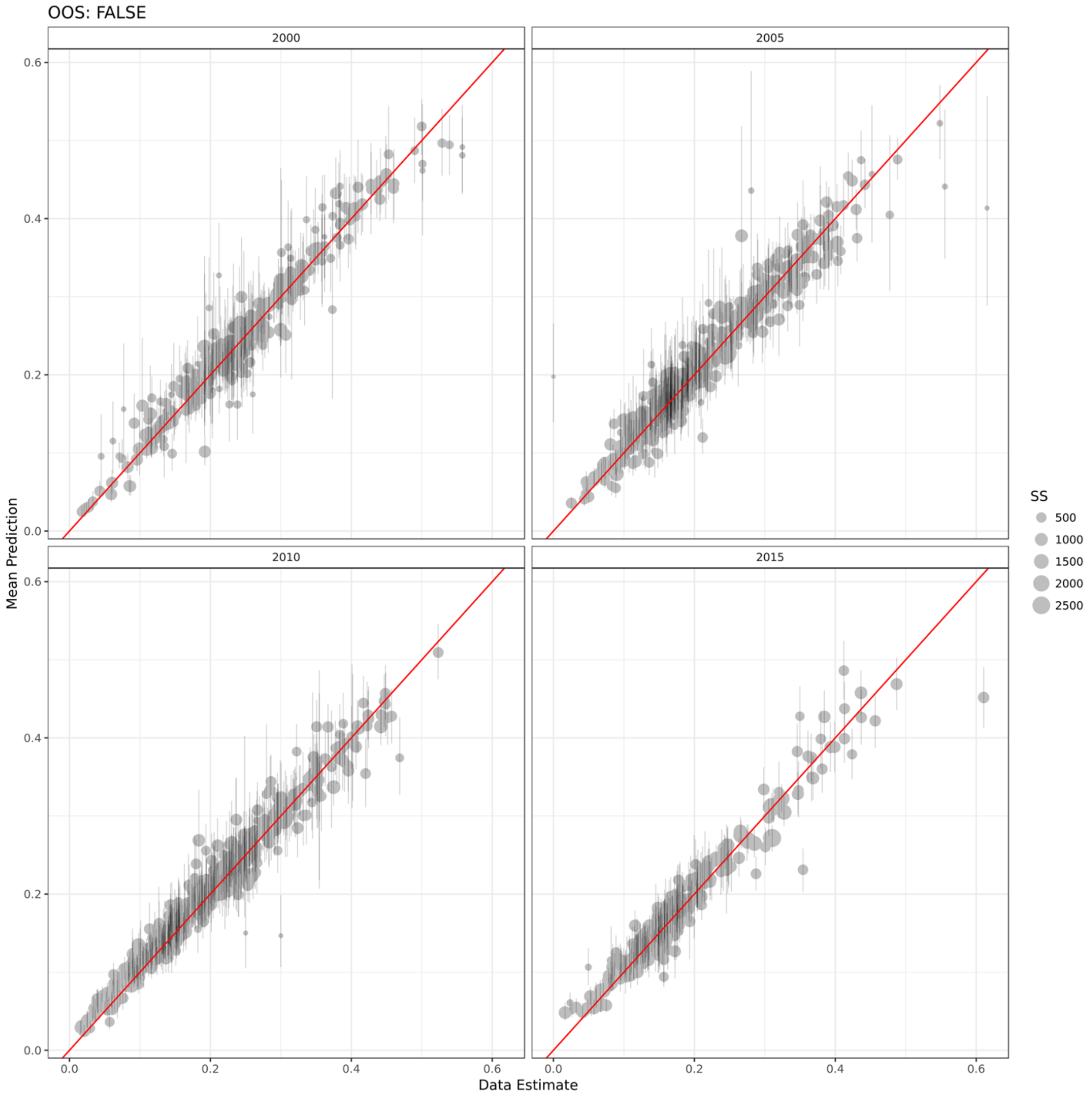

Supplementary Figure 35. Underweight holdout units aggregation in-sample.
Comparison of in-sample predictions aggregated to holdout units generated from recursive quad-tree with 95% uncertainty intervals plotted against aggregated data observations from the same spatial region.

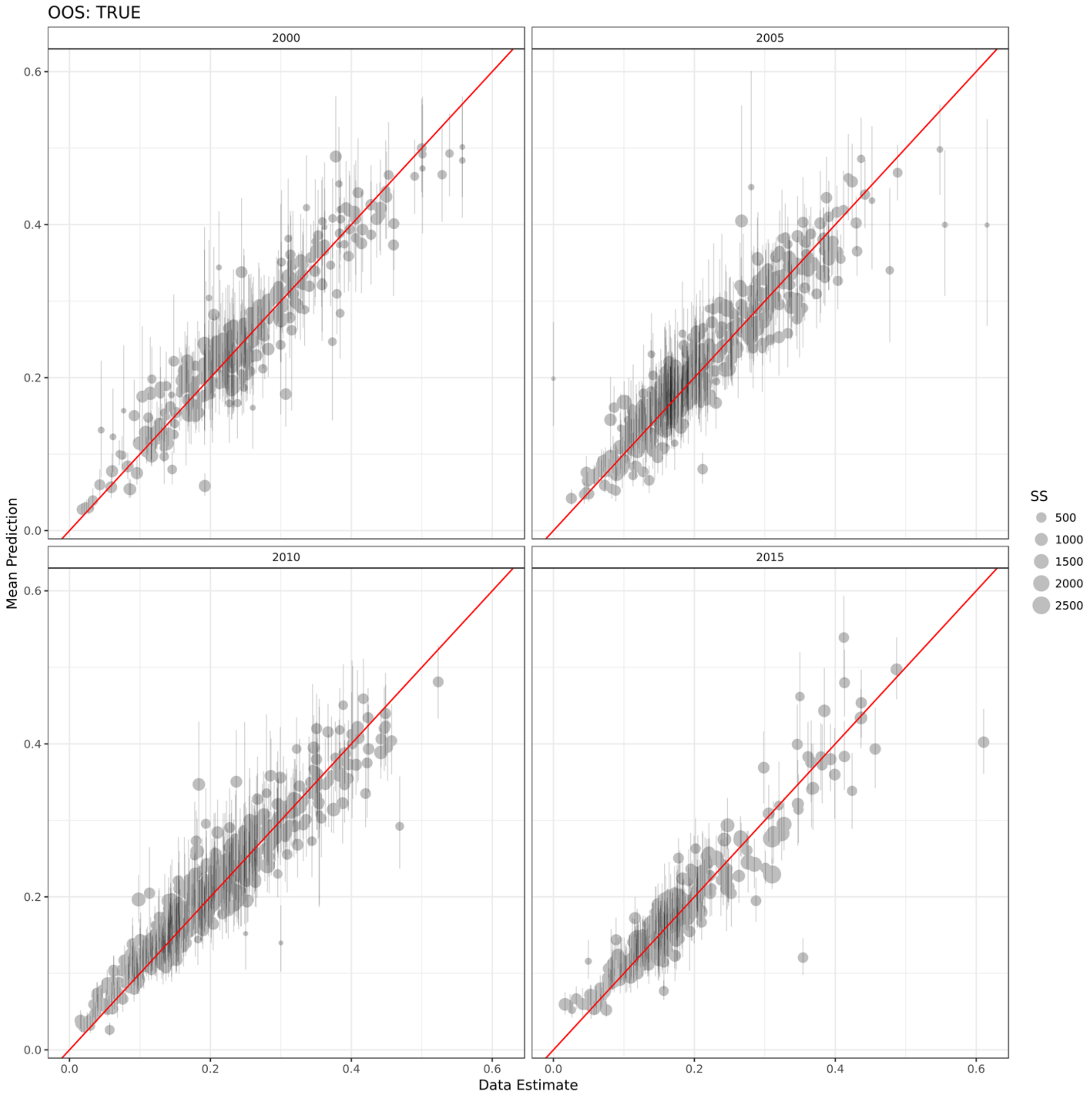

Supplementary Figure 36. Underweight holdout units aggregation out-of-sample. Comparison of out-of-sample predictions aggregated to holdout units generated from recursive quad-tree with 95% uncertainty intervals plotted against aggregated data observations from the same spatial region.

5.4.7 Absolute error plots

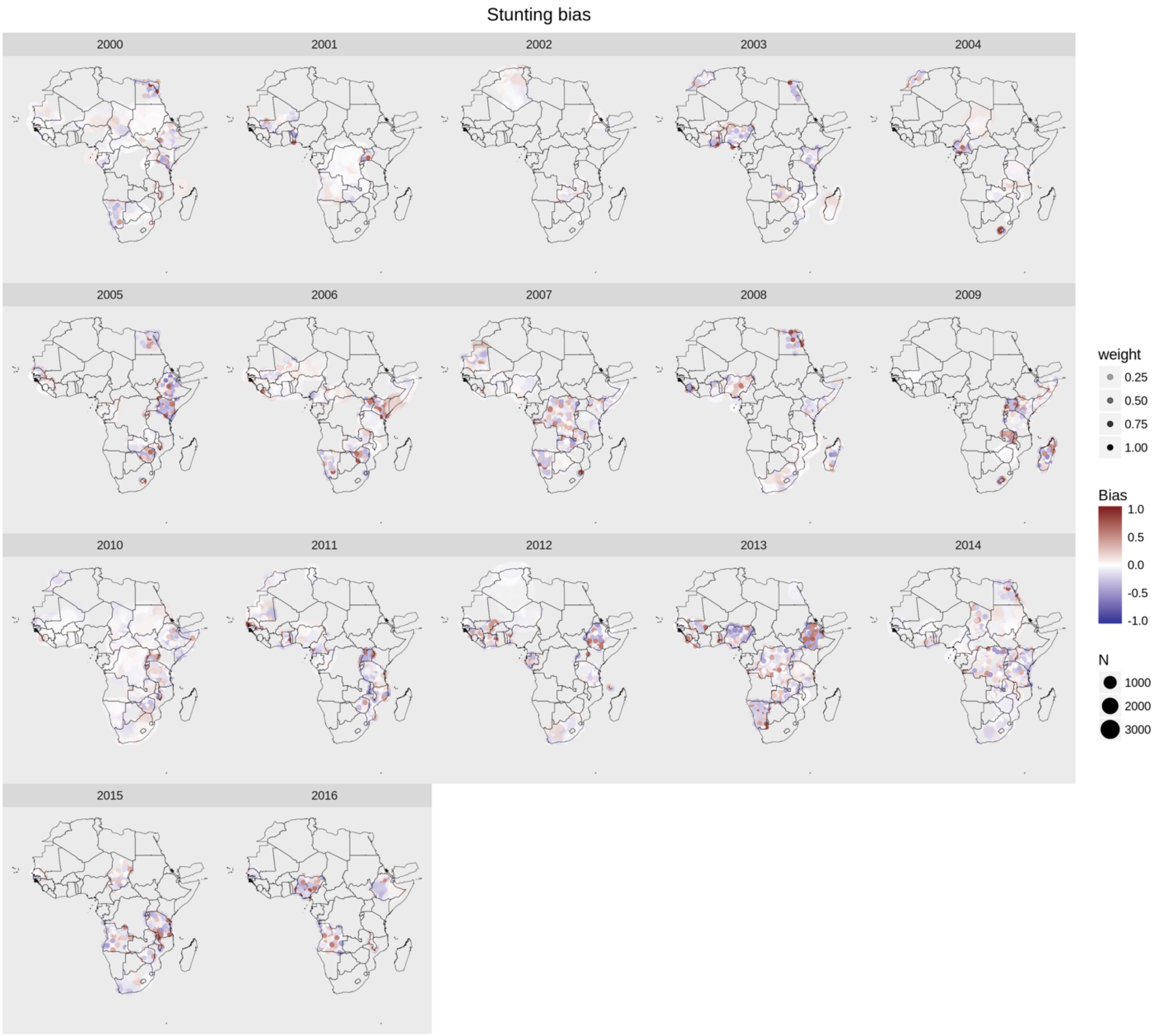

Supplementary Figure 37. Plots of stunting bias in Africa out-of-sample.
Colour indicates magnitude of out-of-sample error, size of the points represents the sample size of the observed cluster or pseudocluster, and transparency represents the weight of the cluster or pseudocluster.

doi:10.1038/nature25760

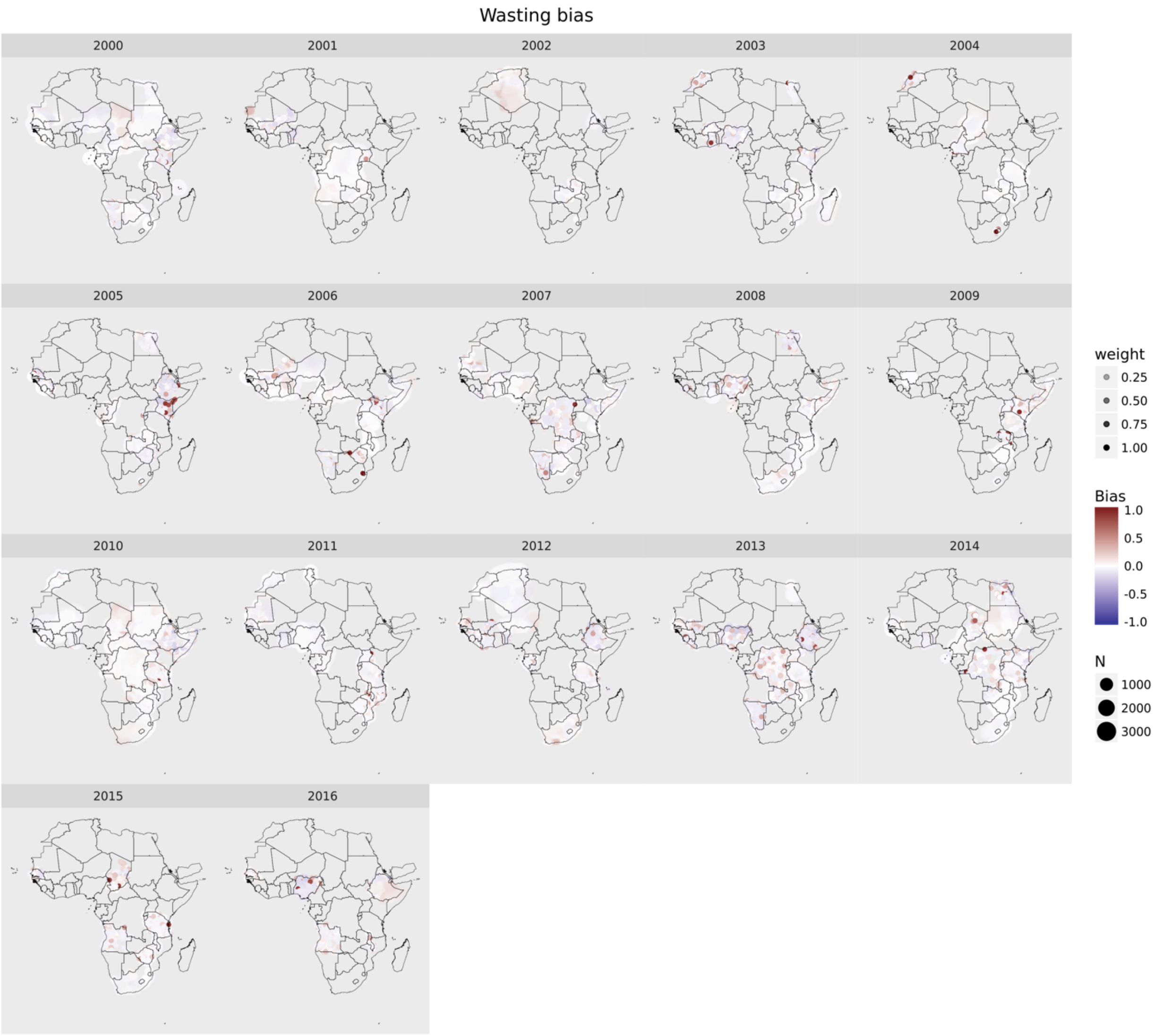

Supplementary Figure 38. Plots of wasting bias in Africa out-of-sample.

Colour indicates magnitude of out-of-sample error, size of the points represents the sample size of the observed cluster or pseudocluster, and transparency represents the weight of the cluster or pseudocluster.

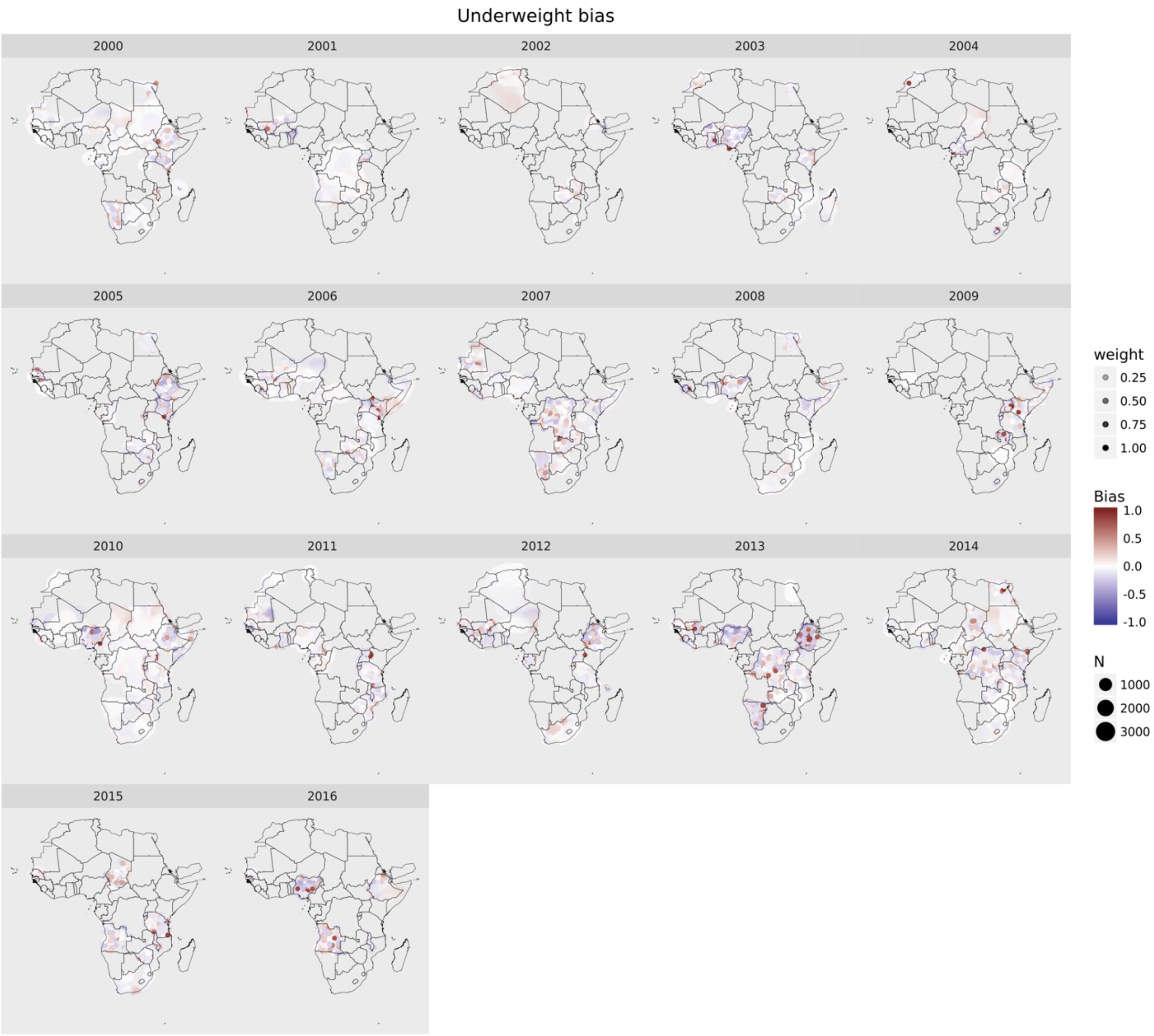

Supplementary Figure 39. Plots of underweight bias in Africa out-of-sample.

Colour indicates magnitude of out-of-sample error, size of the points represents the sample size of the observed cluster or pseudocluster, and transparency represents the weight of the cluster or pseudocluster.

5.4.8 Post estimation calibration to national estimates

In order to leverage national-level data included in GBD 2016, but outside the scope of our current geospatial modelling framework, and to ensure perfect calibration between these estimates and GBD 2016 national-level estimates, we performed a post hoc calibration to each of our 1,000 candidate maps.[11] For each posterior draw we calculated population-weighted pixel aggregations to a national-level and compared these country-year estimates to the analogous and available GBD 2016[11] country-years (all countries for 2000, 2005, 2010, and 2016). To generate 2015 national-level estimates for use in calibrating our 2015 5x5 km maps, we linearly interpolated between 2010 and 2016 estimates. We defined the raking factor to be the ratio between the GBD 2016[11] estimate and our current estimates and linearly interpolated raking factors in a country between the available years. Finally, we multiplied each of our pixels in a country-year by its associated raking factor. This ensures perfect calibration between our geospatial estimates and GBD 2016[11] national-level estimates, while preserving our estimated within-country geospatial and temporal variation.

To allow comparison between our modelled estimates and the GBD 2016[11] national-level estimates to which they were calibrated, Supplementary Fig. 28-30 plot mean uncalibrated estimates from the model-based geostatistics (MBG) process aggregated to the national-level ("MBG mean") as compared to the GBD national estimates ("GBD mean") for all modelled years. The median raking factors for stunting, wasting, and underweight were 0.983 (interquartile range: 0.917-1.052), 1.055 (IQR: 0.947-1.207), and 0.985 (IQR: 0.903-1.064), respectively.

doi:10.1038/nature25760

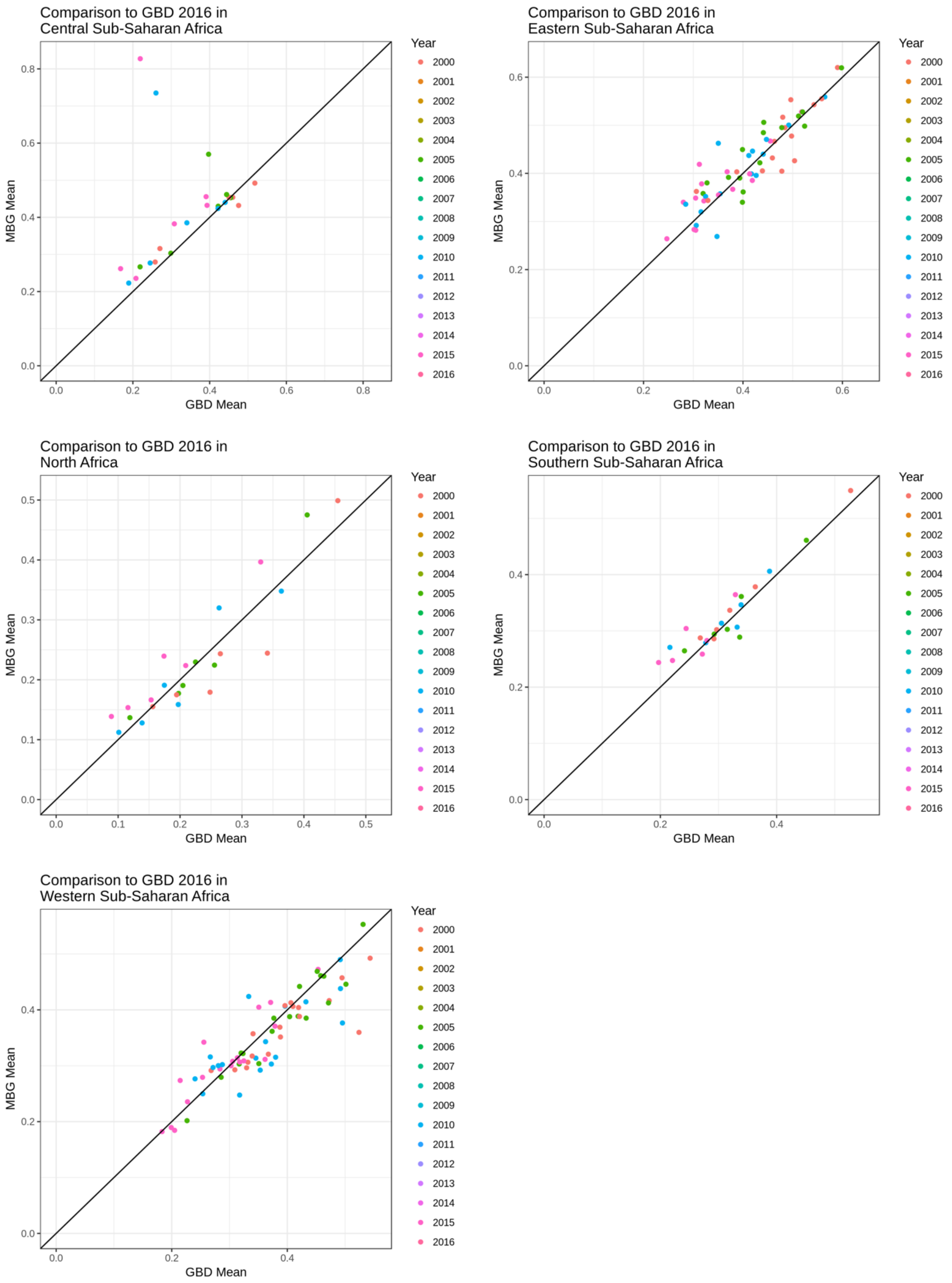

Supplementary Figure 40. Comparison of aggregated stunting MBG estimates to GBD 2016 stunting estimates.

Note that our models and GBD 2016 datasets overlap but are not identical.

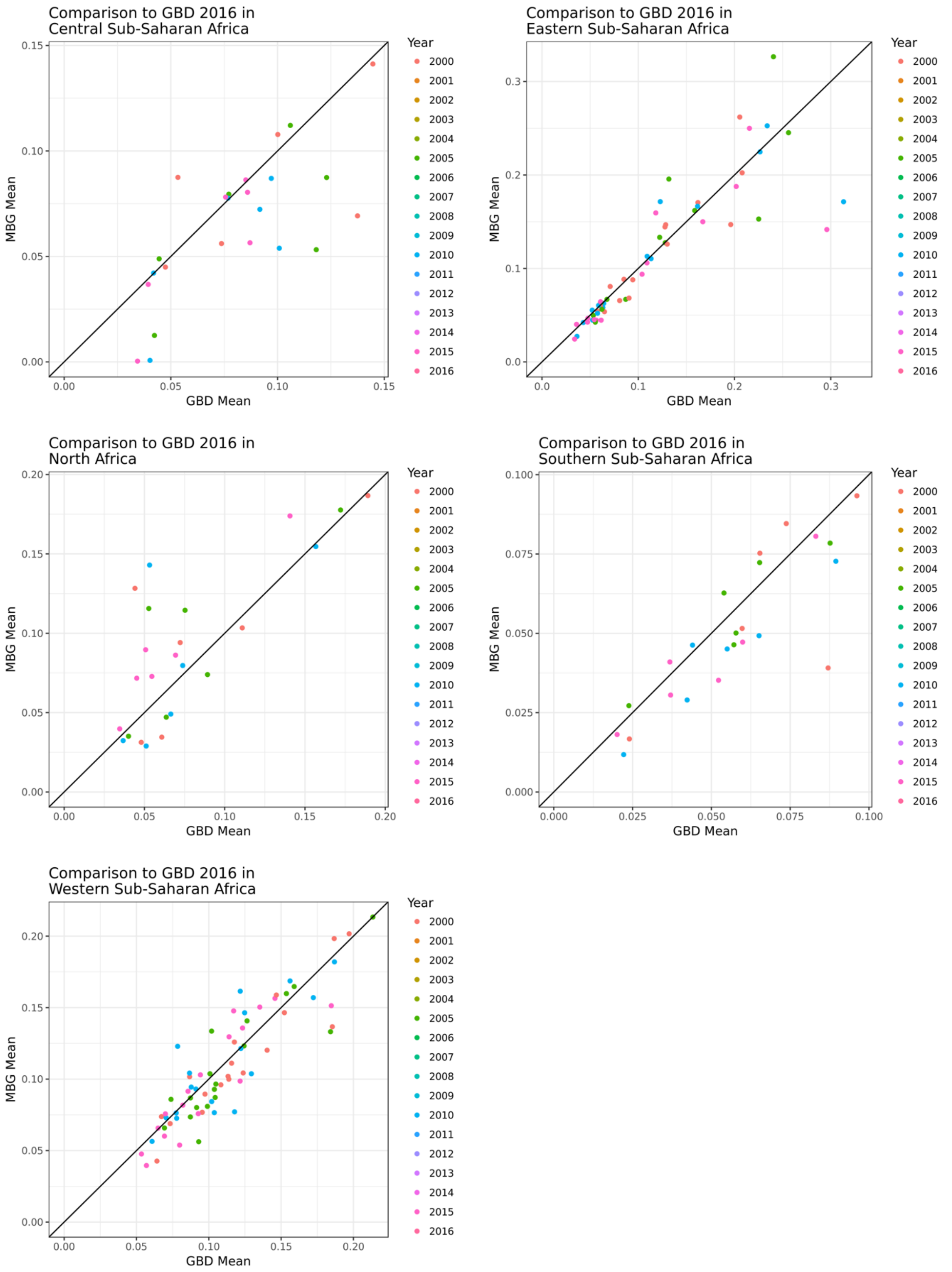

Supplementary Figure 41. Comparison of aggregated wasting MBG estimates to GBD 2016 stunting estimates.
Note that our models and GBD 2016 datasets overlap but are not identical.

doi:10.1038/nature25760

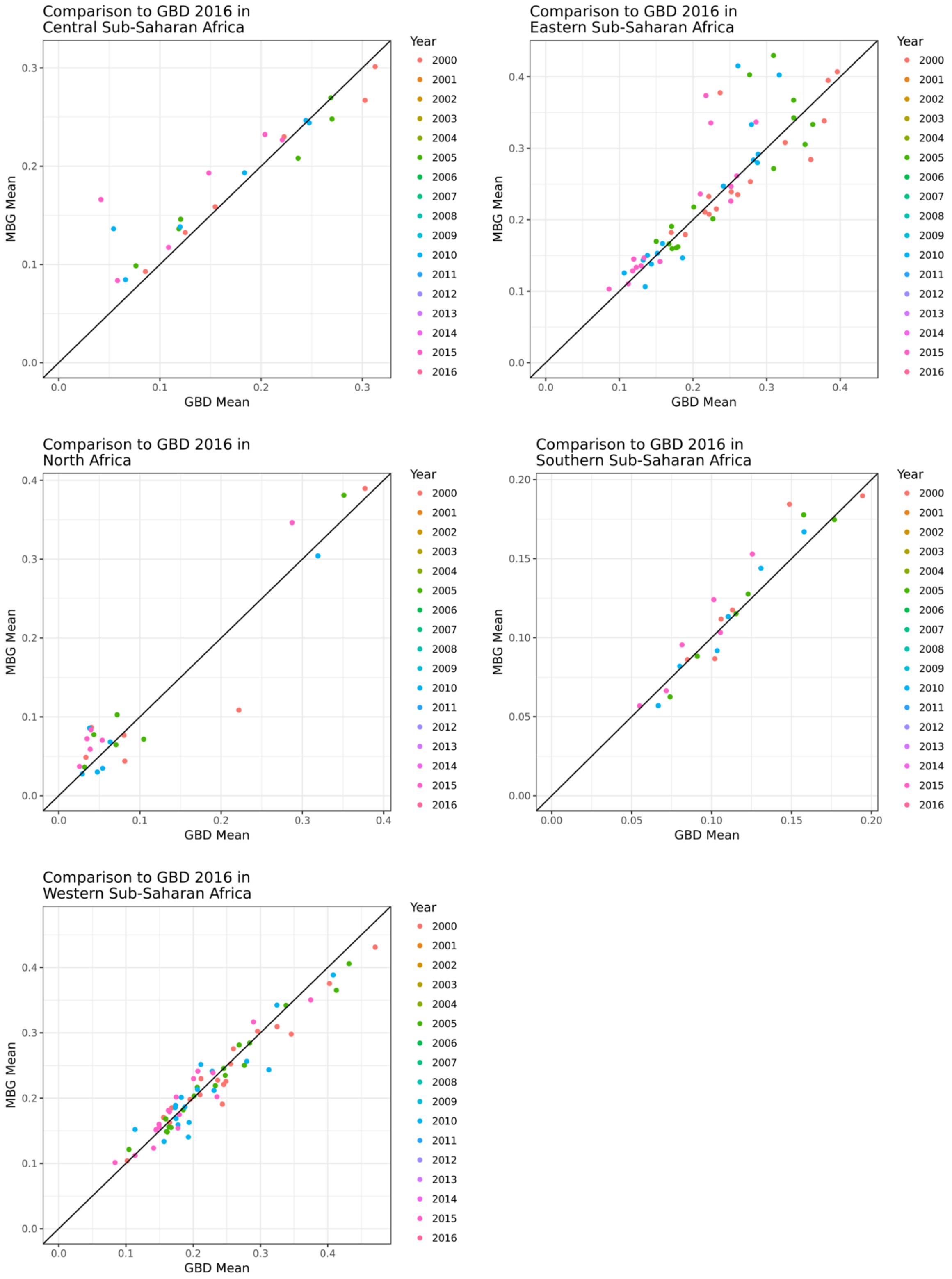

Supplementary Figure 42. Comparison of aggregated underweight MBG estimates to GBD 2016 stunting estimates.
Note that our models and GBD 2016 datasets overlap but are not identical.

5.4.9 Verification and comparison against other subnational CGF estimates

Estimates produced by MBG models were compared to raw estimates from the DHS series, each aggregated to the first subnational geographic subdivision. These results are presented below.

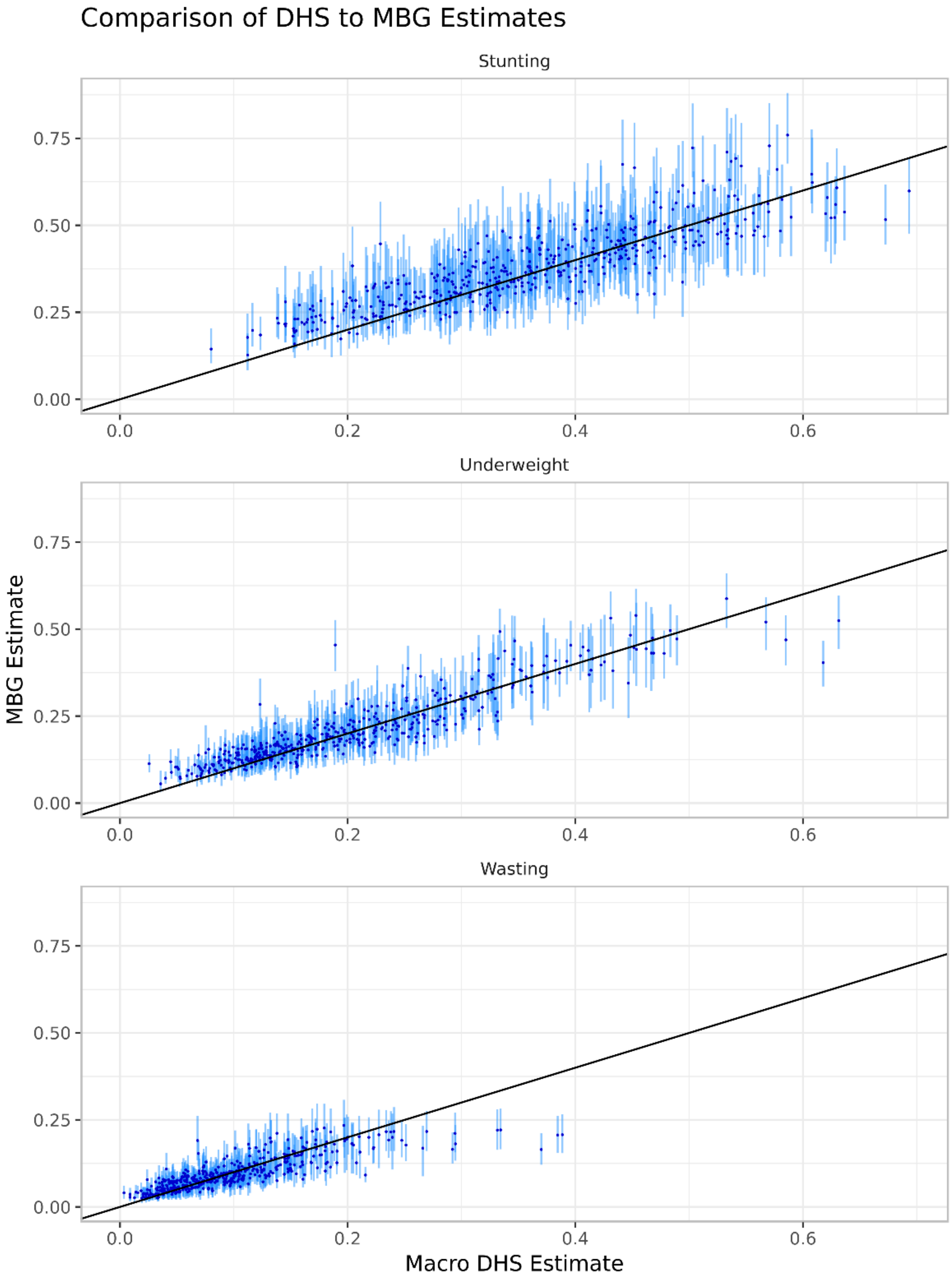

Supplementary Figure 43. Comparison of MBG estimates aggregated to admin 1 to DHS admin 1 estimates.
95% uncertainty intervals are plotted along with the aggregated MBG estimates. Note that our model includes more data than just the DHS surveys.

5.4.10 Evaluation of projection methodology

In the interest of evaluating our projection methods (described in the Methods section in the main manuscript), annual rates of change at the pixel level were calculated based on the years 2000 to 2010, and were applied to the 2010 estimate to generate a "projection" for 2015. The alignment between this projection and our estimates is shown below for each indicator. Although the projections often yield reasonably close comparisons with the 2015 predictions, this is only true as long as the rates of change in the past are consistent with what was observed. An example where this does not hold can be seen in northern Nigeria in the stunting projection figure.

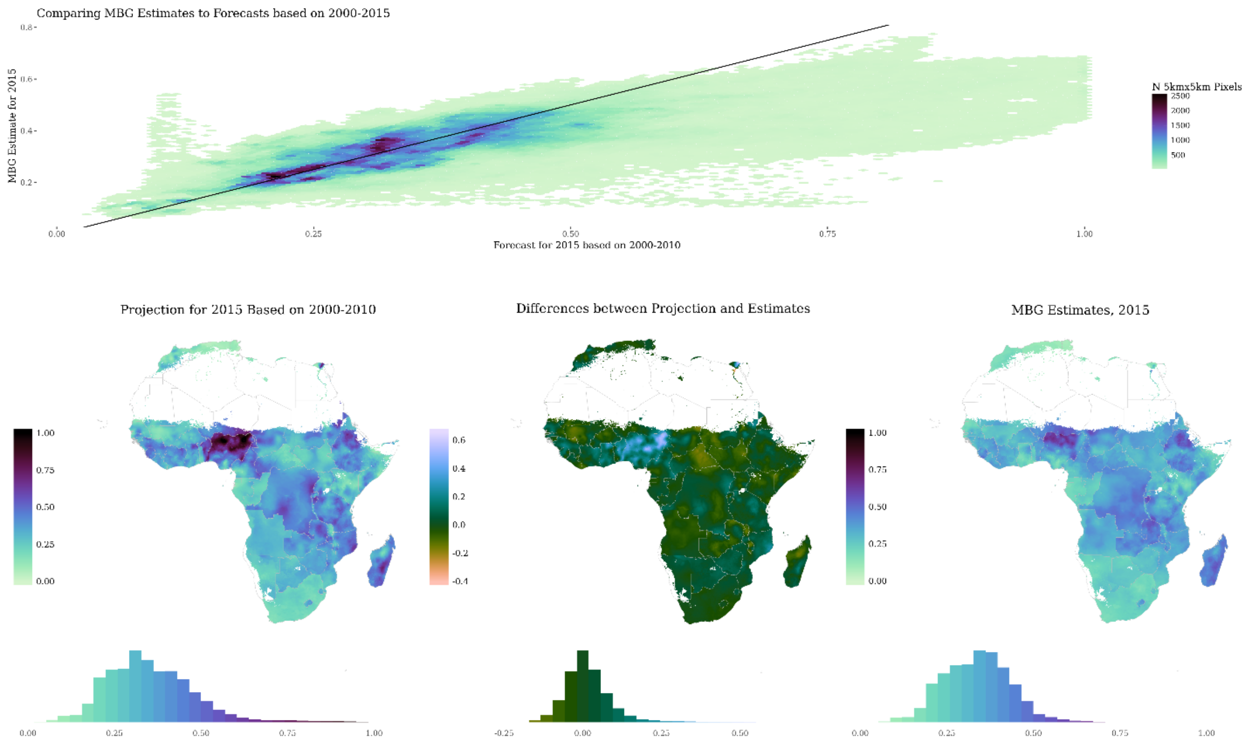

Supplementary Figure 44. Stunting projection comparison.
Comparison of 2015 estimates for stunting to "projected estimates" generated using annual rates of change based on time period of 2000–2010. The leftmost map shows the projections for 2015 using a 5-year projection starting from 2010 and weighted annualized rates of change calculated from the 2000-2010 estimated mean maps. The rightmost plot shows the 2015 estimates and the middle map plots the difference between the two. The scatterplot on top plots the 2015 projected pixels against the estimated 2015 pixels from the model run.

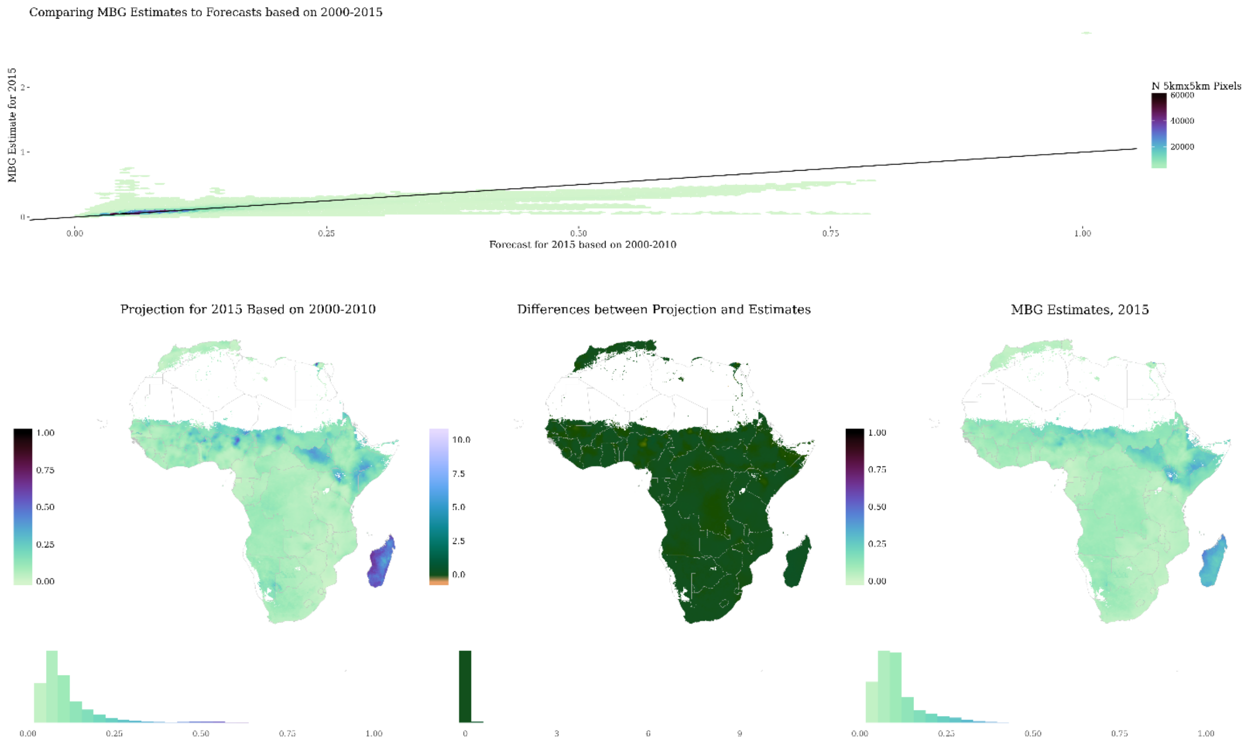

Supplementary Figure 45. Wasting projection comparison.
Comparison of 2015 estimates for underweight to "projected estimates" generated using annual rates of change based on time period of 2000–2010. The leftmost map shows the projections for 2015 using a 5-year projection starting from 2010 and weighted annualized rates of change calculated from the 2000-2010 estimated mean maps. The rightmost plot shows the 2015 estimates and the middle map plots the difference between the two. The scatterplot on top plots the 2015 projected pixels against the estimated 2015 pixels from the model run.

doi:10.1038/nature25760

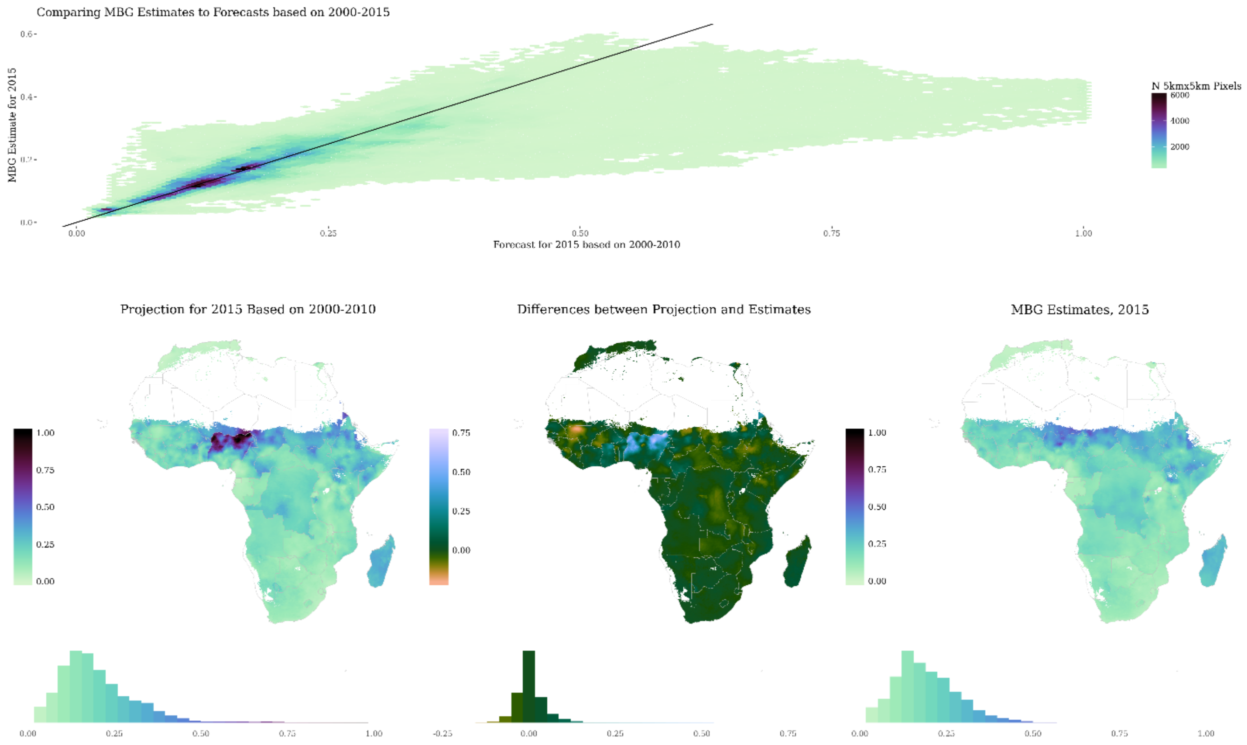

Supplementary Figure 46. Underweight projection comparison.
Comparison of 2015 estimates for wasting to "projected estimates" generated using annual rates of change based on time period of 2000–2010. The leftmost map shows the projections for 2015 using a 5-year projection starting from 2010 and weighted annualized rates of change calculated from the 2000-2010 estimated mean maps. The rightmost plot shows the 2015 estimates and the middle map plots the difference between the two. The scatterplot on top plots the 2015 projected pixels against the estimated 2015 pixels from the model run.

6.0 Supplementary references

1. USAID. *The 1,000-day Window of Opportunity: Technical Guidance Brief.*
2. Toe, L. C. *et al.* Seasonality modifies the effect of a lipid-based nutrient supplement for pregnant rural women on birth length. *J. Nutr.* **145,** 634–639 (2015).
3. Bhutta, Z. A. *et al.* Evidence-based interventions for improvement of maternal and child nutrition: what can be done and at what cost? *The Lancet* **382,** 452–477 (2013).
4. WHO. *Management of severe malnutrition: a manual for physicians and other senior health workers*. (WHO, 1999).
5. WHO. *Management of the child with a serious infection or severe malnutrition: guidelines for care at the first-referral level in developing countries*. (WHO, 2000).
6. Collins, S. Treating severe acute malnutrition seriously. *Arch. Dis. Child.* **92,** 453–461 (2007).
7. Lenters, L. M., Wazny, K., Webb, P., Ahmed, T. & Bhutta, Z. A. Treatment of severe and moderate acute malnutrition in low- and middle-income settings: a systematic review, meta-analysis and Delphi process. *BMC Public Health* **13,** S23 (2013).
8. Downs, R. E. (ed), Kerner, D. O. (ed) & Reyna, S. P. (ed). *The Political Economy of African Famine*. (Philadelphia Penn.: Gordan and Breach Science Publishers 1991, 2016).
9. Kassebaum, N. J. *et al.* Global, regional, and national disability-adjusted life-years (DALYs) for 315 diseases and injuries and healthy life expectancy (HALE), 1990–2015: a systematic analysis for the Global Burden of Disease Study 2015. *The Lancet* **388,** 1603–1658 (2016).
10. Sahn, D. E. *The fight against hunger and malnutrition: the role of food, agriculture, and targeted policies*. (OUP Oxford, 2015).
11. Gakidou, E. *et al.* Global, regional, and national comparative risk assessment of 84 behavioural, environmental and occupational, and metabolic risks or clusters of risks, 1990–2016: a systematic analysis for the Global Burden of Disease Study 2016. *The Lancet* **390,** 1345–1422 (2017).
12. Lumley, T. Analysis of complex survey samples. *J. Stat. Softw.* **9,** (2004).
13. Gelman, A. Struggles with survey weighting and regression modeling. *Stat. Sci.* **22,** 153164 (2007).
14. Kish, L. *Survey Sampling*. (Wiley-Interscience, 1995).
15. Golding, N., Burstein, R., Longbottom, J. & et al. Mapping under-5 and neonatal mortality in Africa, 2000–2015: a baseline analysis for the Sustainable Development Goals. *Lancet Press* (2017).

16. Lloyd, C. T., Sorichetta, A. & Tatem, A. J. High resolution global gridded data for use in population studies. *Sci. Data* **4,** sdata20171 (2017).

17. World Pop. Get data. Available at: http://www.worldpop.org.uk/data/get_data/. (Accessed: 25th July 2017)

18. Murray, C. J. *et al.* GBD 2010: design, definitions, and metrics. *The Lancet* **380,** 2063–2066 (2012).

19. Stein, M. L. *Interpolation of Spatial Data*. (Springer New York, 1999).

20. Waller, L. & Carlin, B. Disease mapping. in *Handbook of Spatial Statistics* 217–243 (CRC Press, 2010).

21. Rue, H., Martino, S. & Chopin, N. Approximate Bayesian inference for latent Gaussian models by using integrated nested Laplace approximations. *J. R. Stat. Soc. Ser. B Stat. Methodol.* **71,** 319–392 (2009).

22. Martins, T. G., Simpson, D., Lindgren, F. & Rue, H. Bayesian computing with INLA: new features. *Comput Stat Data Anal* **67,** 68–83 (2013).

23. Lindgren, F., Rue, H. & Lindström, J. An explicit link between Gaussian fields and Gaussian Markov random fields: the stochastic partial differential equation approach. *J. R. Stat. Soc. Ser. B Stat. Methodol.* **73,** 423–498 (2011).

24. Patil, A. P., Gething, P. W., Piel, F. B. & Hay, S. I. Bayesian geostatistics in health cartography: the perspective of malaria. *Trends Parasitol.* **27,** 246–253 (2011).

25. Rue, H., Martino, S. & Chopin, N. Approximate Bayesian inference for latent Gaussian models by using integrated nested Laplace approximations. *J. R. Stat. Soc. Ser. B Stat. Methodol.* **71,** 319–392 (2009).

26. Roberts, D. R. *et al.* Cross-validation strategies for data with temporal, spatial, hierarchical, or phylogenetic structure. *Ecography* n/a-n/a (2017). doi:10.1111/ecog.02881

27. FAO. The Global Administrative Unit layers (GAUL): Technical Aspects. Available at: http://www.fao.org/geonetwork/srv/en/main.home.
